# Comparison of Protein–Glycosaminoglycan Interactions in ff14sb/GLYCAM06j‑1 and CHARMM36m Force Fields

**DOI:** 10.1021/acs.jcim.5c03159

**Published:** 2026-05-04

**Authors:** Krzysztof K. Bojarski, Patryk A. Wesołowski, Diksha Dewan, Łukasz J. Dziadek, Vilmos Neuman, Bernard R. Brooks, Jacek Czub, Martin Zacharias, Adam K. Sieradzan, David J. Wales

**Affiliations:** † Department of Physical Chemistry, 49646 Gdansk University of Technology, Narutowicza 11/12, Gdansk 80-308, Poland; ‡ Center for Functional Protein Assemblies, 9184 Technical University of Munich, Ernst-Otto-Fischer-Straße 8, Garching 85748, Germany; § Yusuf Hamied Department of Chemistry, 2152 University of Cambridge, Lensfield Road, Cambridge CB2 1EW, U.K.; ∥ Department of Theoretical Chemistry, University of Gdansk, Wita Stwosza 63, Gdansk 80-952, Poland; ⊥ Department of Chemistry, Physical and Theoretical Chemistry Laboratory, 150602 University of Oxford, South Parks Road, Oxford OX1 3QZ, U.K.; # Laboratory of Computational Biology, National Heart Lung and Blood Institute, 2511 National Institutes of Health, Bethesda, Maryland 20892, United States; ∇ Physics Department, Technical University of Munich, James-Franck Strasse 1, Garching 85748, Germany

## Abstract

Glycosaminoglycans (GAGs) are long, anionic polysaccharides abundant in the extracellular matrix and lysosomes, where their electrostatic interactions with proteins are essential for biological function. Computational studies of GAG-containing systems remain challenging due to their significant charge density and conformational flexibility. Here we benchmark two widely used force-fields, ff14SB/GLYCAM06j-1 and CHARMM36m, for three experimentally characterized protein–GAG complexes. Both force fields reproduce the key structural features of protein–GAG interactions, while GAG dynamics depend on protein charge, with CHARMM36m favoring broader surface exploration for highly positively charged proteins and AMBER enhancing mobility for less charged systems. Although protein flexibility is similarly described, ff14SB/GLYCAM06j-1 samples a broader GAG conformational space, and dissociation free energy profiles diverge for highly anionic GAGs, but remain comparable for moderately sulfated systems. In addition, we performed molecular dynamics simulations for all systems using the ff14SB/GLYCAM06j-1, CHARMM36m, and ff19SB/GLYCAM06j-1 force fields in a 15 Å solvent box. Structural and energetic analyses revealed no significant impact of the solvent box size on the examined descriptors. These results establish practical benchmarks for accurate atomistic simulations of GAG–protein assemblies and will inform future developments in biomolecular force fields.

## Graphical abstract

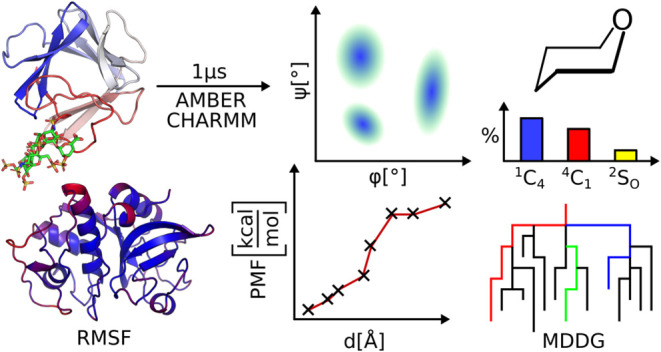

## Introduction

Glycosaminoglycans (GAGs) are linear and predominantly sulfated polysaccharides composed of repeating disaccharide units and are present in all animal cells. Their building blocks consist of alternating amino sugars and glucuronic or iduronic acids, except for keratan sulfate, which lacks uronic acid.^1^ Owing to their structural heterogeneity, GAGs are classified as chondroitin sulfate (CS), dermatan sulfate (DS), keratan sulfate (KS), heparin (HP), heparan sulfate (HS), and hyaluronic acid (HA), the only unsulfated and nonproteoglycan member. These polysaccharides participate in diverse biological processes including neuronal development, cell signaling, growth regulation, adhesion, anticoagulation, and wound repair.^2–4^ Disruption of these processes has been linked to tumor growth, viral invasion, spinal cord injury, and corneal opacity.^4–7^ Understanding the molecular basis of protein–GAG recognition is therefore essential. Known GAG-binding partners include growth factors,^8,9^ cysteine cathepsins,^10,11^ SARS-CoV-2 proteins,^12^ N-deacetylase/N-sulfotransferase,^13^ antithrombin,^14^ bone morphogenetic protein 6,^15^ chemokines,^16^ and collagen.^17^ Among these partners, Fibroblast Growth Factors (FGFs) and cysteine cathepsins are particularly well-characterized models for studying GAG-mediated interactions. FGFs regulate key developmental and homeostatic processes through complex formation with heparin or heparan sulfate proteoglycans and their receptors (FGFRs).^18^ These interactions stabilize FGFs and enhance FGFR activation, often by promoting receptor dimerization.^19–21^ Cysteine cathepsins, in contrast, are lysosomal proteases whose maturation and stability can be modulated by GAG binding.^10,11^

Experimental characterization of protein–GAG complexes at atomic resolution remains challenging. X-ray crystallography requires well-diffracting crystals, which are difficult to obtain, and often fails to capture solution conformations.^22^ NMR spectroscopy, though powerful, is constrained by protein size, oligosaccharide length, concentration, and complex stability.^23^ Further complications arise from GAG structural heterogeneity, variable sulfation patterns, repeating sequence motifs, and large chain flexibility.^24–27^ Computational methods provide an efficient alternative, but must address the intrinsic flexibility of GAGs and their highly charged nature. Both pyranose rings and glycosidic linkages can adopt multiple conformations, influencing binding affinities and conformational sampling.^9,28^ Electrostatic effects dominate protein–GAG interactions and are strongly modulated by solvent and ion-specific contributions.^29,30^ Moreover, GAGs frequently exhibit multipose binding, where distinct poses have similar binding free energies,^31,32^ further complicating accurate modeling. In addition, several studies have reported that classical force fields may systematically overestimate glycan–protein binding affinities due to general limitations in the description of intermolecular interactions and the absence of explicit electronic polarization.^33–35^

Molecular dynamics (MD) simulations offer a popular framework to explore these systems by numerically integrating Newton’s equations of motion for interacting particles.^36^ Interatomic forces and potential energies are here described by empirical force fields, which determine the accuracy of simulated energetics and structures. Among available models, ff14SB/GLYCAM06j-1 ^37^ and CHARMM36m^38^ are the most widely used for protein–GAG systems. The key distinction between these potential energy functions lies in CHARMM’s inclusion of the Urey–Bradley potential, which represents the effective interaction between nonbonded 1,3-atoms.

Both force fields have been applied to study GAG–protein complexes.^39–41^ The ff14SB/GLYCAM06j-1 combination has been used to probe GAG binding to procathepsin S, revealing conformational changes that facilitate activation,^11^ and to investigate heparin interactions with FGFs, showing that chain polarity and length modulate orientation and binding stability.^8^ Phosphorylated GAGs, a novel class of biomacromolecules, have also been characterized using this framework, highlighting their enhanced rigidity and binding strength relative to sulfated analogues.^42^ CHARMM extensions have similarly enabled accurate modeling of sulfated GAGs,^43–46^ including adaptive biasing force simulations of chondroitin sulfate^47^ and analyses of proteoglycan linker flexibility.^48^ While both ff14SB/GLYCAM06j-1 and CHARMM36m have been validated for individual proteins and carbohydrates, systematic benchmarking of protein–GAG interactions remains limited. Here, we compare the structural and energetic properties of protein–GAG complexes for these two force fields. Classical molecular dynamics (MD) and umbrella sampling simulations were employed to evaluate binding energetics and conformational stability, with the results subsequently represented in terms of the underlying energy landscape using the Molecular Dynamics to Disconnectivity Graphs (MDDG) procedure^49^ to produce disconnectivity graphs.^50,51^ Structural accuracy is assessed by conformational analysis.

We also investigated the impact of the solvent box size on the structural and energetic descriptors of protein–GAG interactions. No significant differences were observed between simulations performed with solvent buffers of 6 Å and 15 Å using the ff14SB/GLYCAM06j-1 force field. In addition, we tested the same solvent box sizes using the ff19SB/GLYCAM06j-1 force field. However, in this case the calculations were stable only with the larger 15 Å solvent box. Furthermore, for most of the analyzed descriptors, the ff19SB/GLYCAM06j-1 force field was outperformed by ff14SB/GLYCAM06j-1 in describing protein–GAG interactions. The results highlight force-field–dependent differences in protein–GAG recognition and provide guidance for accurate *in silico* characterization of these biologically important systems.

## Methods

### Structures of Analyzed Protein-Glycosaminoglycan Complexes

In this computational study we analyzed interactions in three protein-GAG systems: FGF-1-HP dp6 (dp stands for degree of polymerization), FGF-2-HP dp6 and CatK–C4-S dp6 (Figure 1). The experimental structures were obtained from the Protein Data Bank (PDB ID: 1BFC for FGF-2-HP dp6,^52^ 2AXM for FGF-1-HP dp6, and 4N8W^53^ for CatK–C4-S dp6 ^54^). For FGF-1-HP dp6 simulations only chain A of FGF-1 was considered in this study, since NMR data revealed a 1:1 complex, which was further shown to be biologically relevant.^55,56^ We also considered structures of apo-proteins (PDB ID: 2K43 for FGF-1, 1BFG for FGF-2,^57^ and 5TUN for CatK^58^) for Root Mean Square Fluctuation profile analyses (see section “Conformational Analysis”).

**Figure 1:**
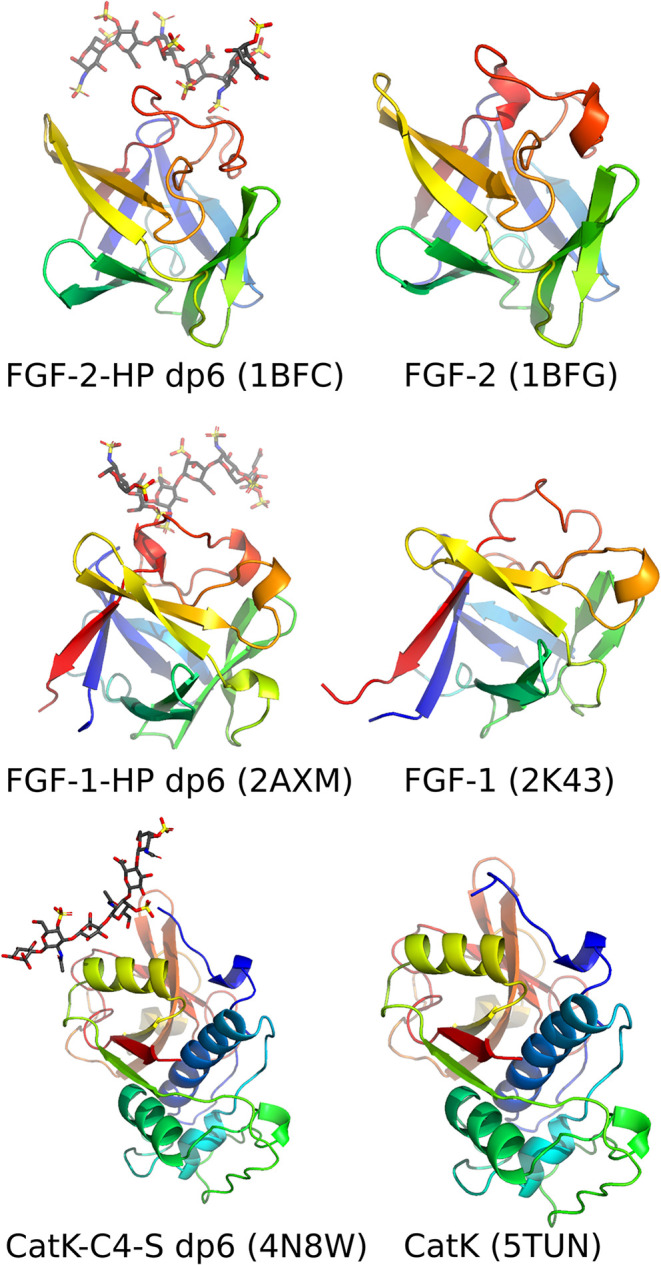
Experimental structures of the protein targets analyzed in the present study in their unbound state and in complex with GAGs. Proteins are shown in rainbow cartoon and GAGs in licorice colored by atom types: C (dark gray), S (yellow), O (red), N (blue). The PDB codes for each structure are provided in brackets.

### All-Atom Molecular Dynamics Simulations in 6 Å Solvent Box

#### ff14SB/GLYCAM06j-1

Three selected complexes, as well as the apo-proteins, were solvated in a TIP3P^59^ octahedral periodic box with a layer of water molecules of 6 Å from the border of the periodic box to the solute and neutralized with counterions (Na^+^). Parameters and charges for sulfated GAGs were taken from the GLYCAM06j-1 force field^60^ and from the literature for sulfate groups.^61^ Energy minimization was carried out in two steps: (i) 0.5 × 10^3^ steepest descent cycles and 10^3^ conjugate gradient cycles with harmonic force restraints of 100 kcal mol^–1^ Å^–2^ on solute atoms and then (ii), 3 × 10^3^ steepest descent cycles and 3 × 10^3^ conjugate gradient cycles without restraints. Then, the system was heated to 300 K for 10 ps with harmonic force restraints of 100 kcal mol^–1^ Å^–2^ on solute atoms and equilibrated for 100 ps at 300 K and 10^5^ Pa in the isothermal isobaric ensemble (NPT). Finally, three 1 μs productive MD runs for each complex was carried out in the NPT ensemble. The SHAKE algorithm, 2 fs time integration step, 8 Å cutoff for nonbonded interactions, and the particle mesh Ewald method^62^ were used. We employed Langevin and Berendsen algorithms for temperature and pressure control, respectively (γ = 1.0 ps^–1^; τ_p_ = 2.0 ps). The structures were saved every 1 ns, which produced 10^3^ in total per simulation used for further analysis. For these simulations the ff14SB and GLYCAM06j-1 force fields were used. We also tested the ff19SB force field^63^ in combination with GLYCAM06j-1 and the OPC explicit water model. However, despite employing two different minimization protocols,^9,64^ this force field combination resulted in artificially high densities, which disrupted the analysis of structural fluctuations.

#### CHARMM36m

Input files for MD simulations in CHARMM36m force field were prepared using charmm-gui.^65^ As for the ff14SB/GLYCAM06j-1 simulations three selected complexes and unbound proteins were solvated in a TIP3P octahedral periodic box with a layer of water molecules of 6 Å from the border of the periodic box to the solute and neutralized with counterions (Na^+^). For each system energy minimization with default charmm-gui options was carried out in the first step. Positional restraints were imposed on backbone atoms, side chains and dihedral angle restraints were used with force constant of 400, 40 kJ/mol^–1^ Å^–2^ and 4 kJ mol^–1^ deg^–2^. A steepest descent algorithm for energy minimization with the tolerance of 10^4^ kJ mol^–1^ nm was employed. Next, an equilibration step was carried out with same positional restraints as in energy minimization. The equilibration simulation was performed in the NVT ensemble for 125 ps at 300 K with temperature coupling using a Nose-Hoover extended ensemble.^66^ Here, covalent bonds involving hydrogen atoms in the solute were constrained using the LINCS algorithm,^67^ while water molecules were treated using the SETTLE algorithm.^68^ Finally, three 1 μs productive MD runs for each complex were carried out in an NTP ensemble. Nose–Hoover^66^ and Parrinello–Rahman^69^ algorithms were used for temperature and pressure control, respectively (τ_t_ = 1.0 ps; τ_p_ = 5.0 ps). A 2 fs integration time step was employed. Nonbonded interactions were calculated using a cutoff of 12 Å. van der Waals interactions were treated using a force-switching scheme with a switching distance of 10 Å and a cutoff of 12 Å. Long-range electrostatics were treated using the particle mesh Ewald method. The structures were saved every 1 ns, which produced 10^3^ in total per simulation for use in subsequent analysis. These simulations were performed using the 2023.2 version of the GROMACS software.^70^

### All-Atom Molecular Dynamics Simulations in 15 Å Solvent Box

We additionally performed simulations of unbound FGF-1, FGF-2, and CatK, as well as their complexes with the respective GAGs present in the experimental structures, using an enlarged solvent box (15 Å) to assess the effect of solvation on the conformational flexibility of both proteins and GAGs. Systems simulated with the ff14SB/GLYCAM06j-1 force field were prepared using the tleap module of AmberTools, whereas those employing CHARMM36m were generated via charmm-gui and converted to an AMBER-compatible format (CHAMBER).

Furthermore, all complexes were also simulated using the ff19SB/GLYCAM06j-1 force field in combination with the OPC solvent model, as recommended by the force field developers. Simulation protocols and parameters for all systems employing the enlarged solvent box (15 Å) and all described force fields were identical to those used in the all-atom simulations performed with ff14SB/GLYCAM06j-1 and a 6 Å solvent box, unless stated otherwise.

### Conformational Analysis

The trajectories obtained with the ff14SB/GLYCAM06j-1 and CHARMM36m approaches were analyzed taking into account conformational aspects of protein-GAG interactions, such as root-mean-square deviation (RMSD) of GAGs in their bound state relative to the initial structure, root-mean-square fluctuations (RMSF) of proteins in unbound and bound states, dihedral angles distributions of glycosidic linkages, sugar ring conformation ratios, and contact maps of protein-GAG residues. RMSD calculations were done for all frames of the MD simulation after aligment to the first reference frame. The RMSD of each GAG was calculated for all heavy atoms. For RMSD calculations, the rmsd module of cpptraj^71^ and the rms module of gmx^70^ were employed for ff14SB/GLYCAM06j-1 and CHARMM36m, respectively. RMSF calculations were performed for all frames of MD simulation and for all atoms. The output values were computed as average (mass-weighted) fluctuations for every residue of the analyzed protein. For ff14SB/GLYCAM06j-1 simulations the atomicfluct module of cpptraj^71^ was employed with the “byres” option, while for CHARMM36m simulations the rmsf module of gmx^70^ was used with the “-res” option. RMSF values obtained from MD simulations were compared to experimentally determined Debye–Waller factors (*B*-factors). They were converted into RMSF by employing the following formula (eq 1) in which *B* is the *B*-factor^72^

1 $${\mathrm{RMSF}}_{\exp}=\sqrt{\frac{3B}{8\pi^{2}}}$$

RMSF_exp_ values were calculated for experimental structures of the protein-GAG complexes (PDB ID: 1BFC, 2AXM, 4N8W). In addition, to assess the potential effect of GAG binding on protein flexibility, we also calculated RMSF_exp_ for apoprotein targets: FGF-1 (PDB ID: 2K43), FGF-2 (PDB ID: 1BFC) and CatK (PDB ID: 5TUN). Since calculated RMSF profiles for each simulation were generally consistent, they were averaged. Experimental fluctuation profiles (based on NMR and X-ray data) were normalized. Due to differences in fluctuation amplitude caused by methodology, normalized RMSF profiles (RMSFN), defined in eq 2, were also considered,

2 $${\mathrm{RMSFN}}_{i}=\frac{{\mathrm{RMSF}}_{i}}{\sqrt{\sum_{i=1}^{n}{\mathrm{RMSF}}_{i}^{2}}}$$

where RMSF_*i*_ is the predicted fluctuation profile for residuals with index *i*, and *n* is the number of residuals. The dihedral angles for glycosidic linkage analysis were defined similarly to the work of Sattelle et al.^28^ as O5_*n*+1_-C1_*n*+1_-O4*_n_*-C4*_n_* and C1_*n*+1_-O4*_n_*-C4*_n_*-C3*_n_* for HP and O5_*n*+1_-C1_*n*+1_-O4*_n_*-C4/3*_n_* and C1_*n*+1_-O4*_n_*-C4/3*_n_*-C3/2*_n_* for C4–S, where *n* stands for the sequential number of a sugar monomeric unit. For this analysis the dihedral module of cpptraj and the TORSION module of the plumed plugin^73^ were employed for ff14SB/GLYCAM06j-1 and CHARMM36m simulations, respectively. These modules were also used to calculate sugar ring conformation ratios. These ratios were calculated by determining in how many frames out of the whole MD simulation a particular conformation occurred. Conformations were defined by two dihedral angles: C1*_n_*-C2*_n_*-C3*_n_*-C4*_n_* and C1*_n_*-O5*_n_*-C5*_n_*-C4*_n_* and the ranges of these dihedral angles for specific sugar ring conformations were defined as in our previous work.^9^ Contact maps were calculated as means of protein-GAG distances over MD simulations. The distance module of cpptraj and the plumed DISTANCE module with centers of mass (COM) defined for each residue were employed for ff14SB/GLYCAM06j-1 and CHARMM36m simulations, respectively.

### Potential of Mean Force

To characterize the energetics of GAG unbinding from the protein targets, we employed the Umbrella Sampling (US) protocol.^74^ The reaction coordinate was defined as the distance between COM of the protein and GAG, with a window spacing of 1.0 Å, and a harmonic force constant of 4 kcal·mol^–1^·Å^–2^. Prior to US simulations, each window was energy-minimized using the ff14SB/GLYCAM06j-1 protocol, independently of the employed force field. During the equilibration of each window, a harmonic restraint was applied to the protein-GAG COM to stabilize the complex at the target distance. The equilibrated coordinates from one window were then used as the starting structure for the subsequent window. Production US simulations were carried out for 100 ns per window. The resulting distance distributions were analyzed using the Weighted Histogram Analysis Method (WHAM),^75,76^ employing a distance range of 20 Å divided into 21 bins and a convergence tolerance of 0.01 kcal·mol^–1^. The final Potential of Mean Force (PMF) profiles were plotted using matplotlib python library.^77^

### Energy Landscape Framework

We visualize the production run trajectories of the complexes in both the ff14SB/GLYCAM06j-1 and CHARMM36m force fields using disconnectivity graphs.^50,51,78,79^ Here we employ a newly presented program, MDDG,^49^ which uses the MD potential energy time series directly. Unlike rigorous energy landscape explorations, this approach avoids geometry optimization. In the energy landscape framework, the nuclear configuration space is explored via discrete path sampling, with minima and transition states located using the OPTIM program.^80^ While this methodology enables sampling across broad regions of chemical space, it is computationally expensive. In contrast, MDDG is a lightweight method designed to visualize the landscape sampled in a single, finite simulation. It is not a replacement for discrete path sampling, but a fast, complementary tool that runs in seconds on a laptop. The program utilizes Savitzky–Golay (SG) smoothing^81,82^ to reveal the conformational basins and the proxy transition states connecting them, actually sampled on the simulation time scale. This treatment directly visualizes the energy landscape sampled in a finite run using a given potential, making it well suited to comparing force fields.

In the MDDG framework, the SG window size and fitted polynomial order control the degree of smoothing via a nominal cutoff frequency. Using the expression derived by Schmid et al.,^83^ we estimate the characteristic cutoff time scale from the approximate −3 dB cutoff frequency *f*_3dB_, obtained via least-squares fitting. With structures saved every ns, adjacent configurations are likely to skip over some intermediate minima. For this reason, the MDDG disconnectivity graph provides a coarse-grained view of the landscape. Even though we do not map and visualize every minimum visited along the trajectory, the global organization of the explored landscape is still partly recovered. This limitation also inflates the apparent barriers, since intervening minima between adjacent MDDG basins are potentially not resolved. For these reasons, we apply gentle smoothing with a five frame sliding window and cubic polynomial fitting. This choice is sufficient to attenuate barrier inflation, while leaving basin assignments essentially unchanged. These settings correspond to an effective cutoff time scale of 3 ns. Initial benchmark results for MDDG with ff14SB/GLYCAM06j-1 and CHARMM36m are presented in the preprint of Neuman et al.^49^

To validate the use of the MDDG program, we performed geometry optimizations of all frames from the ff14SB/GLYCAM06j-1 trajectories used to construct the MDDG disconnectivity graphs. We employed the ff14SB/GLYCAM06j-1 interface implemented in the OPTIM program,^80^ with implicit solvation, and assesed the relaxation in terms of the RMSD upon quenching. Explicit solvent would introduce additional degrees of freedom that render such optimizations impractical on the relevant time scales. Minimization employed the limited-memory BFGS (LBFGS) algorithm^84^ as implemented in OPTIM,^80^ with convergence defined by an RMS gradient threshold of 2.0 × 10^–7^ kcal mol^–1^ Å^–1^. We intended to carry out an analogous analysis for the CHARMM36m force field; however, implicit solvent support has been removed from GROMACS due to insufficient accuracy and poor parallelization. We also explored the use of CHAMBER, but this interface is not yet compatible with OPTIM,^80^ and therefore this benchmark will be performed in the future.

## Results and Discussion

### Glycosaminoglycan Binding Region Analysis

The first thing we investigated in our study was the GAG binding patterns. Here, we analyzed averaged protein-GAG distance maps and GAG RMSD referenced to the initial frame of the MD simulation. Based on the averaged distance maps, GAG binding regions remain comparable between the ff14SB/GLYCAM06j-1 and CHARMM36m force fields for all systems. (Figures S1, S2, and S3). The analysis of RMSD profiles for FGF-2-HP dp6 revealed that in CHARMM36m simulations more conformational space was explored, since in ff14SB/GLYCAM06j-1 the RMSD remained at a level close to the initial structure (<5 Å) (Figures S4 and S7), while in CHARMM36m it reached 14 Å in all three runs. Structural analysis shows that the increase in RMSD in ff14SB/GLYCAM06j-1 corresponds to a change of HP orientation on the protein surface, while in CHARMM36m it additionally involves a subtle change of binding region (bending of carbohydrate chain). The opposite trend was observed for FGF-1-HP dp6 (Figures S5 and S8). Here, based on the RMSD profiles, for the CHARMM36m force field HP remained close to its initial structure, exhibiting an increase of RMSD to ∼6 Å, while in ff14SB/GLYCAM06j-1 several clusters of structures were identified in the range 2 Å to 15 Å. These findings are in agreement with our previously obtained data for 10 μs MD simulations, in which the HP RMSD stabilized after 6 μs.^9^ Structural analysis also shows that for CHARMM36m, the binding region observed in the initial structure was preserved with bending of carbohydrate chain observed, and for ff14SB/GLYCAM06j-1 additionally a change of GAG orientation occurs. For the CatK–C4-S dp6 complex comparable RMSD profiles were obtained for the ff14SB/GLYCAM06j-1 and CHARMM36m force fields (Figures S6 and S9). Structural analysis shows that for CHARMM36m, the increase in RMSD corresponds to a conformational change of a GAG within the same binding region, while for ff14SB/GLYCAM06j-1, partial dissociation of the carbohydrate chain combined with movement of C4–S on the protein surface was observed. The results suggest that GAG exploration of the protein surface might correspond to overall protein charge. For system with strong electrostatic interactions (FGF-2–HP dp6, protein charge: +11), CHARMM36m explores different binding regions, which corresponds to higher RMSD values with respect to the first frame. CatK is intermediate in between FGF-1 and FGF-2 (protein charge: +7) and comparable results were obtained. For FGF-1 (protein charge: +2), higher conformational flexibility was observed in ff14SB/GLYCAM06j-1. This trend is an interesting initial indication that CHARMM36m may explore more conformational space in terms of GAG sampling for systems with higher charge, whereas ff14SB/GLYCAM06j-1 does so for systems with lower charges. However, the current three example studies are likely insufficient to support a definitive conclusion, and this question will be examined further in future studies.

Analysis of the average distance maps for the ff14SB/GLYCAM06j-1, CHARMM36m, and ff19SB/GLYCAM06j-1 force fields with a 15 Å solvent box size indicates that the binding regions are comparable across all three force fields (Figure S10). Furthermore, in comparison with simulations performed using a 6 Å solvent box, the binding regions remain conserved.

RMSD profiles for HP in complex with FGF-2 obtained from simulations with a 15 Å solvent box reveal trends similar to those observed for the 6 Å solvent box (Figure S11). In the case of ff14SB/GLYCAM06j-1, RMSD remained below 5 Å in two out of three simulations. In contrast, for CHARMM36m, RMSD reached 15 Å in all three simulations. The RMSD profiles for ff19SB/GLYCAM06j-1 were comparable to those obtained with ff14SB/GLYCAM06j-1. Similarly, in two out of three MD simulations, RMSD remained below 5 Å for the majority of the simulation time.

For the FGF-1–HP dp6 system simulated in a 15 Å solvent box, higher RMSD values were observed for ff14SB/GLYCAM06j-1 than for CHARMM36m, consistent with the results obtained using the 6 Å solvent box (Figure S12). In one ff14SB/GLYCAM06j-1 simulation, RMSD increased to 15 Å. In contrast, in CHARMM36m simulations, RMSD values reached 8–10 Å in all cases. The smallest structural deviations were observed for ff19SB/GLYCAM06j-1, where RMSD remained around 5 Å for most of the simulation time.

For the CatK–C4-S dp6 complex simulated in a 15 Å solvent box, RMSD values increased during the initial phase of the MD simulations for all three force fields, reaching values in the range of 10–20 Å (Figure S13). A similar trend was observed in simulations with the 6 Å solvent box. In ff14SB/GLYCAM06j-1 simulations, once C4–S dp6 adopted a given conformation, only minor structural changes were observed, as reflected by relatively stable RMSD values. In CHARMM36m simulations, the GAG sampled a broader range of conformations, which is supported by the larger RMSD fluctuations. For ff19SB/GLYCAM06j-1, C4–S dp6 remained close to a single conformation after the initial RMSD increase in one simulation, whereas in the other two simulations multiple states were sampled, similarly to the behavior observed in CHARMM36m. Overall, these results suggest that although increasing the solvent box size introduced some differences, the majority of the trends observed for simulations performed with the 6 Å solvent box were preserved.

### Effect of GAG Binding on Amino Acid Residues Fluctuations

In this study, we evaluated the ability of the ff14SB/GLYCAM06j-1 and CHARMM36m force fields to reproduce protein flexibility in the presence and absence of GAGs. Simulated RMSF profiles (Figures S14–S16) were compared with normalized experimental data, and the agreement with experiment was quantified using Pearson (*r*_*p*_) and Spearman (*r*_*s*_) correlation coefficients (Table 1). On average, ff14SB/GLYCAM06j-1 exhibits higher *r*_*p*_ and *r*_*s*_ values. The statistical significance of the differences between the two force fields was assessed using paired Student’s *t* tests and Wilcoxon signed-rank tests. For *r*_*p*_, the resulting *p*-values were 0.2850 and 0.5625, respectively, indicating no statistically significant difference. Similarly, for *r*_*s*_, the *p*-values of 0.2803 and 0.4375, respectively, indicate that the two force fields perform comparably within statistical uncertainty.

**Table 1:** Pearson (*r*_*p*_) and Spearman (*r*_*s*_) Correlation Coefficients between Predicted and Experimental RMSF Profiles for FGF-1, FGF-2, and CatK in Unbound State and in Complex with Respective Protein Targets

	FF14SB bound		CHARMM36m bound		FF14SB unbound		CHARMM36m unbound	
Protein	*r*_*p*_	*r*_*s*_	*r*_*p*_	*r*_*s*_	*r*_*p*_	*r*_*s*_	*r*_*p*_	*r*_*s*_
FGF-2	0.7308	0.7457	0.4840	0.5105	0.7247	0.7385	0.2559	0.3231
FGF-1	0.6109	0.5325	0.5716	0.5172	0.5346	0.5269	0.5945	0.6219
CatK	0.6243	0.6901	0.5748	0.6296	0.5547	0.6461	0.6655	0.7017

We then examined the influence of GAG binding on protein flexibility by comparing RMSF profiles between unbound and bound states (Table S1). Both *r*_*p*_ and *r*_*s*_ values indicate strong correlations for all systems (>0.9), suggesting that GAG binding exerts only a modest effect on global protein fluctuations. A noticeable deviation was observed only for FGF-2 for CHARMM36m, where only moderate correlations was observed (*r*_*p*_ = 0.63 and *r*_*s*_ = 0.76). We also observed that, for this system, the RMSD values with respect to the first frame were significantly higher for CHARMM36m than for ff14SB/GLYCAM06j-1 (Figure 7). For ff14SB/GLYCAM06j-1, the fluctuations were largely confined to the GAG around a single binding site, whereas in CHARMM36m the GAG explored additional binding sites during the simulation. This behavior is also confirmed in the clear subfunnel visible in the CHARMM36m disconnectivity graph (Figure 7). As mentioned before, FGF-2 is the system with the highest charge, which may be linked to its distinct binding behavior and the greater structural flexibility observed between these force fields. Consequently, when RMSF profiles for the bound and unbound states are highly correlated, the corresponding RMSF difference profiles should be interpreted with caution. In such cases, the true signal is small relative to numerical noise, which can obscure meaningful comparison with experimental difference profiles.

This limitation is reflected in the correlation coefficients obtained for ΔRMSF between computational and experimental data (Table S2). For most systems, no clear correlation was observed, as the computed differences are often comparable to simulation noise. These results indicate that when ΔRMSF is marginal, predicting the effect of GAG binding on protein flexibility becomes substantially more challenging.

Overall, these results demonstrate that both ff14SB/GLYCAM06j-1 and CHARMM36m provide a consistent description of protein residue fluctuations, and that GAG binding minimally perturbs global protein dynamics. The analysis also underscores a limitation of RMSF-based metrics: small, localized changes in dynamics may be difficult to resolve against the inherent variability of molecular simulations.

We also analyzed RMSFN profiles for FGF-2, FGF-1, and CatK in both bound and unbound states, using a 15 Å solvent box (Figures S17–S19). A comparison of Pearson’s and Spearman’s correlation coefficients obtained for ff14SB/GLYCAM06j-1 and CHARMM36m simulations with 6 Å and 15 Å solvent boxes revealed that in 8 out of 12 cases the 6 Å solvent box provided more accurate results, showing better agreement with experimental data than the 15 Å box (Tables 1 and 2). These findings suggest that the 6 Å solvent box may capture structural fluctuation features more faithfully, likely due to packing conditions that more closely resemble the experimental environment.

**Table 2:** Pearson (*r*_*p*_) and Spearman (*r*_*s*_) Correlation Coefficients between Predicted and Experimental RMSF Profiles for FGF1, FGF2, and CatK in Unbound State and in Complex with Respective Protein Targets for Simulations with 15 Å Solvent Box

	FF14SB bound		CHARMM36m bound		FF19SB bound	
Protein	*r*_*p*_	*r*_*s*_	*r*_*p*_	*r*_*s*_	*r*_*p*_	*r*_*s*_
FGF2	0.7083	0.6998	0.4079	0.5410	0.6921	0.6636
FGF1	0.5933	0.5582	0.5312	0.6031	0.5419	0.5019
CatK	0.5514	0.6241	0.4424	0.5899	0.5338	0.6482

	FF14SB unbound		CHARMM36m unbound		FF19SB unbound	
Protein	*r*_*p*_	*r*_*s*_	*r*_*p*_	*r*_*s*_	*r*_*p*_	*r*_*s*_
FGF2	0.7222	0.7559	0.4996	0.5527	0.7367	0.7676
FGF1	0.5303	0.4884	0.5666	0.5168	0.6049	0.5619
CatK	0.6199	0.6592	0.4702	0.5341	0.6414	0.6682

Analysis of the ff19SB/GLYCAM06j-1 results showed that, for unbound proteins, this force field provided the most accurate description of RMSFN profiles in nearly all cases, outperforming both ff14SB/GLYCAM06j-1 and CHARMM36m for both 6 Å and 15 Å solvent boxes. The only exception was CatK, for which CHARMM36m with a 6 Å solvent box exhibited better correlation with experimental data. These observations suggest that ff19SB may be the most suitable choice for studying fluctuations in unbound proteins.

Similar challenges in predicting the effect of GAG binding on protein fluctuations are also observed in simulations performed with a 15 Å solvent box. In nearly all cases, the Pearson and Spearman correlations between RMSFN profiles in the bound and unbound states are higher for computationally derived data than for experimentally derived values (Table S3). Consequently, no clear correlation is observed between computational and experimental ΔRMSF profiles, as the computed differences are often comparable to the intrinsic noise of the simulations (Table S4). These results further indicate that when ΔRMSFN values are small, accurately predicting the impact of GAG binding on protein flexibility becomes substantially more challenging.

In contrast, the presence of GAG reduced the accuracy of ff19SB/GLYCAM06j-1. In all cases, both Pearson’s and Spearman’s correlation coefficients were lower than those obtained for ff14SB/GLYCAM06j-1 with the same solvent box size. This result suggests that for investigating protein fluctuations in protein–GAG systems, ff14SB/GLYCAM06j-1 combined with a 6 Å solvent box may represent the optimal setup.

### Analysis of Monosaccharide Ring Puckering

We analyzed the distribution of monosaccharide ring conformations, known as ring puckering, based on the trajectories obtained using the ff14SB/GLYCAM06j-1 and CHARMM36m force fields (Figure 2). The puckering parameters were defined according to the method of Cremer and Pople.^85^ This analysis provides insight into the preferred conformations and flexibility of GAG monosaccharides in different force fields, which is essential for understanding their structural and functional roles.

**Figure 2:**
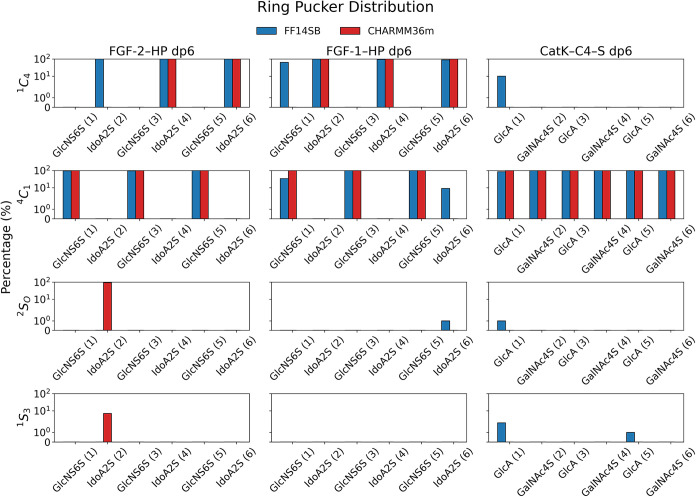
Ring puckering conformations of HP and C4–S residues observed in the MD simulation in ff14SB/GLYCAM06j-1 and CHARMM36m force fields. The residue numbering (in parentheses) is from the reducing to the nonreducing end.

For the GlcNS­(6S) and GalNAc­(4S) residues, the dominant is ^4^ *C*_1_ chair conformation of the sugar ring in both force fields. A notable discrepancy occurs for the first monosaccharide unit of HP in complex with FGF-1, where in ff14SB/GLYCAM06j-1 this residue additionally samples the ^1^ *C*_4_ conformation. It is important to note that this increased flexibility appears at one end of the HP chain, where weaker protein–carbohydrate interactions provide less stabilization of the ring geometry, enabling conformational transitions. Experimental studies indicate that GlcNS­(6S) preferentially adopts the ^4^ *C*_1_ conformation,^86^ and a similar preference is reported for GalNAc­(4S), although minor populations of alternative conformations have also been observed. Altogether, these results suggest that both force fields reproduce the conformational preferences of these residues with comparable accuracy.

GlcA predominantly adopts the ^4^ *C*_1_ conformation in both force fields. In CHARMM36m, only this chair form is observed, whereas ff14SB/GLYCAM06j-1 samples minor populations of additional, mostly skewed conformations. Moreover, the terminal GlcA residue exhibits increased flexibility, with a substantial population of the ^1^ *C*_4_ conformation observed in ff14SB/GLYCAM06j-1. Again, weaker interactions with the protein environment appear to facilitate such transitions. Experimental evidence supports ^4^ *C*_1_ as the predominant GlcA conformation.^87^ Since the deviations between the force fields are minor, both appear to capture the conformational landscape of GlcA consistently.

Analysis of ring puckering for IdoA shows that in both force fields the dominant conformation is ^2^ *S*_*O*_, although the differences between the two potentials are more pronounced compared to other residues. In CHARMM36m, 2-IdoA­(2S) samples two skew conformations, whereas in AMBER the preferred form is ^1^ *C*_4_. Additionally, the 6-IdoA­(2S) residue occasionally adopts ^4^ *C*_1_ and ^2^ *S*_*O*_ in ff14SB/GLYCAM06j-1, while these forms are not populated in CHARMM36m. Experimental studies indicate that IdoA exists in an equilibrium between ^1^ *C*_4_ and ^2^ *S*_*O*_, with additional chair and skewed conformations also observed in smaller amounts.^86^ Hence, both force fields capture the conformational behavior of IdoA reasonably well. The discrepancies may arise from limited sampling, as our previous 10 μs ff14SB/GLYCAM06j-1 simulations^9^ visited all of the mentioned conformations, suggesting that longer trajectories may be required to fully observe the IdoA equilibrium.

Overall, the results show that both force fields provide a broadly consistent description of ring puckering in GAG monosaccharide units. These observations support the robustness of the structural features observed across simulations and suggest that both force fields are suitable for characterizing GAG conformational ensembles at the level required for our analysis.

The solvent box size has no significant impact on the conformational preferences of monosaccharide units constituting GAG chains. The observed differences are quantitative rather than qualitative. For ff14SB/GLYCAM06j-1, the most populated conformation of GalNAc­(4S) is ^4^ *C*_1_. Similarly, for GlcA, ^4^ *C*_1_ remains the dominant conformation, followed by minor populations of ^1^ *C*_4_ and ^1^ *S*_3_ (Figure S20). For the CHARMM36m force field, regardless of the box size, both GalNAc­(4S) and GlcA strongly prefer the ^4^ *C*_1_ conformation. Results obtained with ff19SB/GLYCAM06j-1 show a comparable distribution of GalNAc­(4S) and GlcA conformations to ff14SB/GLYCAM06j-1.

For the GlcNS­(6S) residue in ff14SB/GLYCAM06j-1, the most preferred conformation is ^4^ *C*_1_. The solvent box size has only a minor effect on the population of the alternative ^1^ *C*_4_ conformation. These differences are primarily observed for terminal carbohydrate residues, which, due to weaker interactions with the protein, exhibit increased conformational flexibility. In the case of CHARMM36m, there is a clear and consistent preference for the ^4^ *C*_1_ conformation of GlcNS­(6S), independent of the solvent box size. Results for ff19SB/GLYCAM06j-1 remain comparable to ff14SB/GLYCAM06j-1, with ^4^ *C*_1_ being the dominant conformation and only minor contributions from ^1^ *C*_4_.

Within the ff14SB/GLYCAM06j-1 force field, the preferred conformation for the IdoA­(2S) residue is ^1^ *C*_4_, although small variations in the population of skew-boat conformations are observed. In the 15 Å solvent box, traces of ^2^ *S*_*O*_ are present. For CHARMM36m, similar overall trends are observed for IdoA­(2S). In both 6 Å and 15 Å solvent boxes, the first IdoA­(2S) residue in the FGF-2–HP dp6 system shows a clear preference for ^2^ *S*_*O*_ and ^1^ *S*_3_ conformations, whereas the remaining IdoA­(2S) residues predominantly adopt the ^1^ *C*_4_ conformation. In the case of ff19SB/GLYCAM06j-1, similarly to ff14SB/GLYCAM06j-1, ^1^ *C*_4_ is the preferred conformation for IdoA­(2S), with only minor populations of ^2^ *S*_*O*_ detected.

Overall, these results indicate that increasing the solvent box size from 6 Å to 15 Å does not alter the qualitative conformational landscape of GAG monosaccharides, but may slightly modulate the relative populations of minor ring puckering states. Importantly, the differences observed between force field families are more pronounced than those associated with the solvent box size, suggesting that the choice of force field has a greater impact on ring conformational equilibria than the extension explicit solvent layer.

### Glycosidic Linkages Conformational Analysis

We additionally examined the conformational landscape of HP and C4–S glycosidic linkages to characterize the intrinsic preferences of the carbohydrate backbone. For the GlcNS­(6S)–IdoA­(2S) linkage in ff14SB/GLYCAM06j-1, two dominant conformations were observed: ϕ = −80°, ψ = 110° and ϕ = −80°, ψ = −60° (Figure 3A). The latter state was populated exclusively by the first glycosidic linkage (Figure S21). In contrast, IdoA­(2S)–GlcNS­(6S) consistently adopted a single conformation (ϕ = 110°, ψ = 100°) across all linkages of this type. The presence of these states agrees with earlier reports by Sattelle et al.,^28^ our own microsecond-scale simulations,^9^ as well as studies on phosphorylated GAGs,^42^ indicating that microsecond time scales may be required to sample the full conformational repertoire. In CHARMM36m, however, GlcNS­(6S)–IdoA­(2S) populated only the (ϕ = −90°, ψ = 110°) state, irrespective of its position within the chain. Likewise, IdoA­(2S)–GlcNS­(6S) exhibited a single preferred conformation (ϕ = 120°, ψ = 110°), with narrower distributions than for ff14SB/GLYCAM06j-1. These observations are consistent with previous 5 μs CHARMM36m simulations employing different solvent models,^41^ and suggest that CHARMM36m may impose higher rotational barriers for these linkages, thus requiring longer simulations or enhanced-sampling approaches to achieve comparable state coverage.

**Figure 3:**
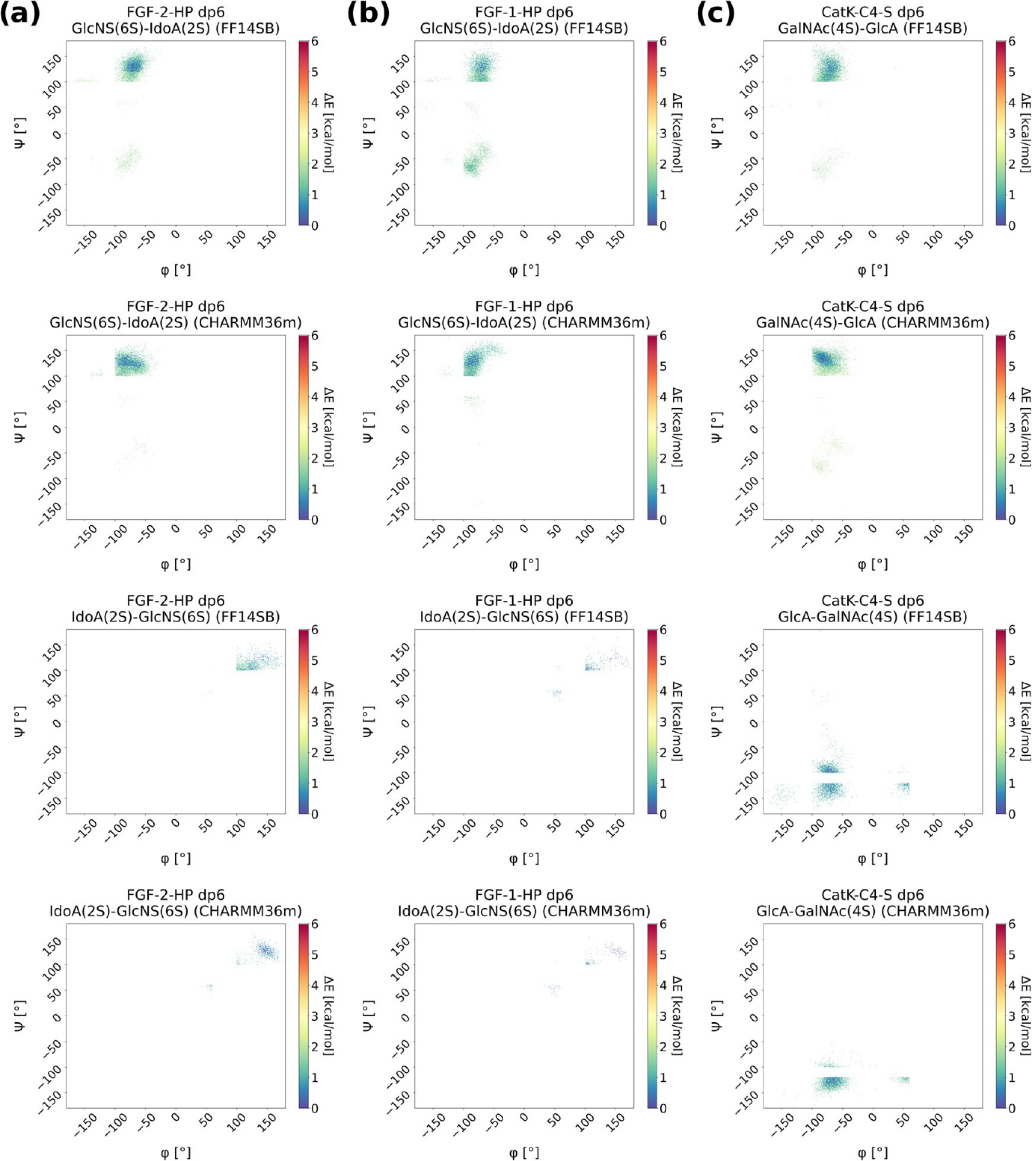
Heatmaps for the glycosidic linkage showing the ϕ and ψ dihedral angles for (a) FGF-2-HP dp6, (b) FGF-1-HP dp6 and (c) CatK–C4-S dp6 using the AMBER and CHARMM force fields.

For HP in complex with FGF-1, the glycosidic distributions in ff14SB/GLYCAM06j-1 remained broadly consistent with those from the FGF-2–HP dp6 system; however, the second conformation (ϕ = −80°, ψ = −60°) was sampled by all GlcNS­(6S)–IdoA­(2S) linkages (Figure 3B). A third state (ϕ = −90°, ψ = 50°) appeared for the 3GlcNS­(6S)–4IdoA­(2S) linkage (Figure S22). For IdoA­(2S)–GlcNS­(6S), the preferred conformation remained (ϕ = 110°, ψ = 110°), although the distribution was somewhat narrower than in the FGF-2 complex. Overall, the conformational states are consistent with those previously reported for HP in complex with FGF-2 and with earlier experimental and computational studies.^9,28,42^ When compared to FGF-2–HP dp6, these results suggest that protein electrostatics may modulate glycosidic flexibility, particularly in ff14SB/GLYCAM06j-1. This trend was also observed in CHARMM36m, albeit to a much lesser extent. In CHARMM36m, while GlcNS­(6S)–IdoA­(2S) predominantly occupied the (ϕ = −90°, ψ = 110°) state, some linkages transiently sampled (ϕ = −90°, ψ = 50°). IdoA­(2S)–GlcNS­(6S) retained the same preferred conformation as in ff14SB/GLYCAM06j-1, but with even narrower distributions. These data reinforce the notion that CHARMM36m imposes higher torsional barriers, consistent with our observations for the FGF-2–HP complex and previous CHARMM-based studies.^41^

For the GalNAc­(4S)–GlcA and GlcA–GalNAc­(4S) linkages in the CatK–C4–S complex, both force fields yielded broadly similar conformational distributions (Figure 3C). In GalNAc­(4S)–GlcA, the dominant state was (ϕ = −90°, ψ = 110°) in both force fields, with a secondary state at (ϕ = −90°, ψ = −90°) appearing at several positions along the chain (Figure S23). CHARMM36m additionally sampled a third state (ϕ = −60°, ψ = −30°) for the 1GalNAc­(4S)–2GlcA linkage. For the GlcA–GalNAc­(4S) linkage, ff14SB/GLYCAM06j-1 populated two states, (ϕ = −80°, ψ = −120°) and (ϕ = −80°, ψ = −90°), with similar probability, whereas CHARMM36m favored the former configuration. Qualitative inspection of the heatmaps further indicates that ff14SB/GLYCAM06j-1 samples a broader region of the conformational space.

In summary, while both force fields reproduce similar qualitative preferences for glycosidic linkages, ff14SB/GLYCAM06j-1 consistently samples a wider range of conformational states. This enhanced flexibility may reflect lower torsional barriers relative to CHARMM36m. Moreover, our results suggest that protein electrostatics can modulate glycosidic flexibility, with a stronger effect observed in ff14SB/GLYCAM06j-1 than in CHARMM36m.

Comparison of the glycosidic linkage maps obtained from simulations performed with 6 Å and 15 Å solvent boxes revealed only minor differences. For the ff14SB/GLYCAM06j-1 force field, regardless of the box size, the FGF-2–HP dp6 system exhibits a dominant GlcNS­(6S)–IdoA­(2S) conformation at ϕ = −80°, ψ = 110° (Figure S24A). Additionally, in the 6 Å solvent box, a second local minimum is observed at ϕ = −80°, ψ = −60°. The IdoA­(2S)–GlcNS­(6S) linkage consistently adopts a single conformation at ϕ = 110°, ψ = 100° for both solvent box sizes.

For the FGF-1–HP dp6 system, two minima are present for the GlcNS­(6S)–IdoA­(2S) linkage, irrespective of the box size: one at ϕ = −80°, ψ = 110° and another at ϕ = −80°, ψ = −60° (Figure S24B). The IdoA­(2S)–GlcNS­(6S) linkage displays a minimum at ϕ = 110°, ψ = 100° in both solvent environments. A comparable distribution is observed for the CatK–C4–S dp6 system at both solvent box sizes (Figure S24C). For the GalNAc­(4S)–GlcA linkage, the most populated conformations occur at ϕ = −90°, ψ = 110° and ϕ = −90°, ψ = −90°, whereas for GlcA–GalNAc­(4S), the dominant states are located at ϕ = −80°, ψ = −120° and ϕ = −80°, ψ = −90°. In the 15 Å solvent box, these two minima merge into a single broader minimum centered at ϕ = −80°, ψ = −120°.

More pronounced differences between the 6 Å and 15 Å solvent boxes are observed for the CHARMM36m force field. For the GlcNS­(6S)–IdoA­(2S) linkage in both FGF systems simulated with the 6 Å solvent box, the dominant conformation is located at ϕ = −80°, ψ = 110° (Figure S25AB). In simulations employing the 15 Å solvent box, a second state previously observed only for ff14SB/GLYCAM06j-1 at ϕ = −80°, ψ = −60° becomes populated in the FGF-1–HP dp6 system and appears sporadically in the FGF-2–HP dp6 complex. The minima for the IdoA­(2S)–GlcNS­(6S) linkage remain generally consistent between solvent box sizes, centered at ϕ = 110°, ψ = 100°.

For the GalNAc­(4S)–GlcA linkage, conformational preferences are largely comparable between box sizes in CHARMM36m simulations, although the second conformation at ϕ = −90°, ψ = −90° is sampled more frequently in the 15 Å solvent box (Figure S25C). For the GlcA–GalNAc­(4S) linkage, a trend similar to that observed for ff14SB/GLYCAM06j-1 is detected: the two minima identified for the 6 Å solvent box at ϕ = −80°, ψ = −120° and ϕ = −80°, ψ = −90° merge into a single minimum at ϕ = −80°, ψ = −120° in the larger solvent environment.

Results obtained for the ff19SB/GLYCAM06j-1 force field show very similar conformational preferences of glycosidic linkages to those observed for ff14SB/GLYCAM06j-1 in the 15 Å solvent box (Figure S26AB). The most noticeable differences are found for the IdoA­(2S)–GlcNS­(6S) linkage in the FGF-1–HP dp6 system and for the GlcA–GalNAc­(4S) linkage in CatK–C4–S dp6 (Figure S26C). In both cases, the conformational space sampled with ff19SB/GLYCAM06j-1 is slightly broader, indicating a modest increase in linkage flexibility relative to ff14SB/GLYCAM06j-1.

Overall, increasing the solvent box size has a more pronounced effect on the spectrum of sampled glycosidic conformations in CHARMM36m simulations than in ff14SB/GLYCAM06j-1. Nevertheless, the observed differences are predominantly quantitative rather than qualitative, as no new dominant conformational states emerge upon box expansion. This indicates that the overall conformational landscape of glycosidic linkages remains robust with respect to solvent box size, while the choice of force field exerts a comparatively greater influence on the extent of conformational sampling.

### Energetic Description of GAG Unbinding

To assess the energetics underlying protein–GAG recognition, we performed umbrella-sampling simulations followed by PMF reconstruction along the COM–COM separation coordinate (Figure 4). While the use of a COM distance as a reaction coordinate may lead to hidden minima in the PMF profile for long GAG chains, previous studies have shown that the GAG fragment corresponding to the crystallographically resolved binding pose can dissociate and reassociate along this coordinate while recovering its experimental conformation.^88^ Therefore, we employed the COM–COM distance as the reaction coordinate in our force-field benchmarking. Overall, the two force fields produced qualitatively similar dissociation profiles, yet systematic quantitative differences were observed, particularly for highly sulfated heparin systems.

**Figure 4:**
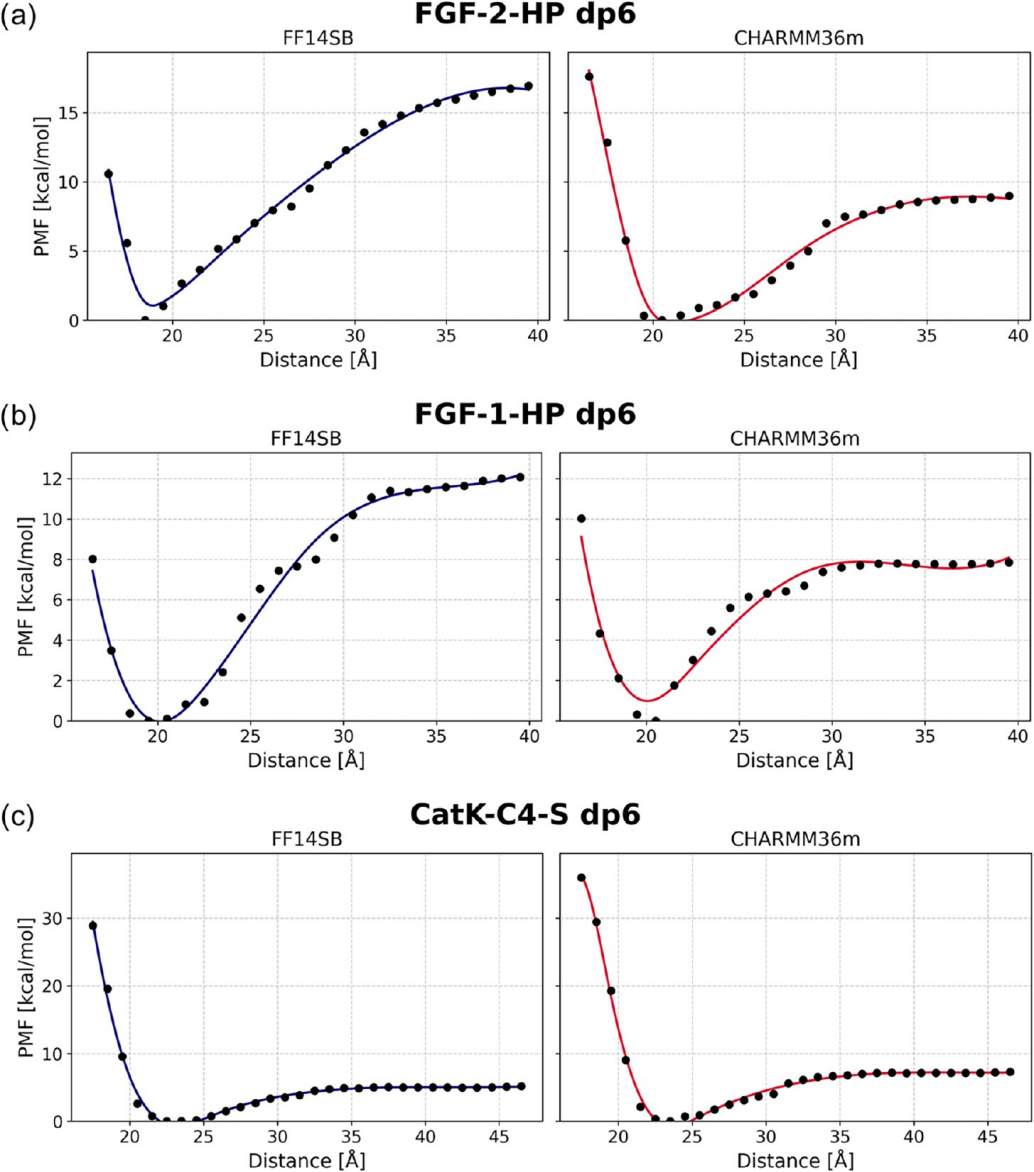
PMF dissociation profiles for HP dp6 in complex with FGF-2 (a) and FGF-1 (b), and for C4–S dp6 in complex with CatK (c), computed using the AMBER and CHARMM force fields.

For the FGF systems, ff14SB/GLYCAM06j-1 consistently yielded higher dissociation free energies than CHARMM36m, indicating more persistent electrostatic stabilization. For the FGF-2–HP dp6 complex, the required dissociation free energy reached 17.0 kcal mol^–1^ in ff14SB/GLYCAM06j-1 versus 9.0 kcal mol^–1^ in CHARMM36m. A similar pattern was observed for FGF-1–HP dp6, where the energies required for GAG dissociation were 12.1 kcal mol^–1^ (ff14SB/GLYCAM06j-1) and 7.9 kcal mol^–1^ (CHARMM36m), consistent with the weaker binding of FGF-1 relative to the more positively charged FGF-2. In contrast, the CatK–C4-S dp6 system exhibited more stable binding in simulations performed with the CHARMM36m force field. In this case, the dissociation energy was 7.3 kcal mol^–1^, compared to simulations using ff14SB/GLYCAM06j-1, where the energy difference between the bound and unbound states was lower (5.2 kcal mol^–1^).

We compared our computational PMF profiles with the available experimental binding data for the FGF-2-HP, FGF-1-HP, and CatK–C4-S complexes. For FGF-2-HP (*K*_D_ = 39 nM; Δ*G*° ≈ −10.2 kcal mol^–1^),^89^ the CHARMM-derived binding free energy was in closer agreement with experiment. A similar trend was observed for the FGF-1-HP complex (*K*_D_ = 1100 nM; Δ*G*° ≈ −8.1 kcal mol^–1^),^90^ for which the experimental study also employed a hexameric HP ligand, consistent with our computational model. In contrast, both ff14SB/GLYCAM06j-1 and CHARMM36m underestimated the binding strength for the CatK–C4–S system (*K*_D_ = 10 nM; Δ*G*° ≈ −10.9 kcal mol^–1^).^91^ This discrepancy may arise from the fact that the experimental measurements were performed using a C4–S oligomer closer in size to an octamer rather than a hexamer, which could enhance the effective binding affinity. Nevertheless, the CHARMM36m force field produced a binding free energy that was closer to the experimental value than that obtained with ff14SB/GLYCAM06j-1.

Overall, these results show that ff14SB/GLYCAM06j-1 systematically predicts stronger binding than CHARMM36m for highly sulfated HP, whereas for moderately sulfated GAGs such as C4–S the two force fields yield nearly identical energetics. Comparison with experimental affinities further suggests that ff14SB/GLYCAM06j-1 may overestimate interaction strengths for strongly anionic GAGs. Both PMF profiles consistently identify FGF-2 as the stronger binder relative to FGF-1, reflecting intrinsic differences in their electrostatic surfaces. Notably, binding free energies obtained with the CHARMM36m force field are overall closer to the available experimental estimates, suggesting that this force field may provide a somewhat more reliable description of protein–GAG interactions in PMF-based binding studies, although this observation is based on a limited number of systems. It should also be noted that discrepancies between computational and experimental binding energies may arise from factors such as multivalency effects and concentration conditions in experimental measurements. Therefore, these results should be interpreted with caution.

## Visualizing the Trajectories with Disconnectivity Graphs

Figure 5 shows that the structural differences between the MDDG proxy minima (raw MD) and the corresponding optimized (quenched) structures are small, on the order of ∼2 Å RMSD. These small RMSD changes indicate that minimization does not change the structures significantly. The ff14SB/GLYCAM06j-1 comparison therefore supports treating the raw MD proxy minima as representative of the corresponding local minima for the purpose of comparing the potential energy landscapes. Consistent with this conclusion, the mean RMSD upon minimization is modest across the three systems, 1.59 Å for 1BFC, 1.72 Å for 2AXM, and 1.65 Å for 4N8W, suggesting that the proxy minima already lie close in configuration space to their associated local minima. As a result, they are adequate for characterizing the qualitative organization of the sampled landscape.

**Figure 5:**
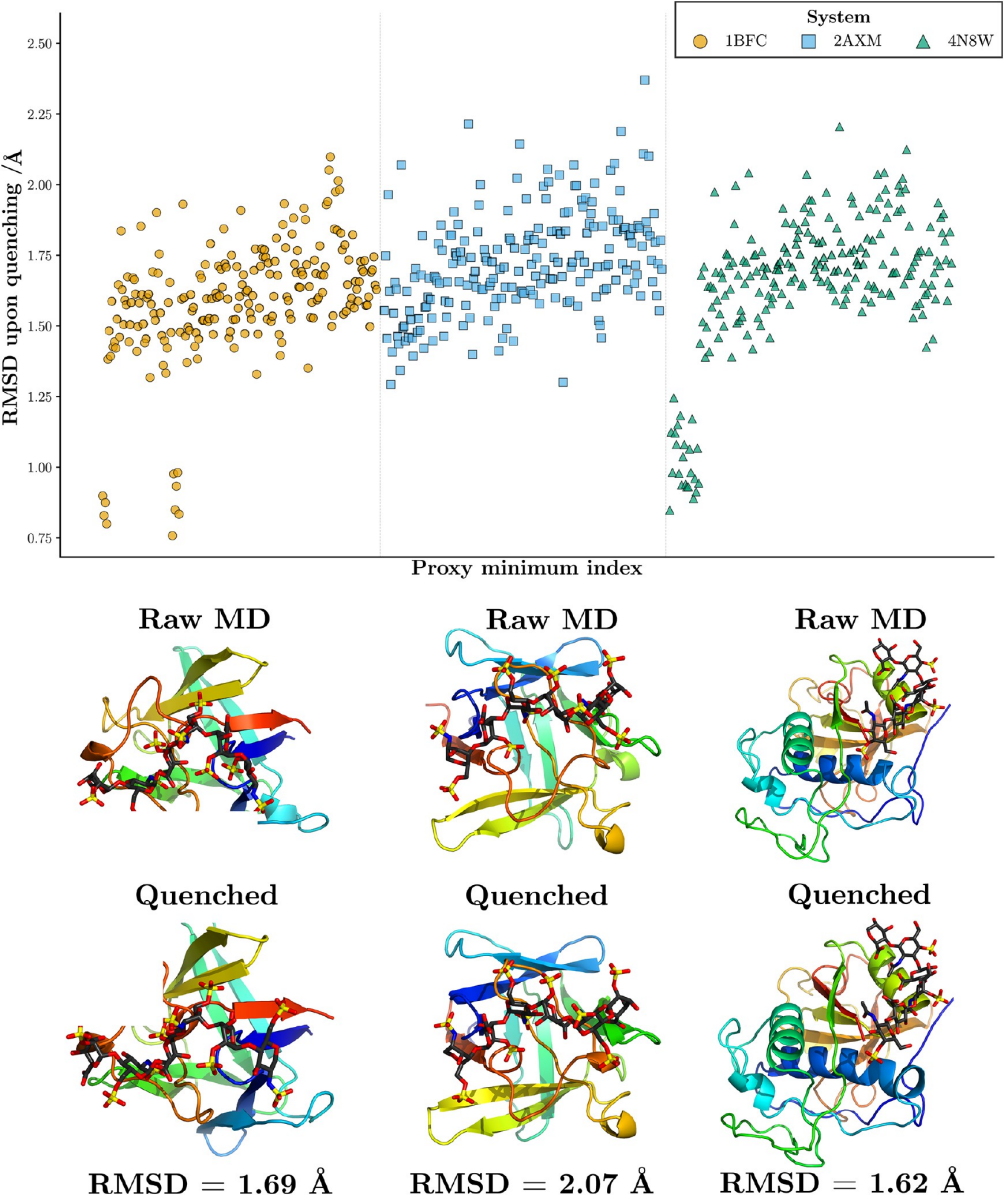
Plot of the RMSD following geometry optimization for the simulations of the three systems used to construct the ff14SB/GLYCAM06j-1 MDDG disconnectivity graphs, shown as a function of the proxy minimum index along the MD trajectory identified by MDDG (top); and the asterisked (*) ff14SB/GLYCAM06j-1 structures from Figures 6, 8, and 10 with their respective quenched structures and RMSD values (bottom).

For the FGF-2-HP dp6 complex disconnectivity graphs (Figures 6 and 7), the proxy barriers appear similar for both force fields, with those obtained using CHARMM being slightly higher. In terms of structural diversity, CHARMM explores minima with larger RMSD values relative to the first frame. The separation into subfunnels leading to distinct structures is, however, more ordered, with clearly demarcated subfunnels when GAG RMSD is used as the order parameter. Interestingly, the CHARMM simulation revisits structures featuring different binding sites, whereas the ff14SB/GLYCAM06j-1 trajectories predominantly sample smaller structural variations within the same binding region, resulting in a more consistent RMSD profile.

**Figure 6:**
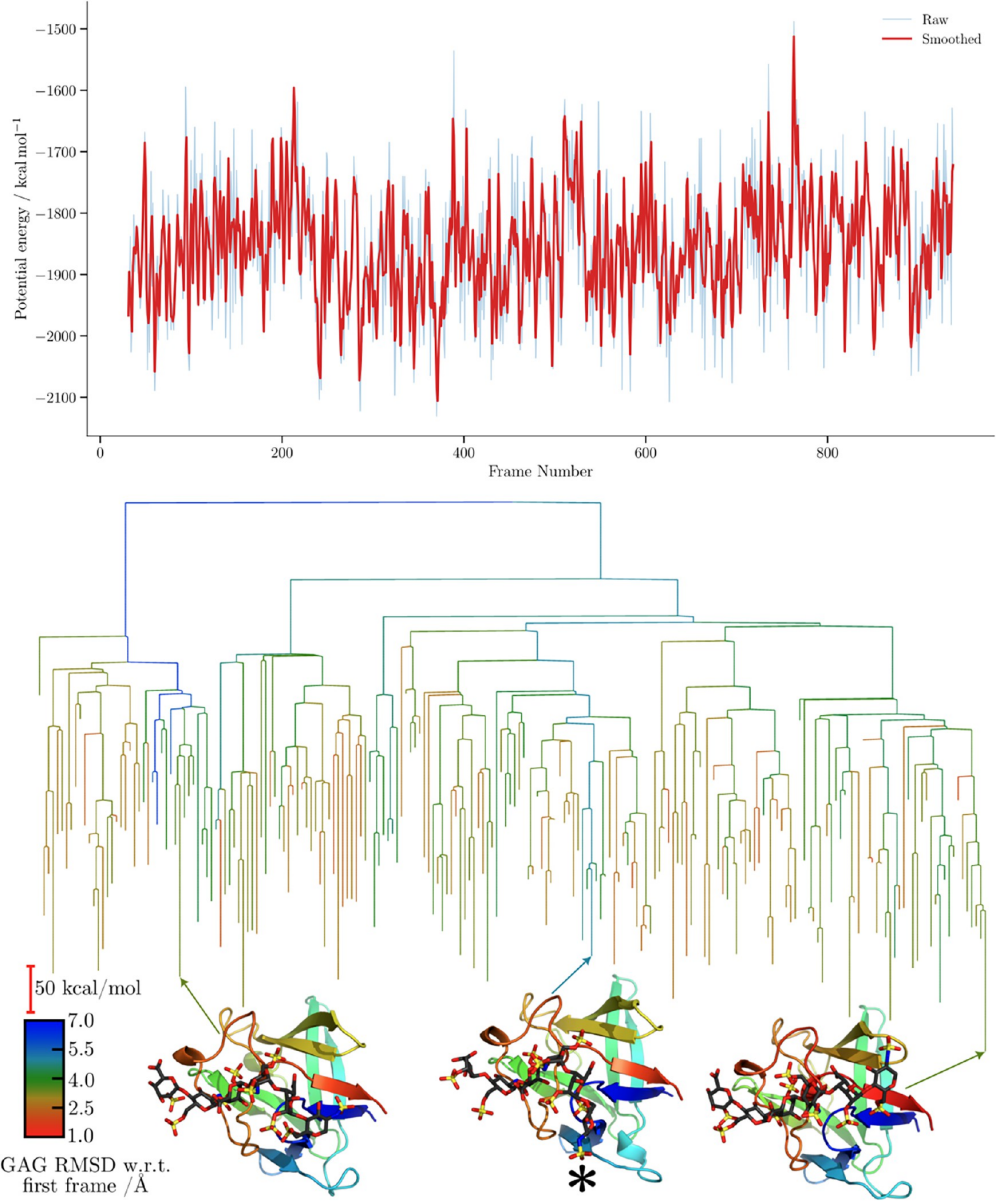
Potential energy time series and the corresponding MDDG disconnectivity graphs for the ff14SB/GLYCAM06j-1 simulation of the 1BFC complex. The branches of each disconnectivity graph are colored by the GAG RMSD relative to the first frame of the corresponding simulation. Representative structures from the main conformational funnels of each tree are shown, with arrows indicating the corresponding proxy minima. Structure marked with an asterisk (*) corresponds to Figure 5.

**Figure 7:**
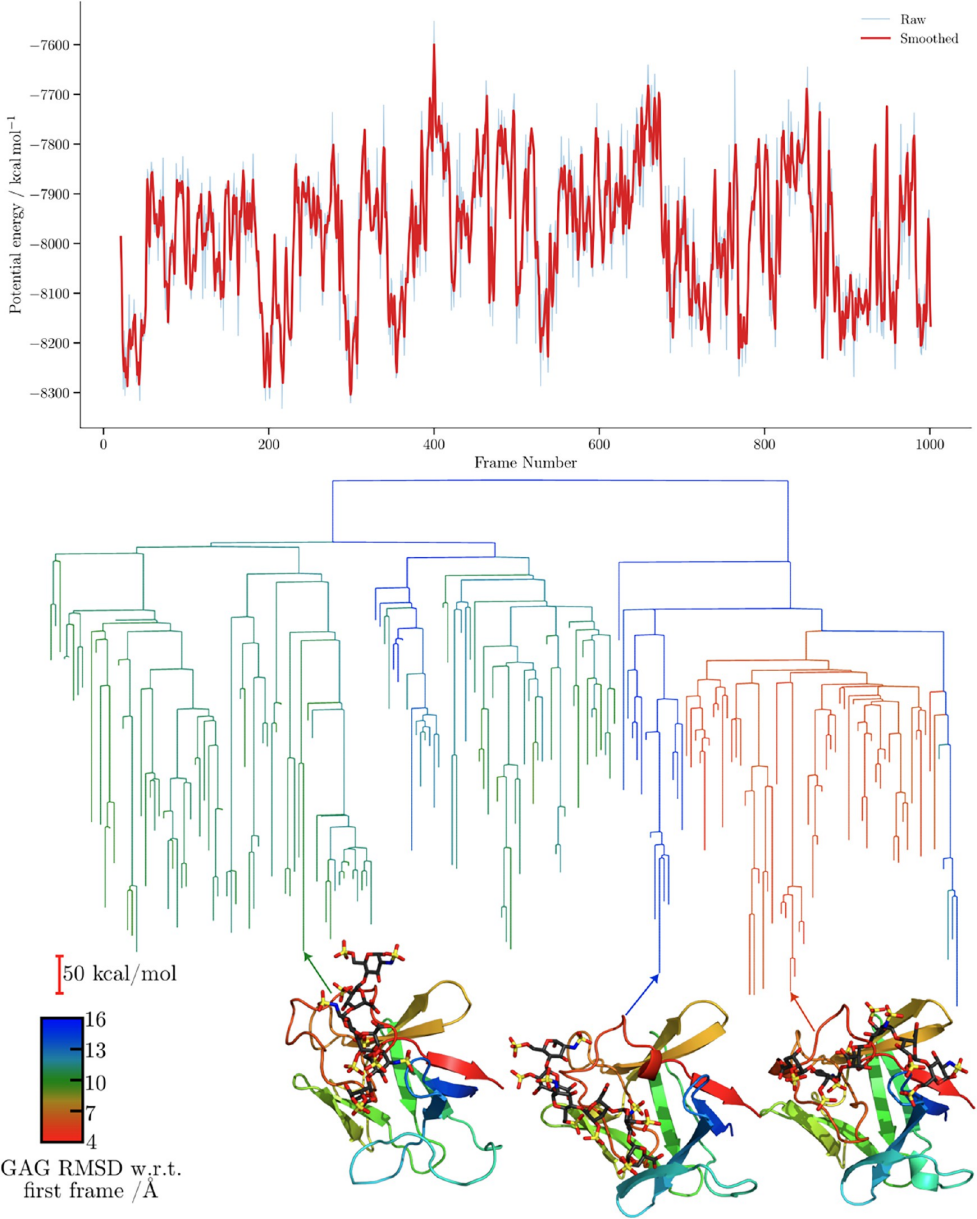
Potential energy time series and the corresponding MDDG disconnectivity graphs for the CHARMM simulation of the 1BFC complex. The branches of each disconnectivity graph are colored by the GAG RMSD relative to the first frame of the corresponding simulation. Representative structures from the main conformational funnels of each tree are shown, with arrows indicating the corresponding proxy minima.

For the FGF-1-HP dp6 complex disconnectivity graphs (Figures 8 and 9), the proxy barriers are markedly higher for CHARMM. In terms of structural diversity, both force fields explore minima with similar RMSD values relative to the first frame. The separation into subfunnels leading to distinct structures appears largely uncorrelated for both force fields when RMSD is employed as the order parameter. In both cases, the trajectories predominantly sample conformations within the same binding region, resulting in a comparatively consistent RMSD profile. Consequently, the observed RMSD differences arise primarily from fluctuations of the GAGs within this region rather than from transitions to alternative binding modes, and, therefore, no clear separation into subfunnels is observed in the disconnectivity graphs.

**Figure 8:**
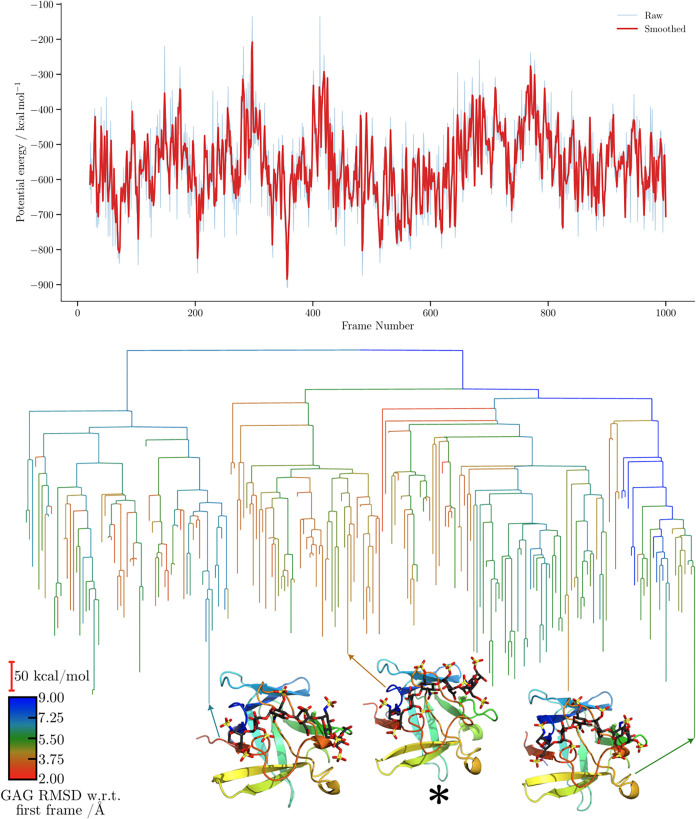
Potential energy time series and the corresponding MDDG disconnectivity graphs for the ff14SB/GLYCAM06j-1 simulation of the 2AXM complex. The branches of each disconnectivity graph are colored by the GAG RMSD relative to the first frame of the corresponding simulation. Representative structures from the main conformational funnels of each tree are shown, with arrows indicating the corresponding proxy minima. Structure marked with an asterisk (*) corresponds to Figure 5.

**Figure 9:**
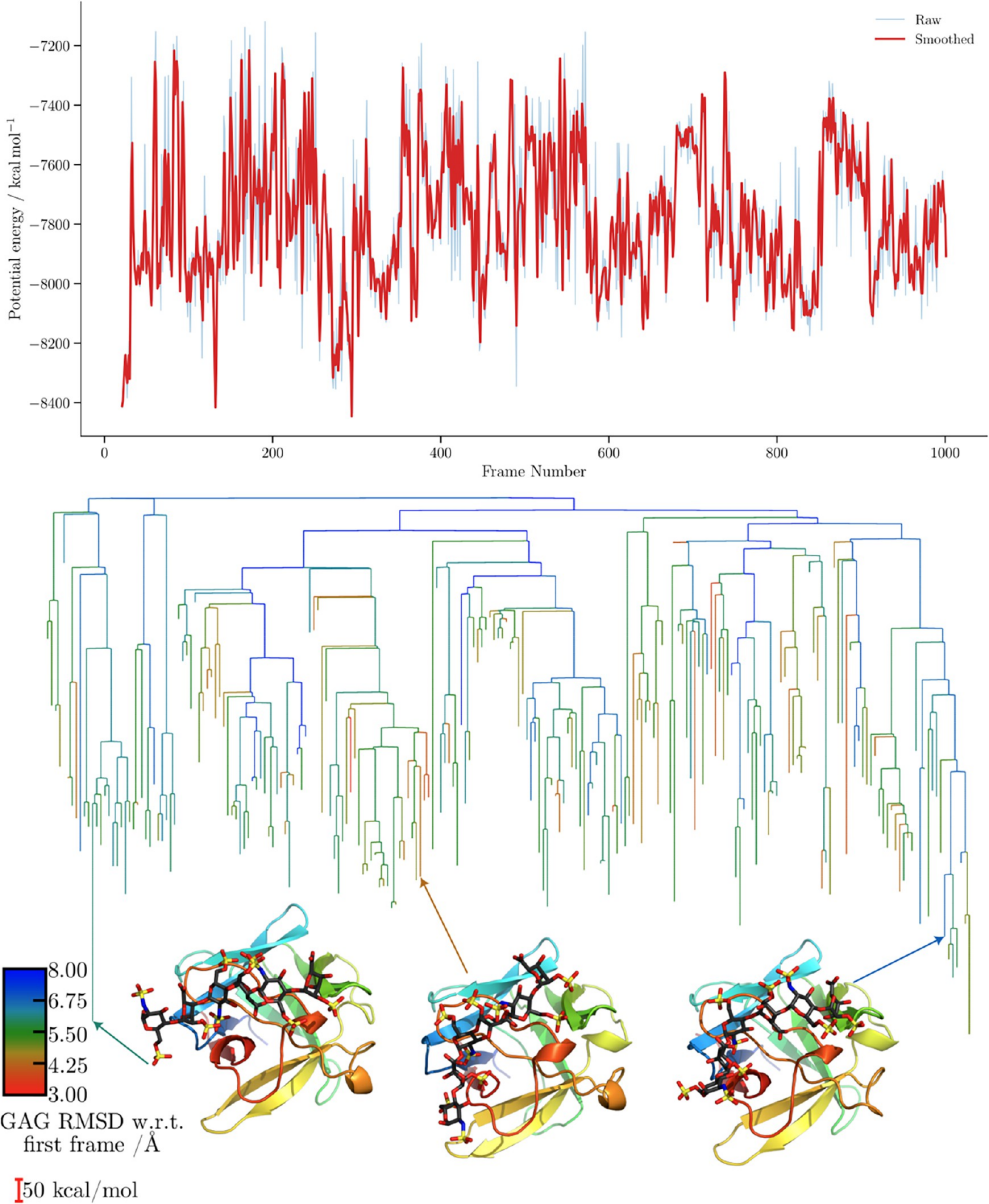
Potential energy time series and the corresponding MDDG disconnectivity graphs for the CHARMM simulation of the 2AXM complex. The branches of each disconnectivity graph are colored by the GAG RMSD relative to the first frame of the corresponding simulation. Representative structures from the main conformational funnels of each tree are shown, with arrows indicating the corresponding proxy minima.

For the CatK–C4-S dp6 complex disconnectivity graphs (Figures 10 and 11), the proxy barriers are again markedly higher for CHARMM. In terms of structural diversity, both force fields explore minima exhibiting substantial variation, with consistently high RMSD values relative to the first frame. The separation into subfunnels leading to distinct structures appears correlated for both force fields when RMSD is employed as the order parameter. In both cases, the trajectories predominantly sample conformations within the same binding region; however, this region appears highly flexible, as the GAG conformation changes significantly throughout the simulation, resulting in persistently large RMSD values with respect to the initial structure. Consequently, the observed RMSD differences arise primarily from fluctuations of the GAGs within this region rather than from transitions to alternative binding modes.

**Figure 10:**
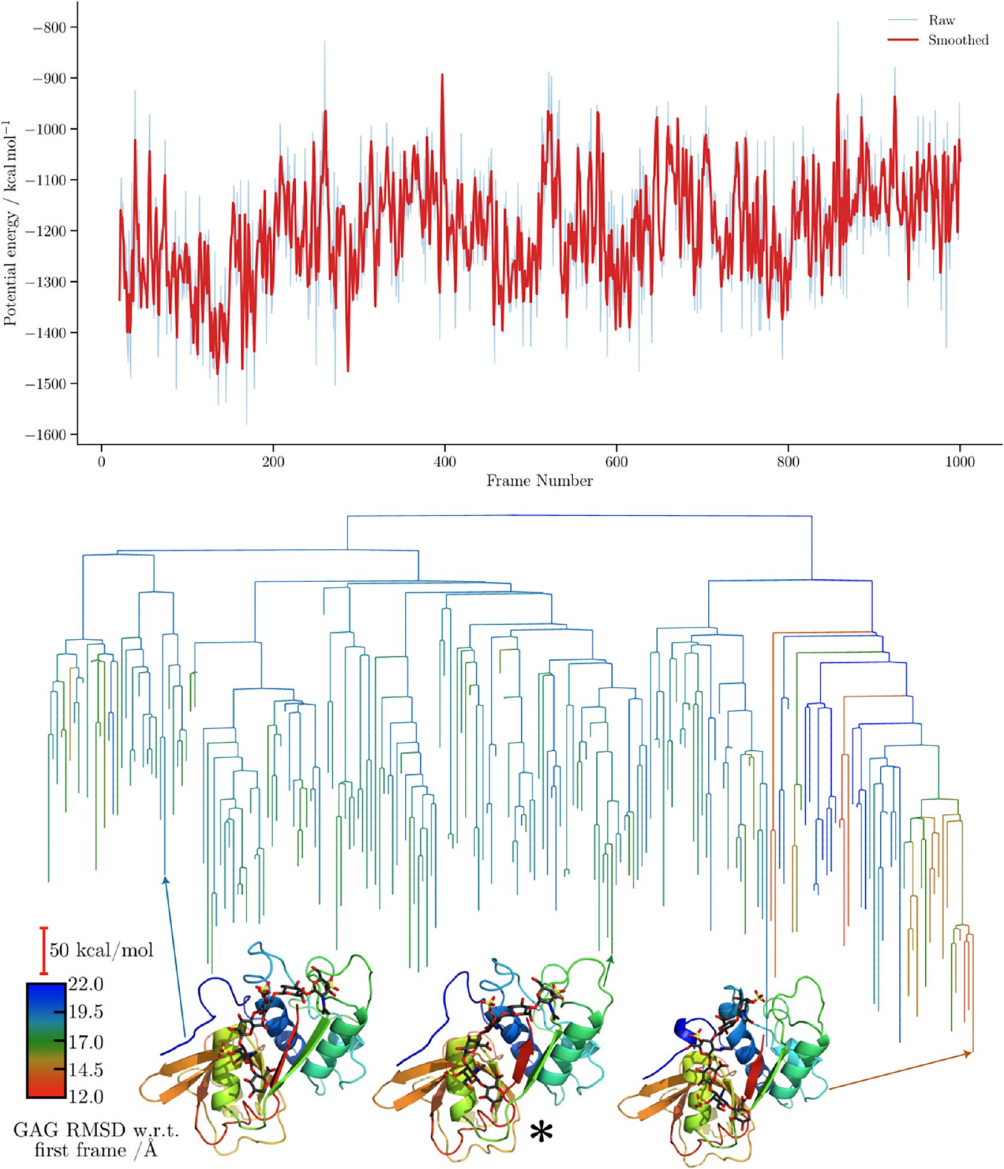
Potential energy time series and the corresponding MDDG disconnectivity graphs for the ff14SB/GLYCAM06j-1 simulation of the 4N8W complex. The branches of each disconnectivity graph are colored by the GAG RMSD relative to the first frame of the corresponding simulation. Representative structures from the main conformational funnels of each tree are shown, with arrows indicating the corresponding proxy minima. Structure marked with an asterisk (*) corresponds to Figure 5.

**Figure 11:**
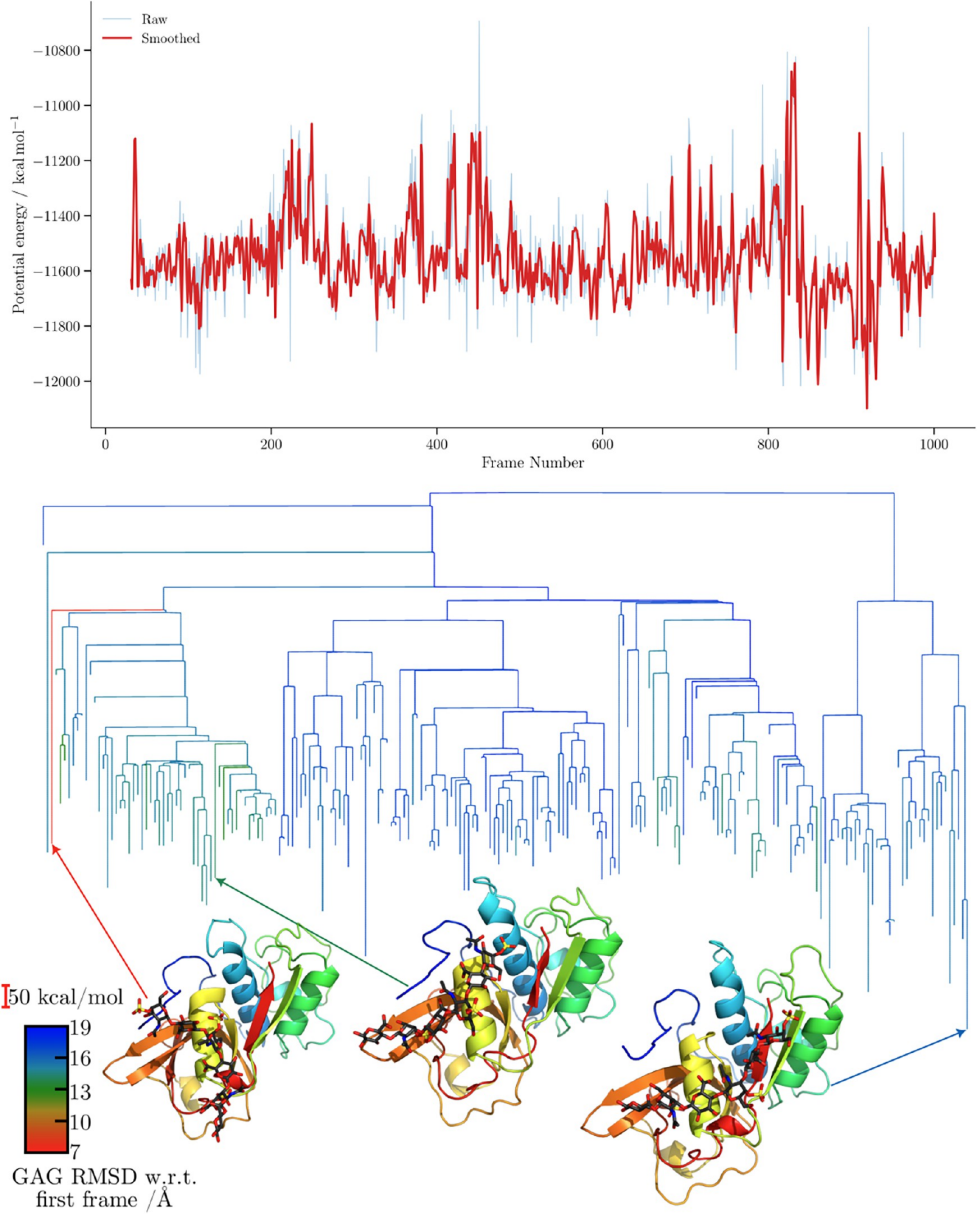
Potential energy time series and the corresponding MDDG disconnectivity graphs for the CHARMM simulation of the 4N8W complex. The branches of each disconnectivity graph are colored by the GAG RMSD relative to the first frame of the corresponding simulation. Representative structures from the main conformational funnels of each tree are shown, with arrows indicating the corresponding proxy minima.

Overall, CHARMM and ff14SB/GLYCAM06j-1 exhibit clearly different energy fluctuation profiles, which is reflected in the topology of the underlying energy landscape and their disconnectivity graphs. CHARMM appears to show somewhat higher proxy barriers. The simulations with both potentials explore roughly the same number of MDDG proxy minima across all systems (approximately 200). In the explored region of the CHARMM–FGF-2-HP dp6 landscape, the GAG RMSD appears to be highly relevant for the organization of the landscape. This structure is reflected in the strong correlation between the funnel/subfunnel topology and this order parameter. Simulations with increased box size or the ff19SB/GLYCAM06j-1 potential did not exhibit significantly different MDDG disconnectivity graphs (Figures S27–S35). We found weak evidence that with increased box sixe, the organization into subfunnels is more pronounced with stronger correlations with GAG RMSD. Due to the subtle differences, it is difficult to tell whether this effect is simply due to different sampling in the simulations or if it reflects something about the organization of the potential energy surface. To resolve this question, geometry optimization methods and extensive landscape searching would be required. Nevertheless, MDDG demonstrates that the GAG RMSD is a clear organizing factor in the potential energy landscapes traversed in the simulations. For completeness, the disconnectivity graphs relating to the 15 Å box and ff19SB/GLYCAM06j-1 simulations are included in the Supporting Information.

## Conclusions

The present study aimed to compare the predictive capabilities of ff14SB/GLYCAM06j-1 and CHARMM36m force fields in describing protein–GAG interactions. Analysis of GAG dynamics on protein surfaces suggests a potential relationship between overall protein charge and GAG mobility. For highly positively charged proteins, GAGs tended to explore a larger fraction of the protein surface for CHARMM36m, whereas for proteins with lower positive charge, GAG mobility was enhanced in ff14SB/GLYCAM06j-1.

Examination of RMSF profiles indicates that both force fields provide a comparable description of protein flexibility, regardless of the presence or absence of bound GAGs. It is important to note, however, that the application of RMSF-based metrics to capture the effects of GAG binding is inherently limited, particularly for systems in which RMSF differences are marginal. Although the smaller box yielded higher Pearson and Spearman correlation coefficients, artificial self-interactions may still occur; therefore, such simulations must be interpreted with caution.

Regarding ring puckering, both force fields consistently reproduced the preferred conformations of GAG monosaccharides. Similarly, glycosidic linkage distributions revealed comparable dominant conformations in ff14SB/GLYCAM06j-1 and CHARMM36m. Nevertheless, ff14SB/GLYCAM06j-1 consistently sampled a broader conformational space, suggesting that energy barriers for glycosidic rotations are generally higher in CHARMM36m, and longer simulation times or enhanced sampling may be required to achieve similar coverage.

Energetic analysis of GAG dissociation reveals that, for highly anionic GAGs, ff14SB/GLYCAM06j-1 predicts systematically stronger binding than CHARMM, leading to an overestimation relative to experimental data, whereas for moderately sulfated GAGs, both force fields yield comparable PMF profiles. Analysis of MDDG proxy minima and their subsequent quenching indicates that the identified proxy minima closely approximate the true local minima in configuration space, with RMSD changes upon minimization generally below 2 Å across all systems. This result justifies the use of MDDG for mapping the energy landscapes of protein–GAG complexes and the comparison of landscape organization between ff14SB/GLYCAM06j-1 and CHARMM36m.

Disconnectivity graph analysis further reveals that CHARMM36m tends to exhibit higher proxy barriers and more clearly defined subfunnels for certain systems (e.g., 1BFC), whereas ff14SB/GLYCAM06j-1 more frequently samples structural variations within the same binding region. These differences highlight the complementary nature of the two force fields: CHARMM36m provides a more rigid view of funnel organization and barrier heights, while ff14SB/GLYCAM06j-1 captures a broader exploration of local minima and GAG conformational diversity.

We also compared different solvation box sizes. For the ff19SB/GLYCAM06j-1 force field with the OPC water model, a 6 Å buffer produced artificially high densities, preventing reliable analysis of structural fluctuations. Increasing the buffer to 15 Å resolved this issue; however, the newer AMBER force field still underperformed relative to the well-established ff14SB/GLYCAM06j-1 with the TIP3P water model. In contrast, no significant differences between 6 Å and 15 Å buffers were observed for ff14SB/GLYCAM06j-1 or CHARMM36m in terms of GAG RMSD, ring pucker and glycosidic linkages. In the RMSF analysis, ff19SB/GLYCAM06j-1 provided the most accurate profiles for unbound proteins compared with experimental data. This force field was outperformed by ff14SB/GLYCAM06j-1 for protein–GAG complexes, and therefore we do not recommend its use for such systems.

Overall, these results indicate that both ff14SB/GLYCAM06j-1 and CHARMM36m capture structural, dynamical, and energetic aspects of protein–GAG interactions at a useful level. The combined insights from RMSD analyses, structural diversity, and disconnectivity graphs provide a comprehensive framework for interpreting protein–GAG interactions and can guide force field selection based on the balance between conformational sampling and energetic resolution. Specifically, ff14SB/GLYCAM06j-1 may be preferable for studies requiring extensive sampling of GAG conformational space, while CHARMM36m could be advantageous when focusing on precise energetic quantification or systems with highly charged proteins, where stronger intramolecular barriers slow down the sampling.

## Supplementary Material



## Data Availability

The data supporting this study are openly available in the Zenodo repository (10.5281/zenodo.19049876). The repository contains MD trajectories of FGF-2-HP dp6, FGF-1-HP dp6, and CatK–C4-S dp6 complexes in ff14SB/GLYCAM06j-1 and CHARMM36m force fields, as well as topologies and initial coordinates. MDDG is open source code available at https://github.com/vilmosneuman/MDDG.

## Notes

Averaged distance maps for protein–GAG complexes from molecular dynamics simulations; GAG RMSD profiles relative to initial structures; representative protein–GAG structures, including initial configurations and structures corresponding to maximum RMSD values; normalized RMSF profiles for apo and complex protein states; ring puckering distributions from simulations in a 15 Å solvent box; glycosidic linkage heatmaps from 15 Å solvent box simulations; glycosidic linkage heatmaps for selected linkages from 6 Å solvent box simulations; disconnectivity graphs from 15 Å solvent box simulations; Pearson and Spearman correlation coefficient tables between RMSF profiles (PDF)
