# Supplementary material for: Comparison of Protein–Glycosaminoglycan Interactions in ff14sb/GLYCAM06j‑1 and CHARMM36m Force Fields
Source: J Chem Inf Model. 2026 May 4;66(10):6159–80. doi: 10.1021/acs.jcim.5c03159 (PMC13213835; doi:10.1021/acs.jcim.5c03159)
Supplement: Supplementary file 1 [file ci5c03159_si_001.pdf]

Supporting Information

Comparison of protein-glycosaminoglycan interactions in ff14sb/GLYCAM06j and CHARMM36m force fields

Krzysztof K. Bojarski[*,1,2,3], Patryk A. Wesołowski[*,2], Diksha Dewan[†,2], Łukasz J. Dziadek[†,4], Vilmos Neuman[†,5], Bernard R. Brooks[6], Jacek Czub[1], Martin Zacharias[3,7], Adam K. Sieradzan[4], and David J. Wales[*,2]

[1]Department of Physical Chemistry, Gdansk University of Technology, Narutowicza 11/12, Gdansk, Poland
[2]Yusuf Hamied Department of Chemistry, University of Cambridge, Lensfield Road, Cambridge, CB2 1EW, U.K.
[3]Center for Functional Protein Assemblies, Technical University of Munich, Ernst-Otto-Fischer-Straße 8, Garching, Germany
[4]Department of Theoretical Chemistry, University of Gdansk, Wita Stwosza 63, Gdansk, Poland
[5]Department of Chemistry, Physical and Theoretical Chemistry Laboratory, University of Oxford, South Parks Road, Oxford OX1 3QZ, U.K.
[6]Laboratory of Computational Biology, National Heart Lung and Blood Institute, National Institutes of Health, Bethesda, Maryland 20892, United States
[7]Physics Department, Technical University of Munich, James-Franck Strasse 1, Garching, Germany

[*]Corresponding authors: krzysztof.bojarski@pg.edu.pl, paw61@cam.ac.uk, dw34@cam.ac.uk
[†]These authors contributed equally.

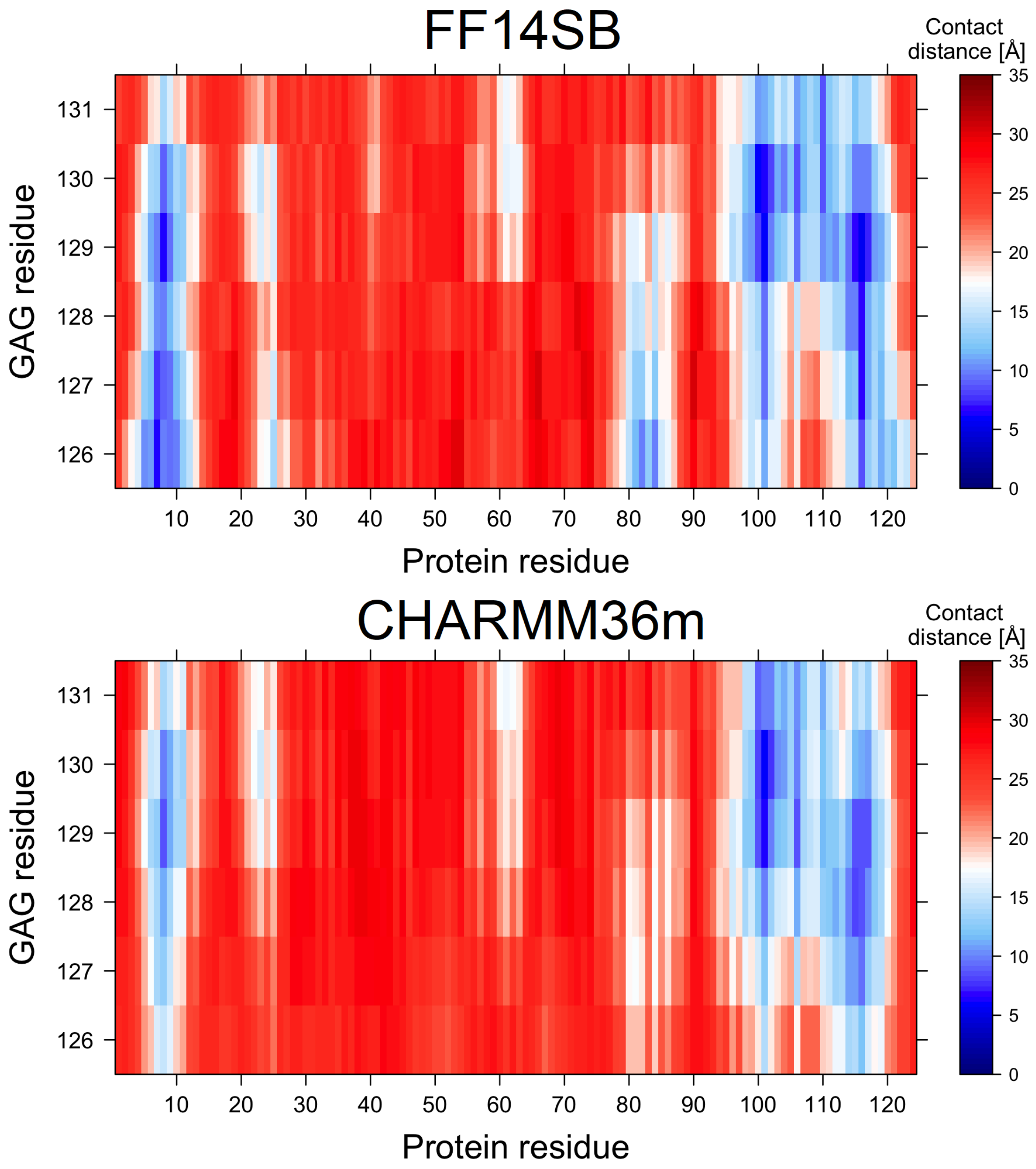

Figure S1: Distance contact maps for the FGF-2-HP dp6 complex (PDB ID: 1BFC) with the ff14SB/GLYCAM06j-1 and CHARMM36m force fields. Values on the contact maps are calculated as mean distances from whole MD simulation.

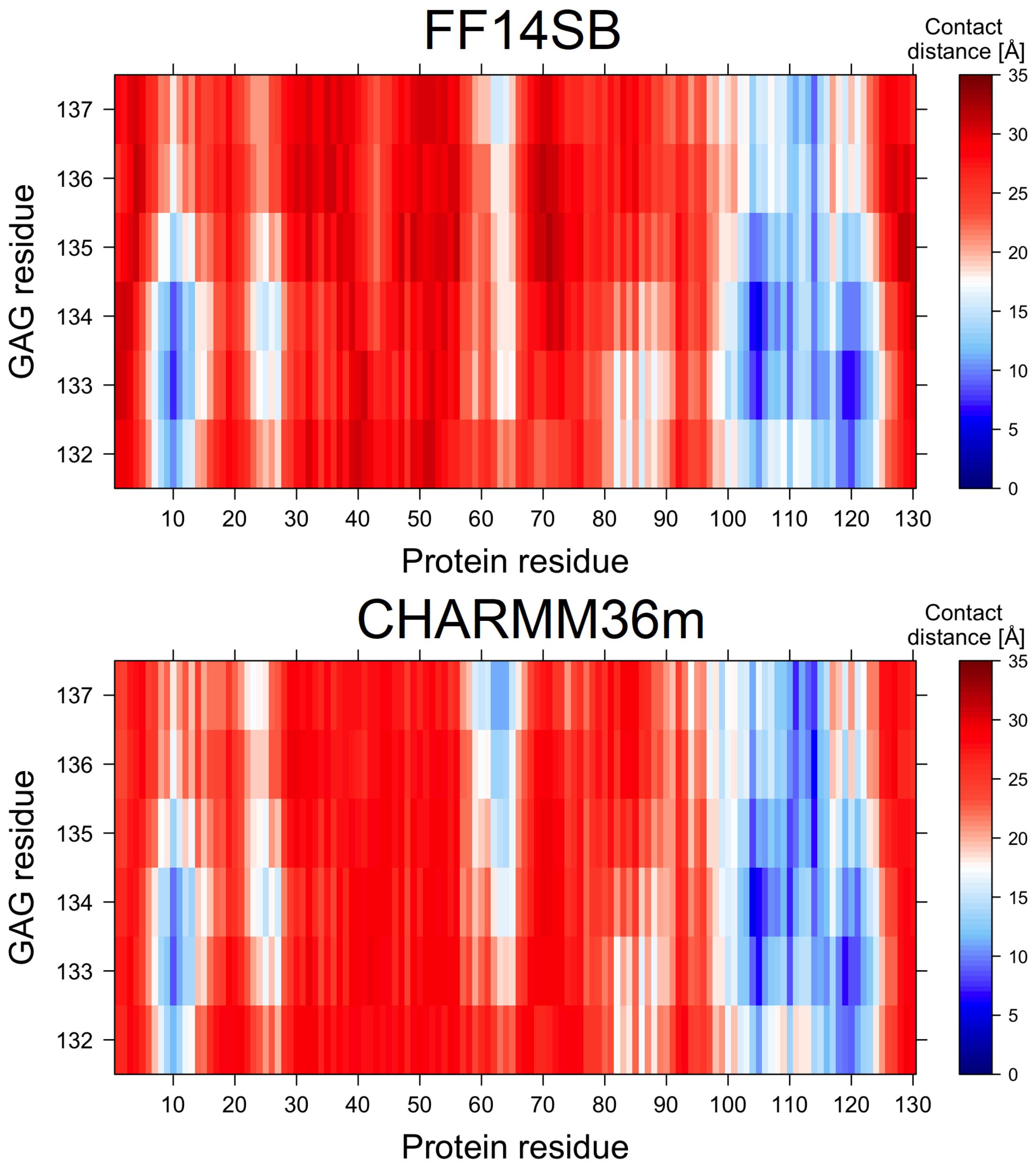

Figure S2: Distance contact maps for the FGF-1-HP dp6 complex (PDB ID: 2AXM) with the ff14SB/GLYCAM06j-1 and CHARMM36m force fields. Values on the contact maps are calculated as mean distances from whole MD simulation.

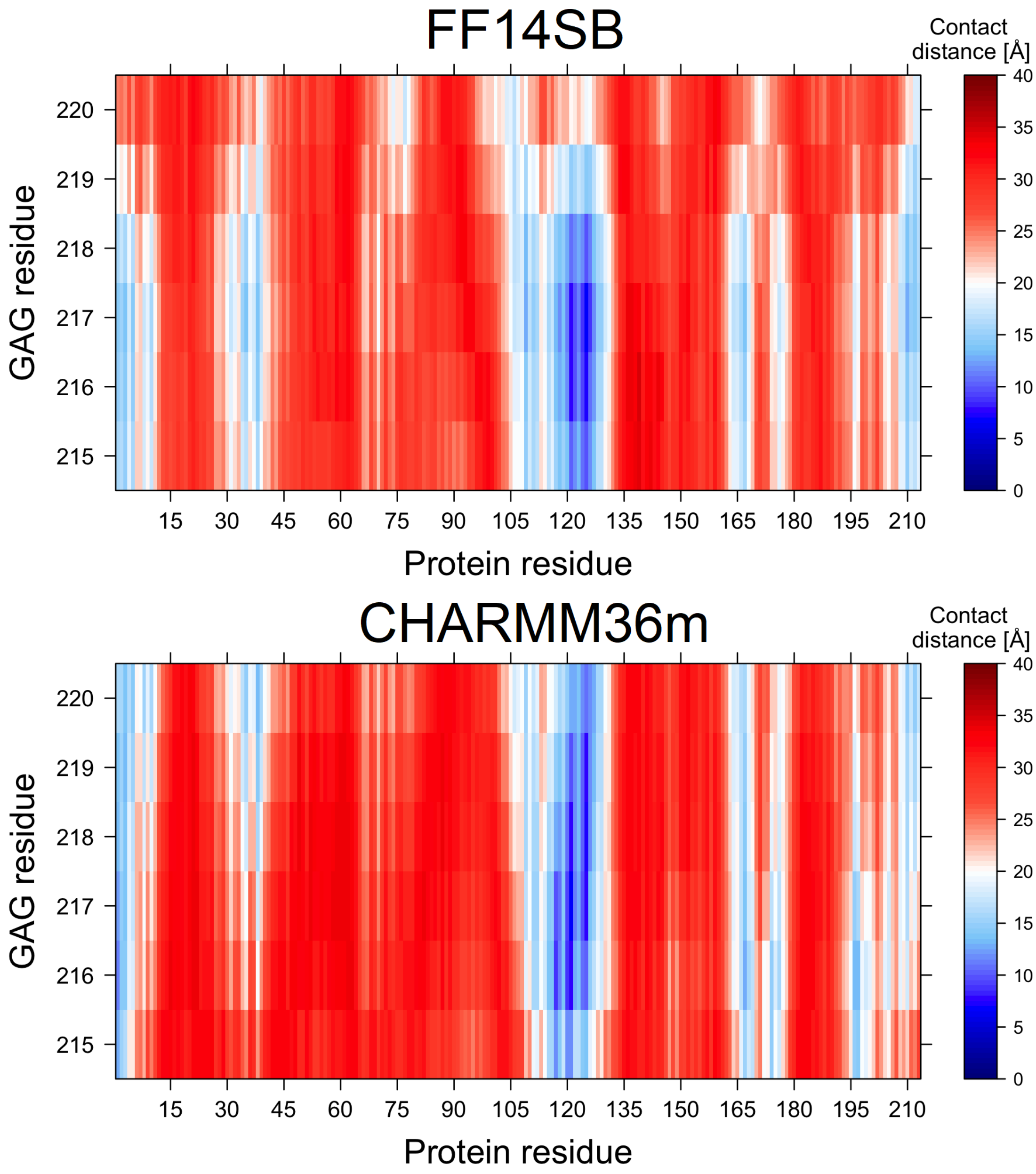

Figure S3: Distance contact maps for the CatK-C4-S dp6 complex (PDB ID: 4N8W) with the ff14SB/GLYCAM06j-1 and CHARMM36m force fields. Values on the contact maps are calculated as mean distances from whole MD simulation.

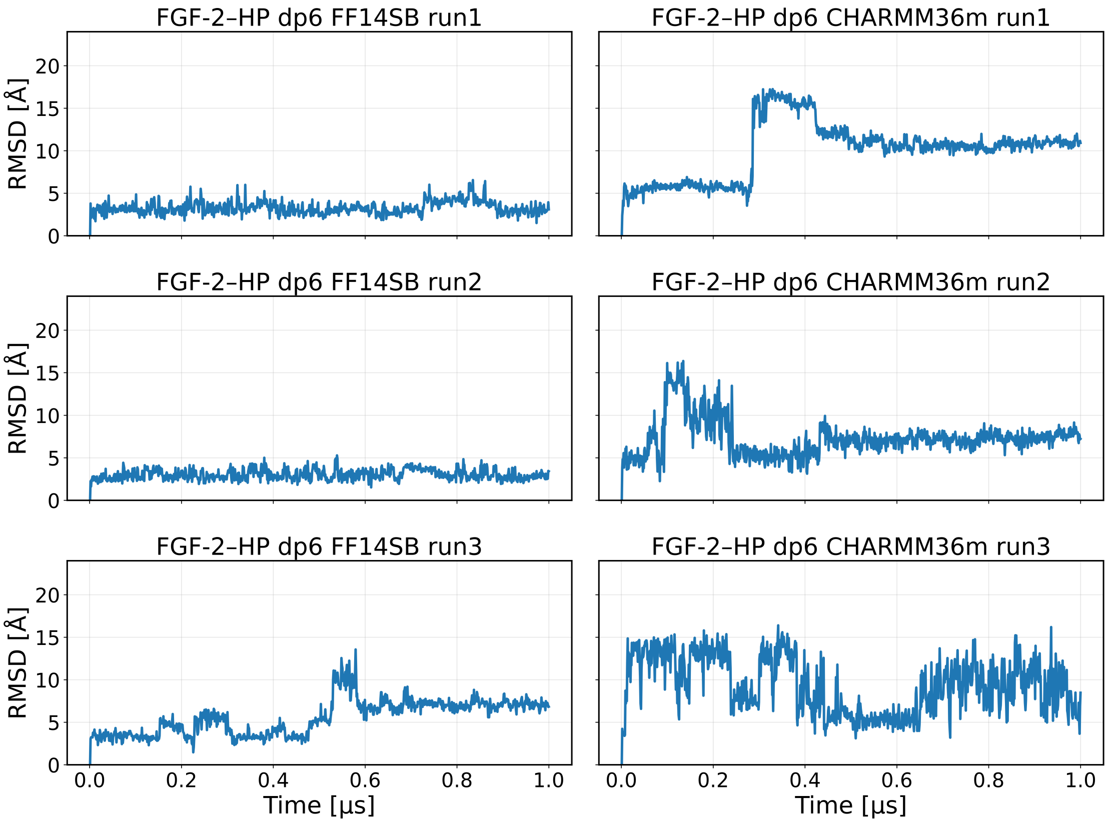

Figure S4: RMSD results for the GAG in the FGF2-HP dp6 (PDB ID: 1BFC) complexes with the ff14SB/GLYCAM06j-1 and CHARMM36m force fields.

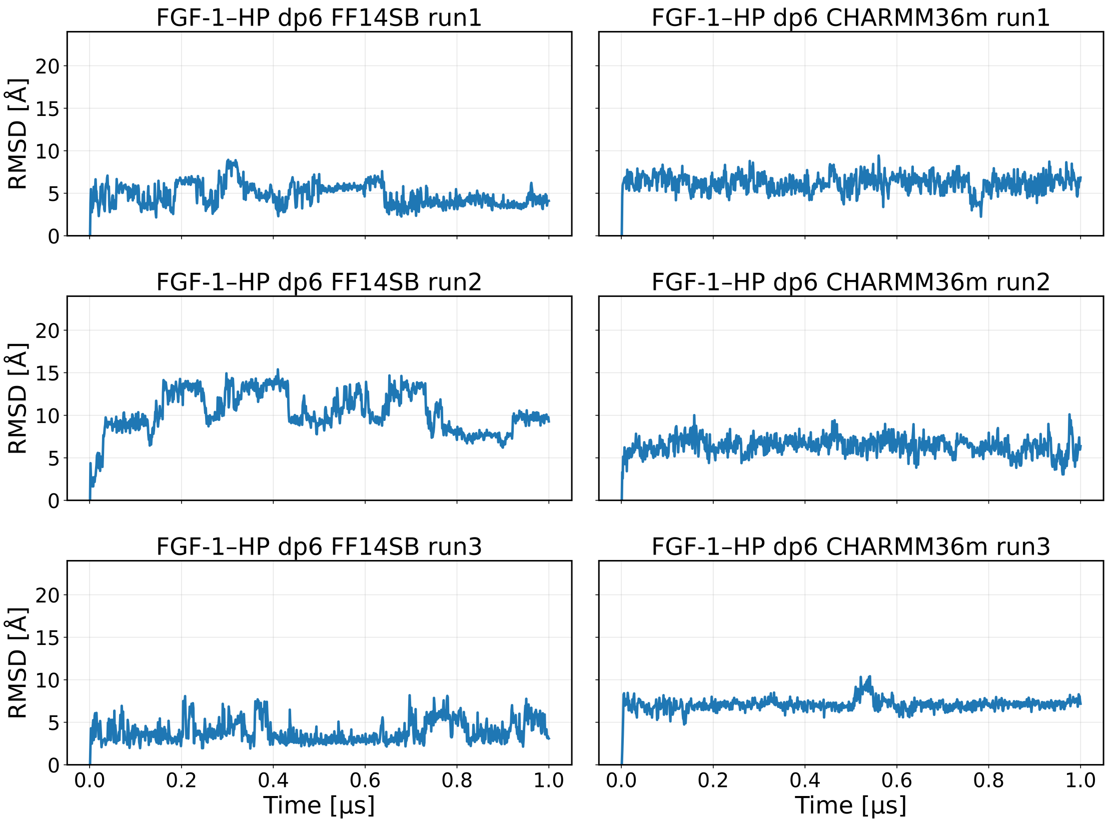

Figure S5: RMSD results for the GAG in the FGF-1-HP dp6 (PDB ID: 2AXM) complexes with the ff14SB/GLYCAM06j-1 and CHARMM36m force fields.

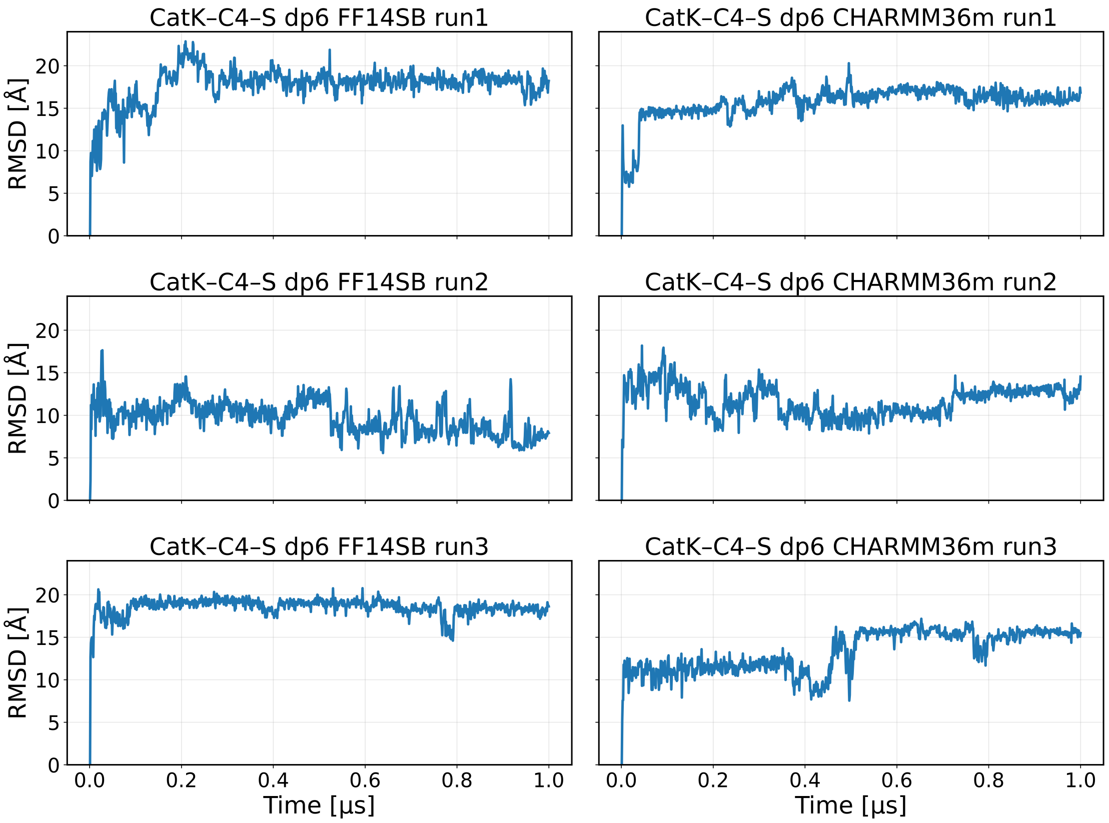

Figure S6: RMSD results for the GAG in the CatK-C4-S dp6 (PDB ID: 4N8W) complexes with the ff14SB/GLYCAM06j-1 and CHARMM36m force fields.

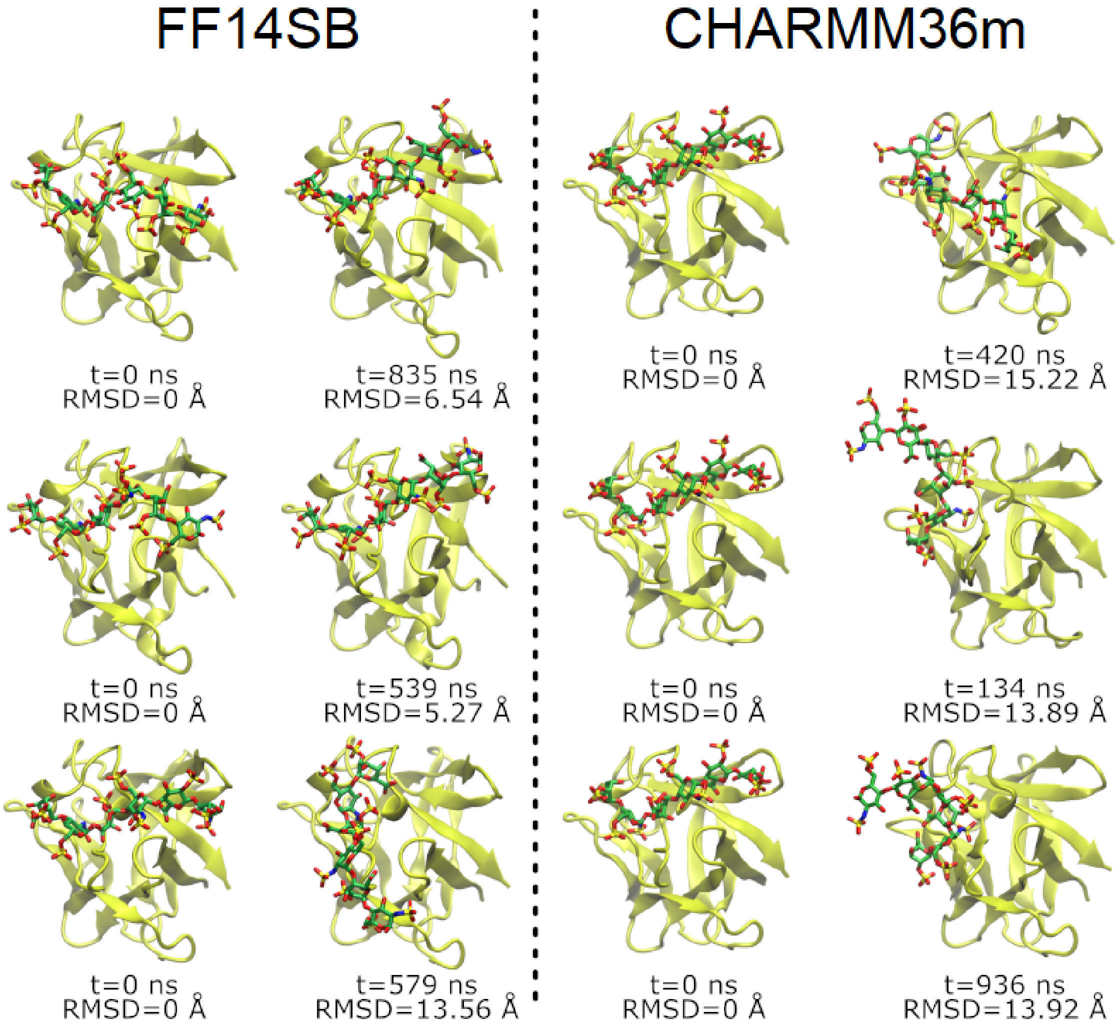

Figure S7: FGF-2-HP dp6 (PDB ID: 1BFC) initial structures and structures characterised by highest RMSD values for GAG in ff14SB/GLYCAM06j-1 and CHARMM36m force fields.

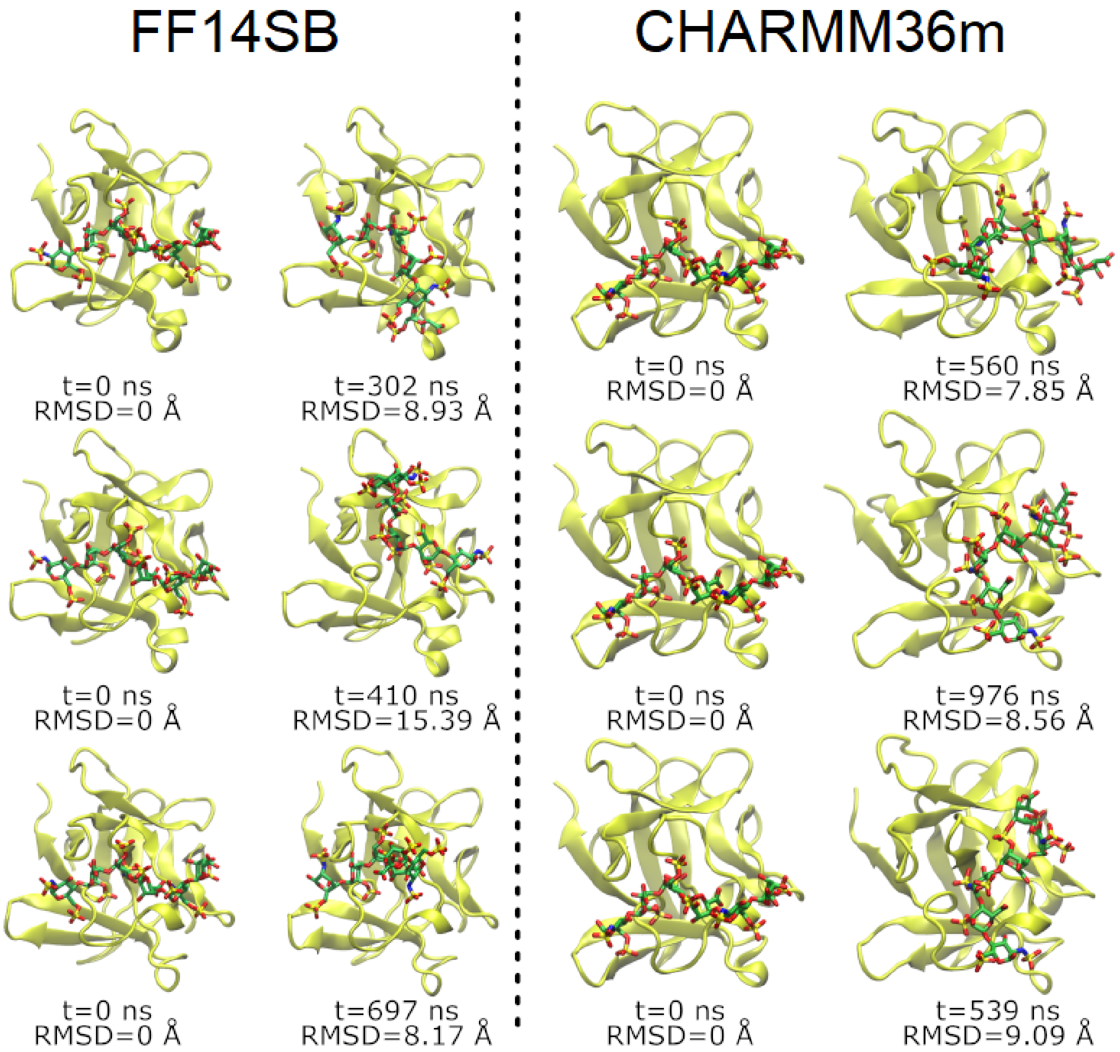

Figure S8: FGF-1-HP dp6 (PDB ID: 2AXM) initial structures and structures characterised by highest RMSD values for GAG in ff14SB/GLYCAM06j-1 and CHARMM36m force fields.

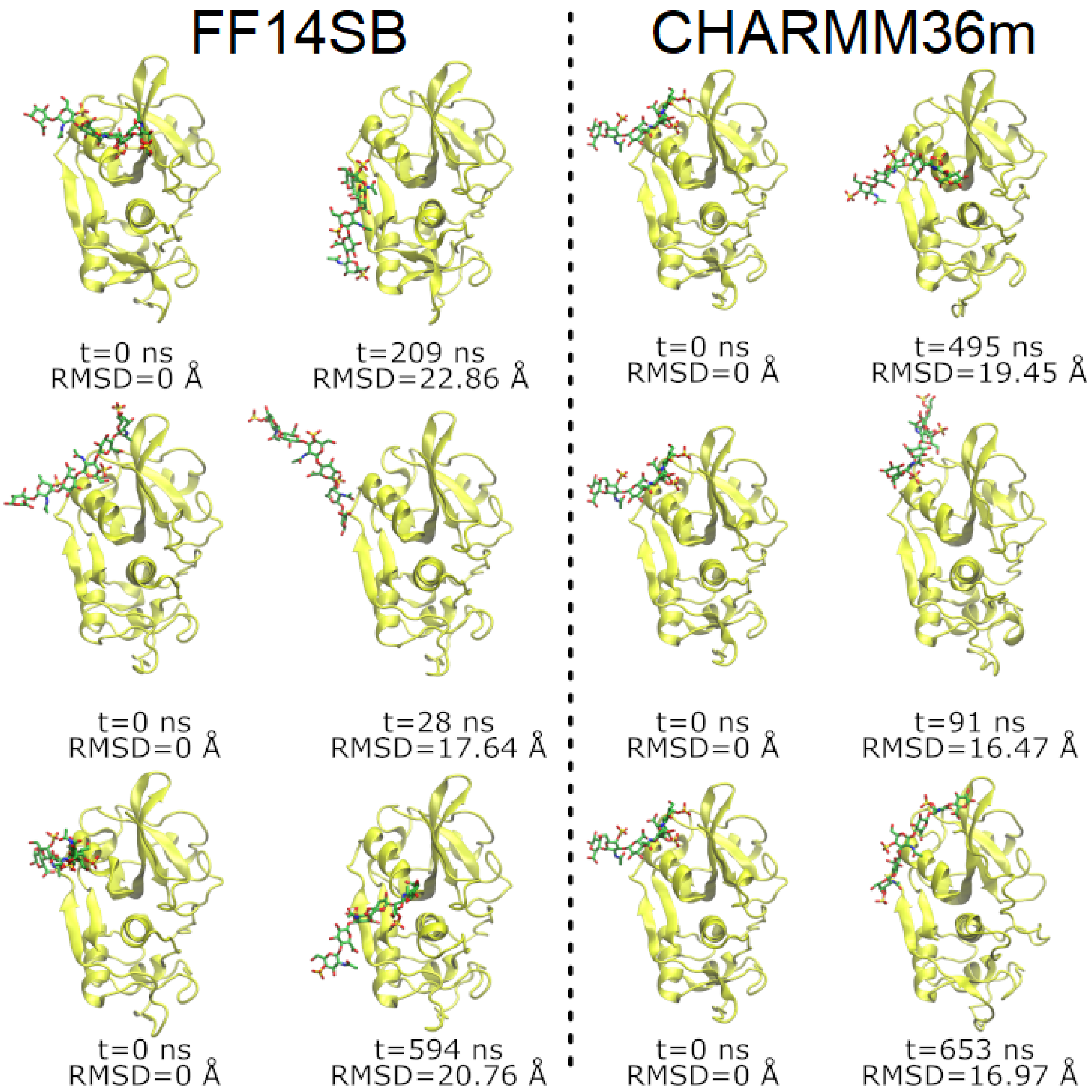

Figure S9: CatK-C4-S dp6 (PDB ID: 4N8W) initial structures and structures characterised by highest RMSD values for GAG in ff14SB/GLYCAM06j-1 and CHARMM36m force fields.

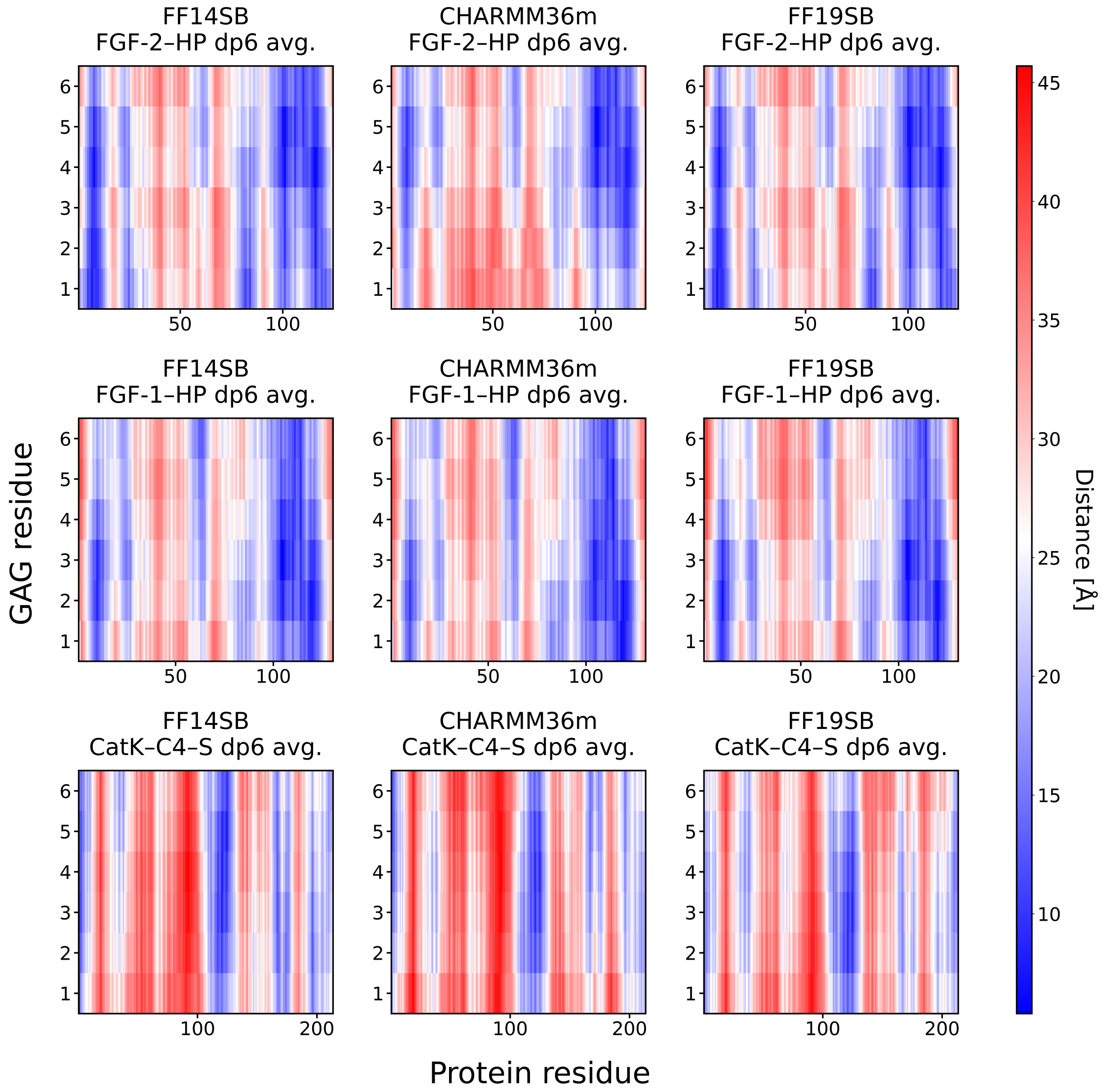

Figure S10: Averaged distance contact maps over three MD simulations with 15 Å solvent box for the FGF-1-HP dp6 (PDB ID: 1BFC), FGF-2-HP dp6 (PDB ID: 1BFC) and CatK-C4-S dp6 (PDB ID: 4N8W) with the ff14SB/GLYCAM06j-1, CHARMM36m and ff19SB/GLYCAM06j-1 force fields. Values on the contact maps are calculated as mean distances from whole MD simulation.

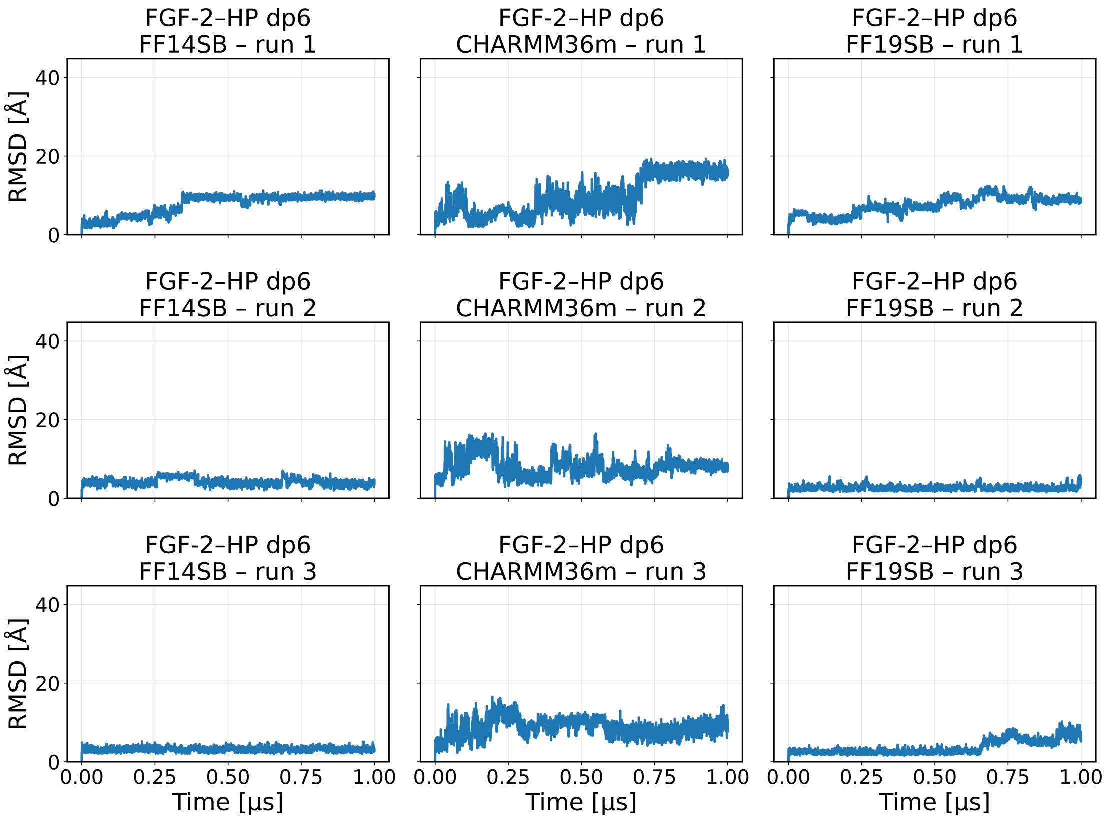

Figure S11: RMSD results for the GAG in the FGF2-HP dp6 (PDB ID: 1BFC) complexes with the ff14SB/GLYCAM06j-1, CHARMM36m and ff19SB/GLYCAM06j-1 force fields from MD simulations with 15 Å solvent box.

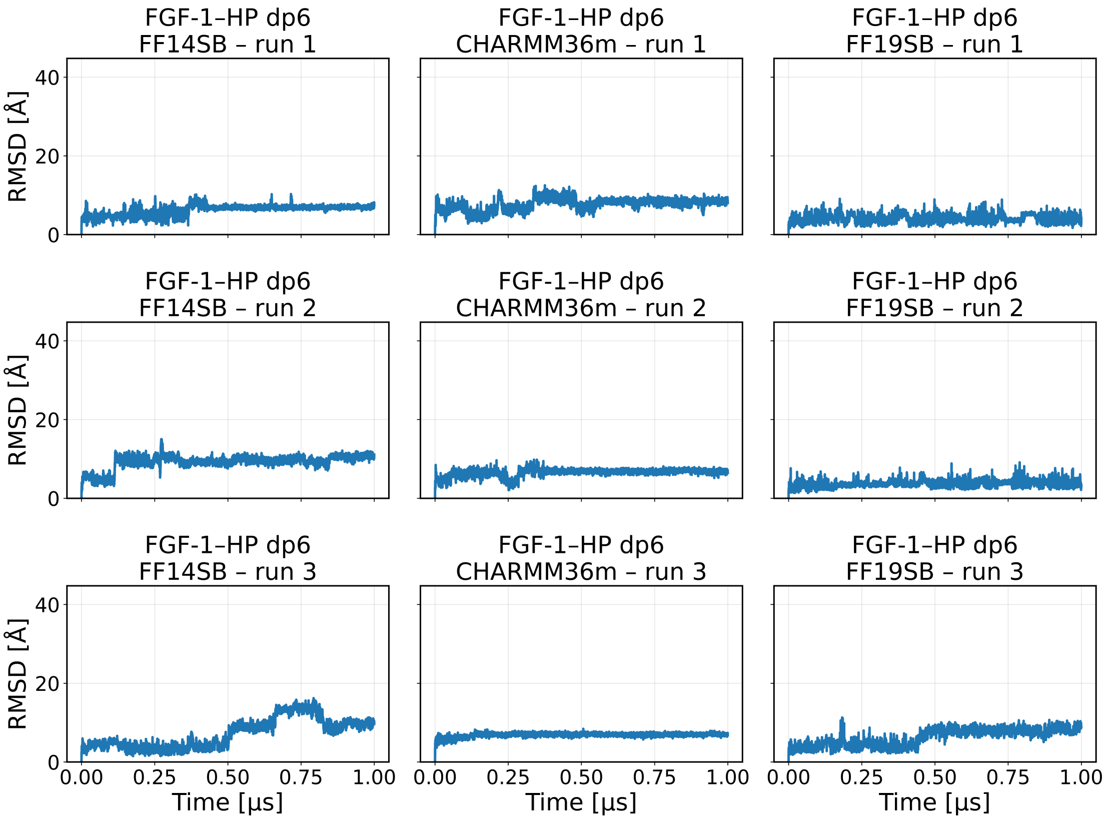

Figure S12: RMSD results for the GAG in the FGF1-HP dp6 (PDB ID: 2AXM) complexes with the ff14SB/GLYCAM06j-1, CHARMM36m and ff19SB/GLYCAM06j-1 force fields from MD simulations with 15 Å solvent box.

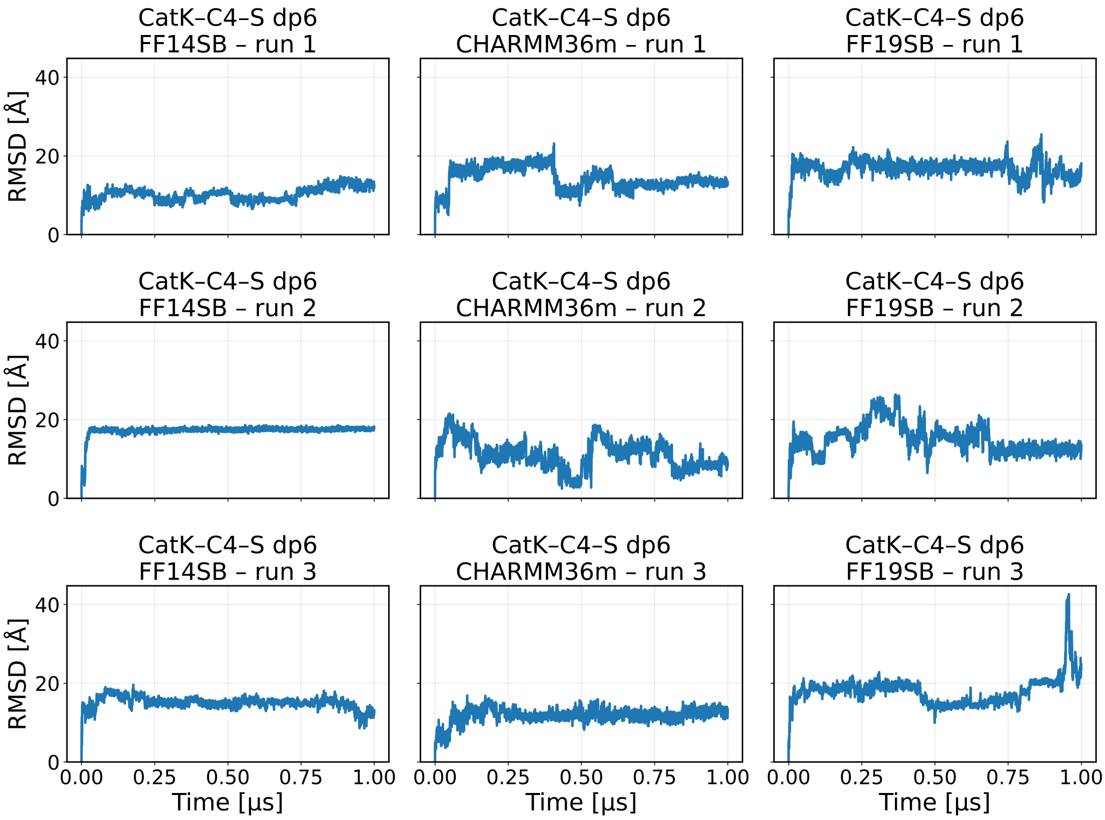

Figure S13: RMSD results for the GAG in the CatK-C4-S dp6 (PDB ID: 4N8W) complexes with the ff14SB/GLYCAM06j-1, CHARMM36m and ff19SB/GLYCAM06j-1 force fields from MD simulations with 15 Å solvent box.

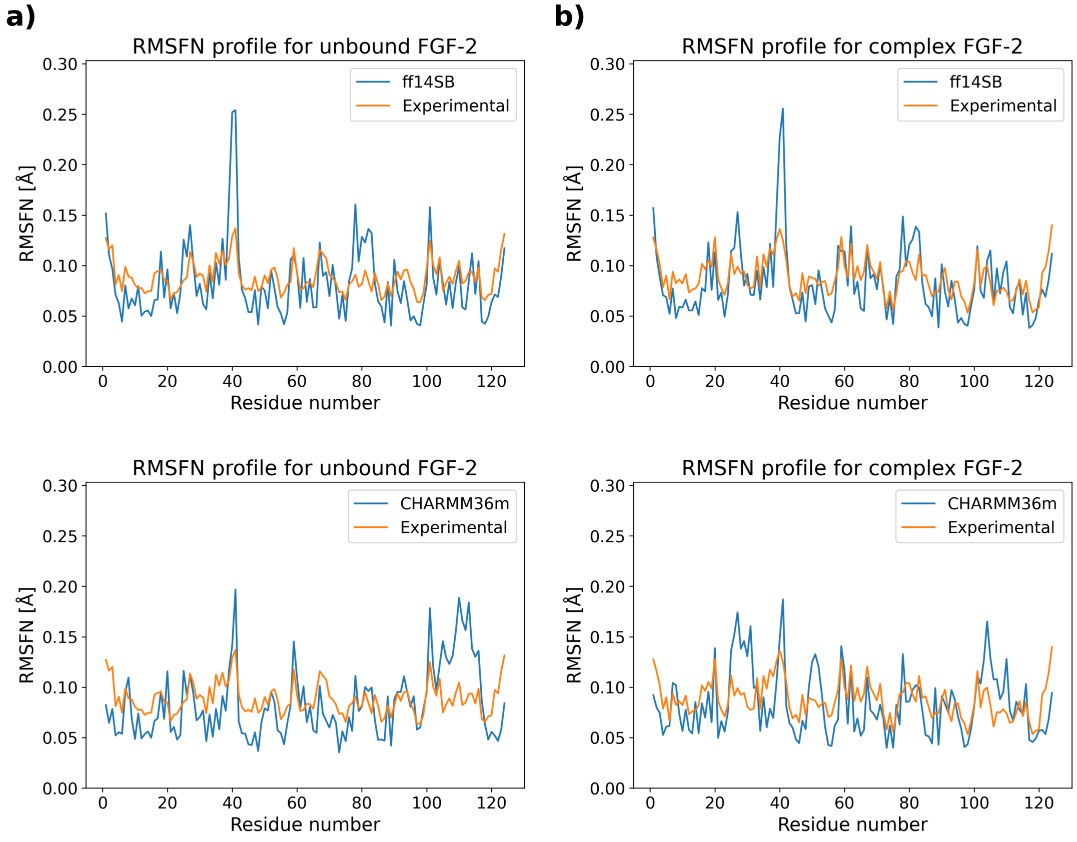

Figure S14: Fluctuation profiles for FGF-2 residues in unbound state (a) and in complex with HP dp6 (b) for ff14SB/GLYCAM06j-1, CHARMM36m force fields (blue) and experimental data (orange).

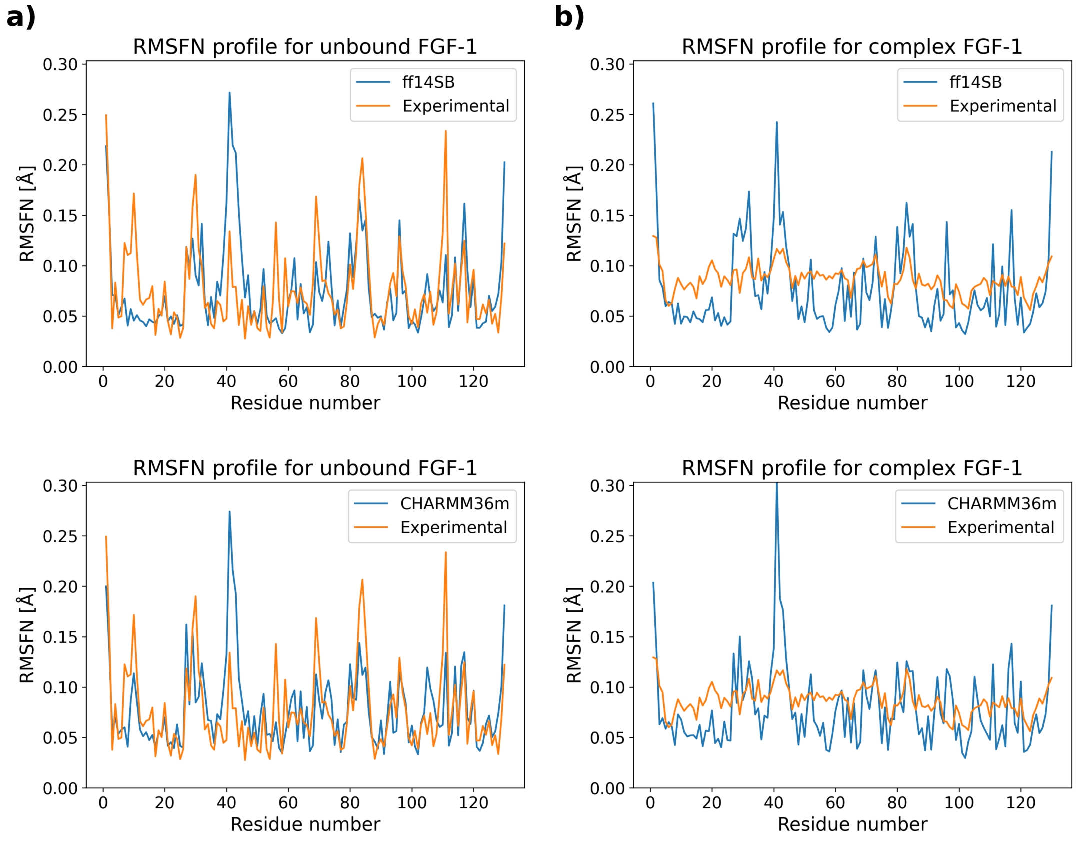

Figure S15: Fluctuation profiles for FGF-1 residues in unbound state (a) and in complex with HP dp6 (b) for ff14SB/GLYCAM06j-1, CHARMM36m force fields (blue) and experimental data (orange).

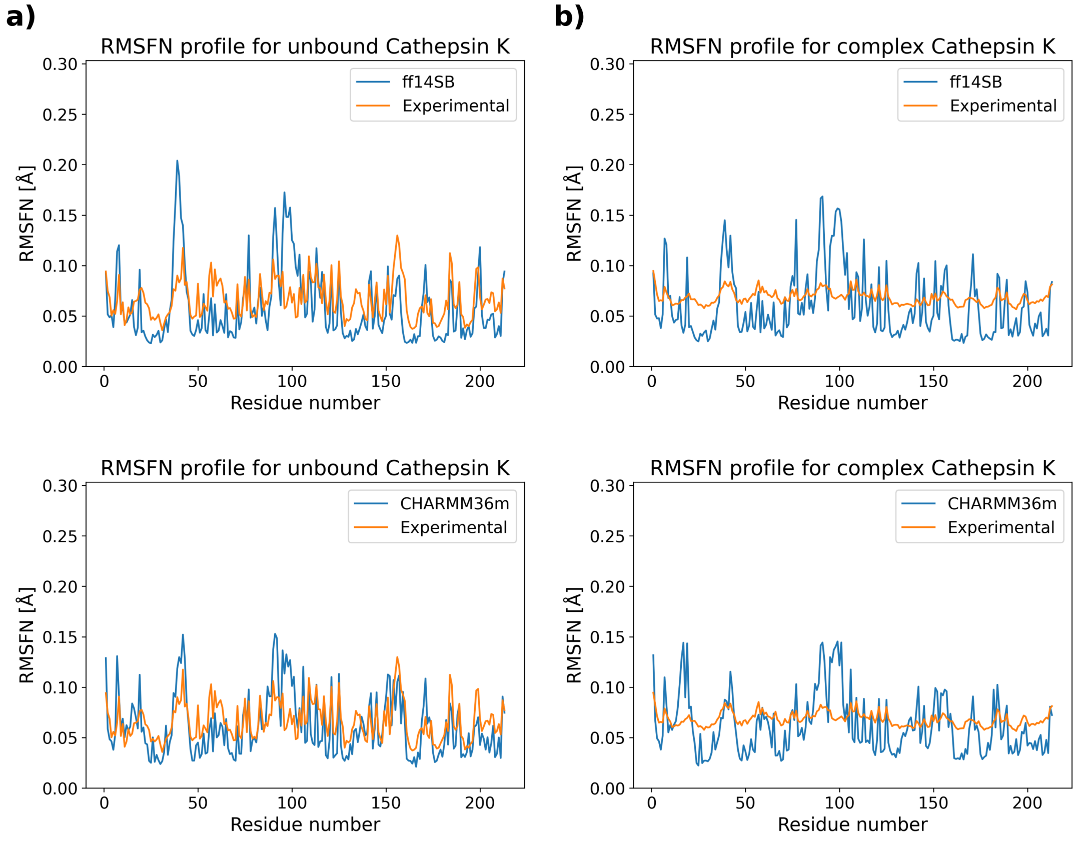

Figure S16: Fluctuation profiles for CatK residues in unbound state (a) and in complex with C4-S dp6 (b) for ff14SB/GLYCAM06j-1, CHARMM36m force fields (blue) and experimental data (orange).

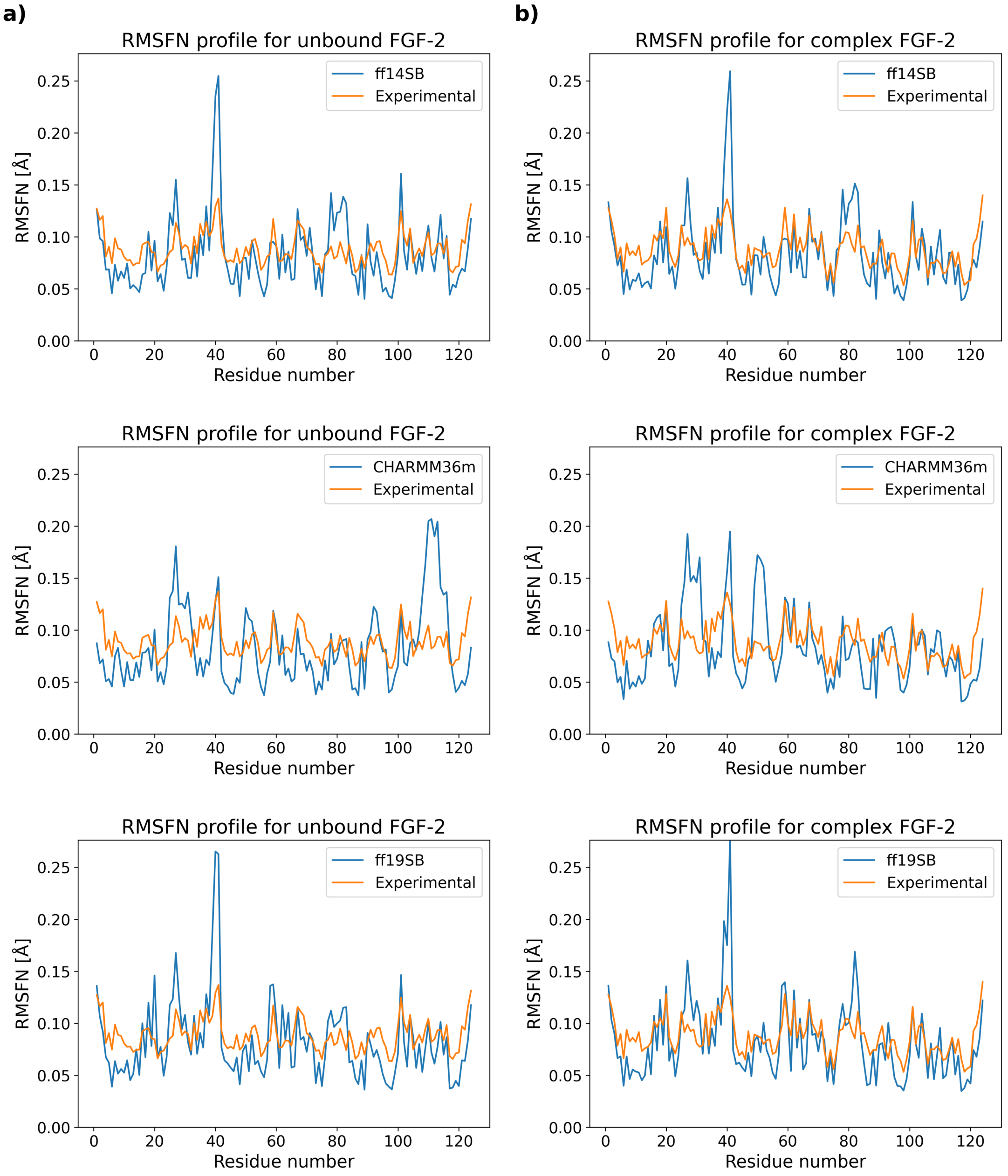

Figure S17: Fluctuation profiles for FGF-2 residues in unbound state (a) and in complex with HP dp6 (b) for ff14SB/GLYCAM06j-1, CHARMM36m, ff19SB/GLYCAM06j-1 force fields (blue) and experimental data (orange) for simulations with 15 Å solvent box.

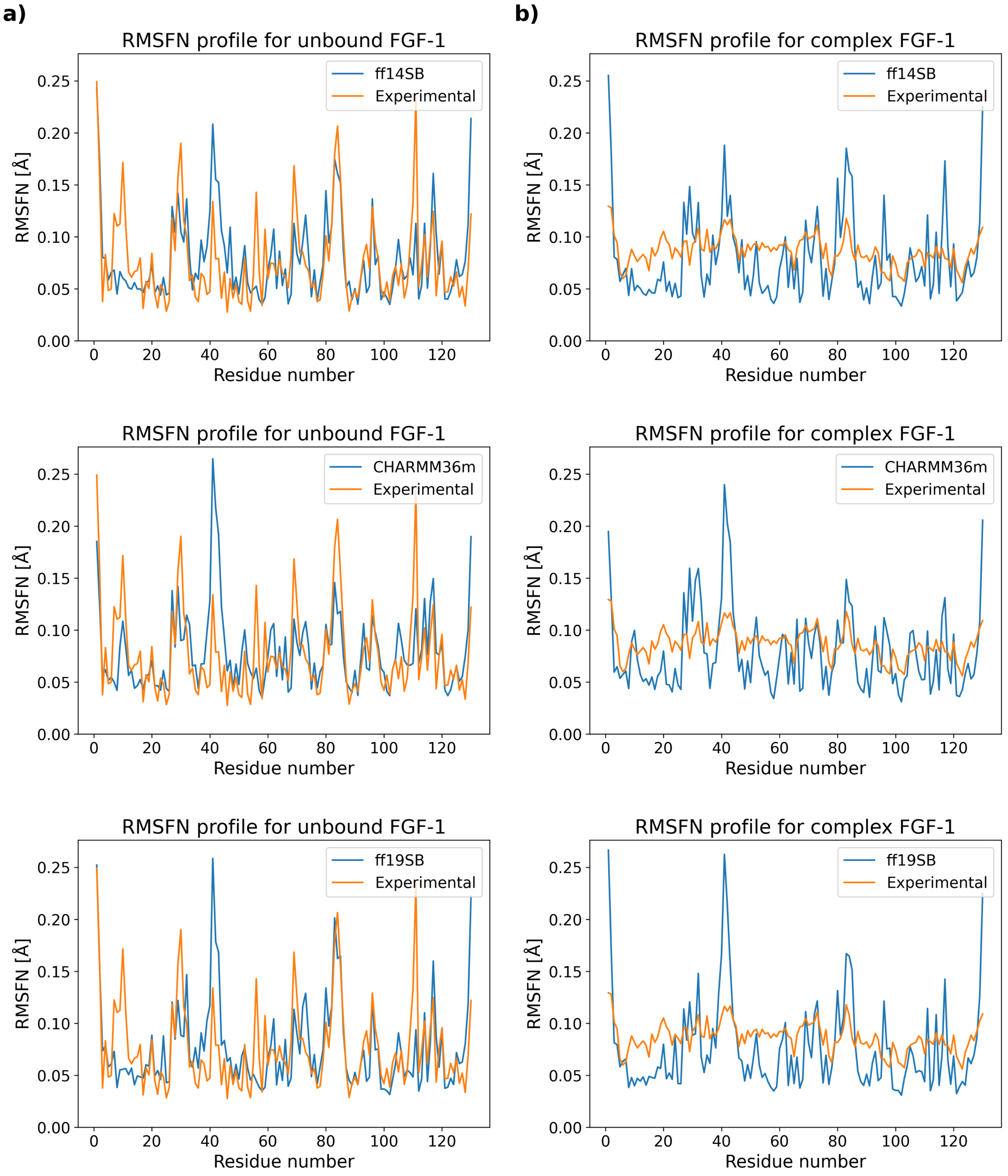

Figure S18: Fluctuation profiles for FGF-1 residues in unbound state (a) and in complex with HP dp6 (b) for ff14SB/GLYCAM06j-1, CHARMM36m, ff19SB/GLYCAM06j-1 force fields (blue) and experimental data (orange) for simulations with 15 Å solvent box.

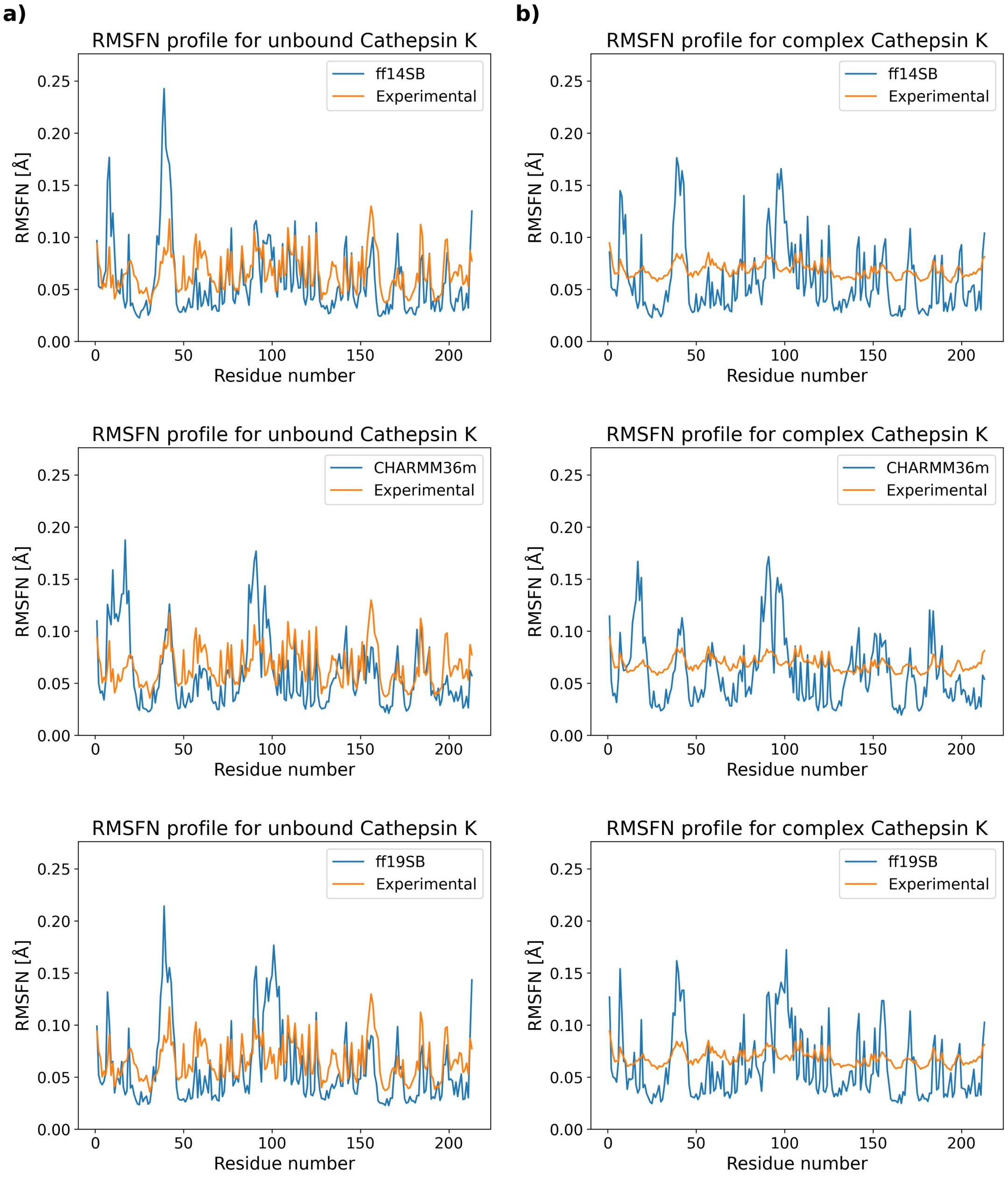

Figure S19: Fluctuation profiles for CatK residues in unbound state (a) and in complex with HP dp6 (b) for ff14SB/GLYCAM06j-1, CHARMM36m, ff19SB/GLYCAM06j-1 force fields (blue) and experimental data (orange) for simulations with 15 Å solvent box.

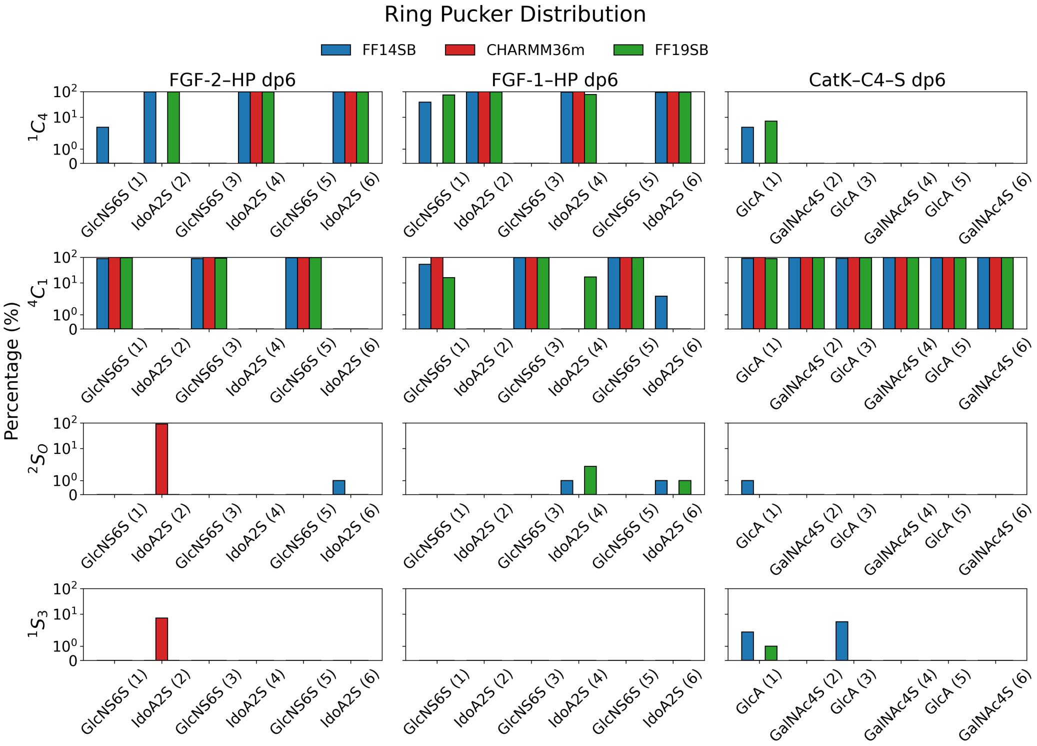

Figure S20: Ring puckering conformations of HP and C4-S residues observed in the MD simulation in ff14SB/GLYCAM06j-1, CHARMM36m and ff19SB/GLYCAM06j-1 force fields for simulations in 15 Å solvent box. The residue numbering (in parenthesis) is from the reducing to the nonreducing end.

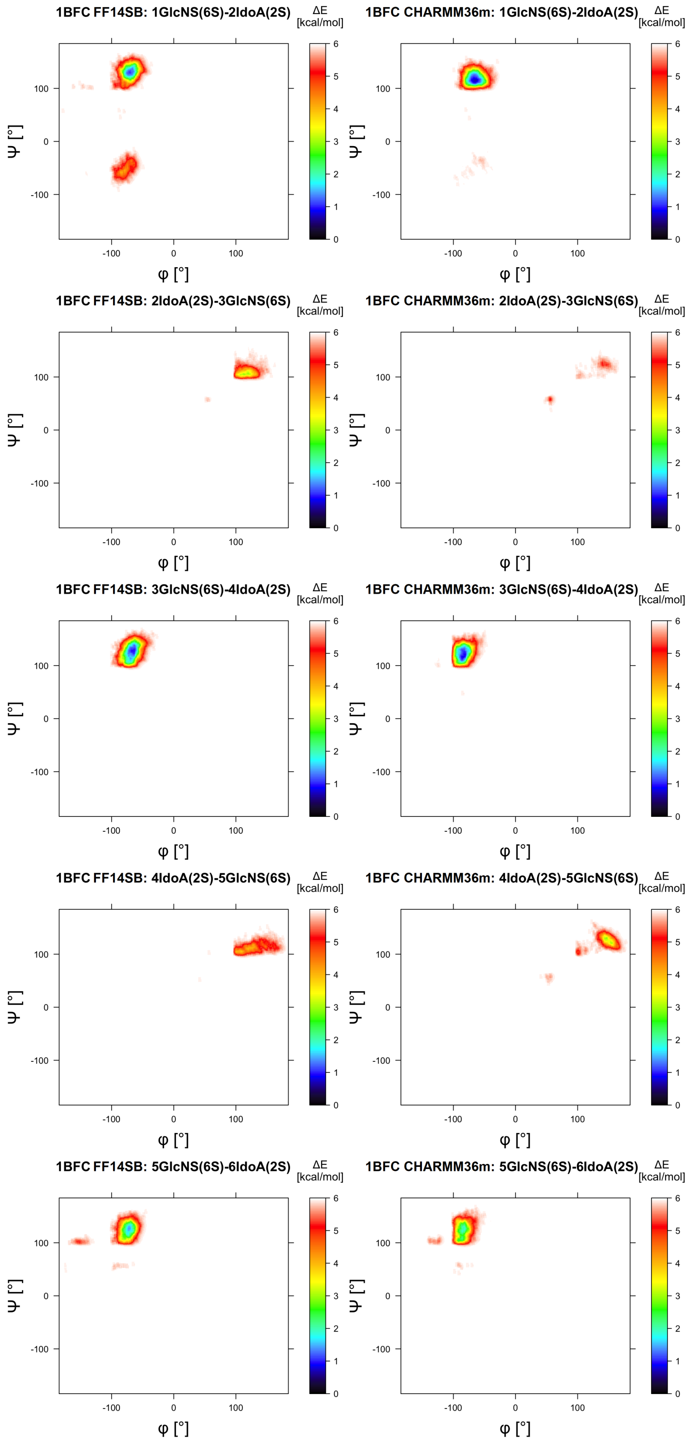

Figure S21: FGF-2-HP dp6 (PDB ID: 1BFC) glycosidic linkage heat maps for φ and ψ dihedral angles in ff14SB/GLYCAM06j-1 and CHARMM36m force fields.

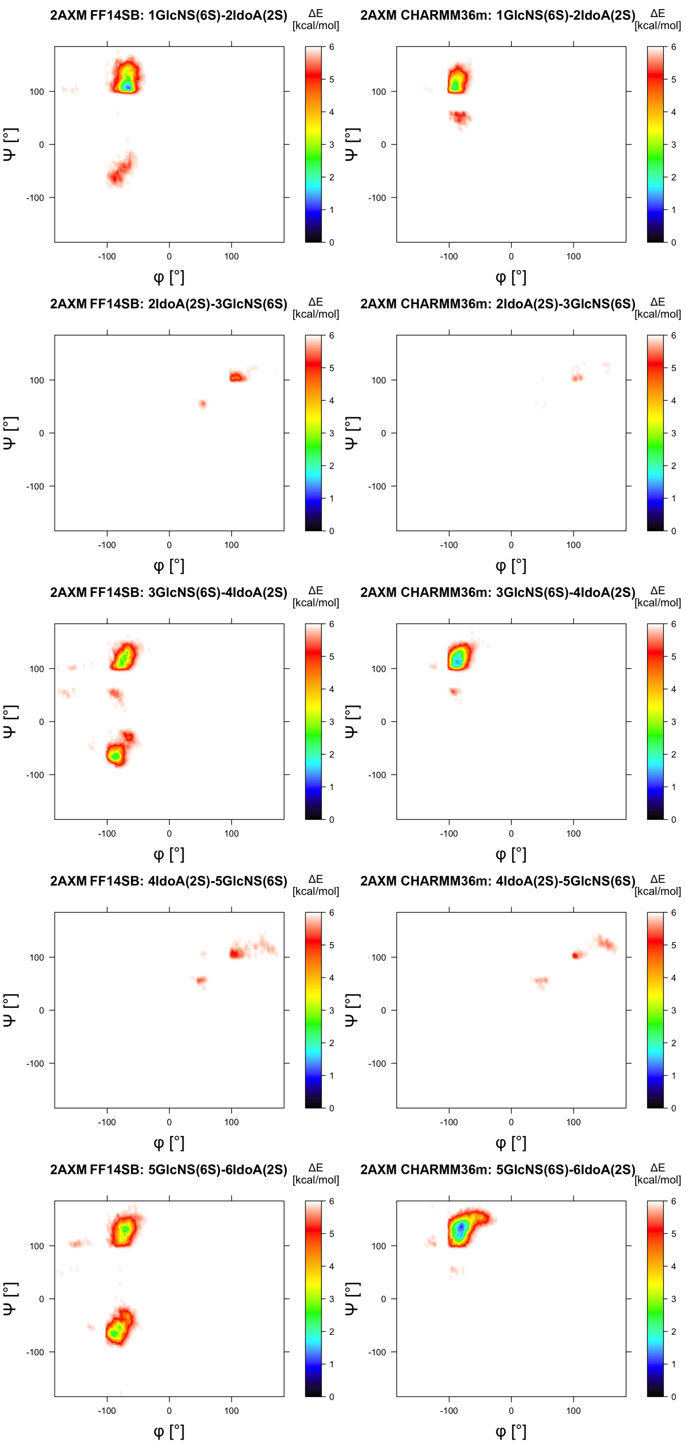

Figure S22: FGF-1-HP dp6 (PDB ID: 2AXM) glycosidic linkage heat maps for φ and ψ dihedral angles in ff14SB/GLYCAM06j-1 and CHARMM36m force fields.

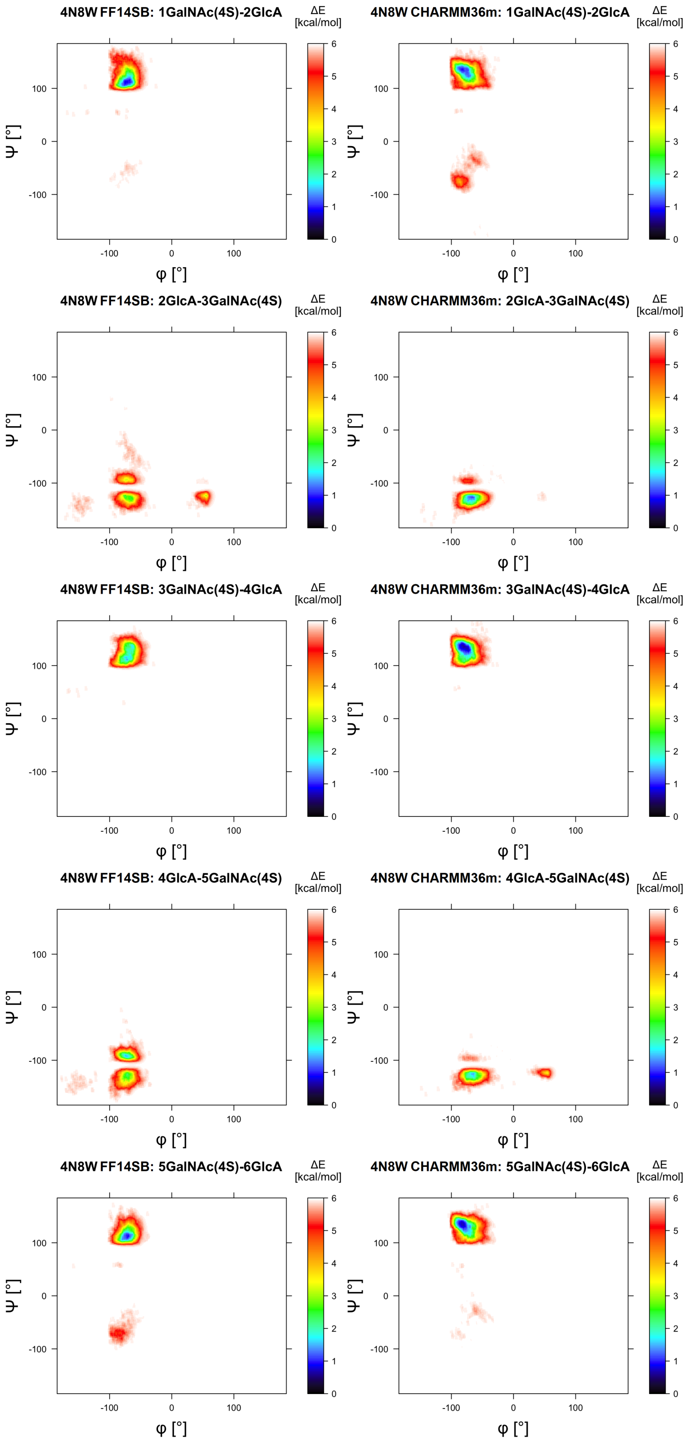

Figure S23: CatK-C4-S dp6 (PDB ID: 4N8W) glycosidic linkage heat maps for φ and ψ dihedral angles in ff14SB/GLYCAM06j-1 and CHARMM36m force fields.

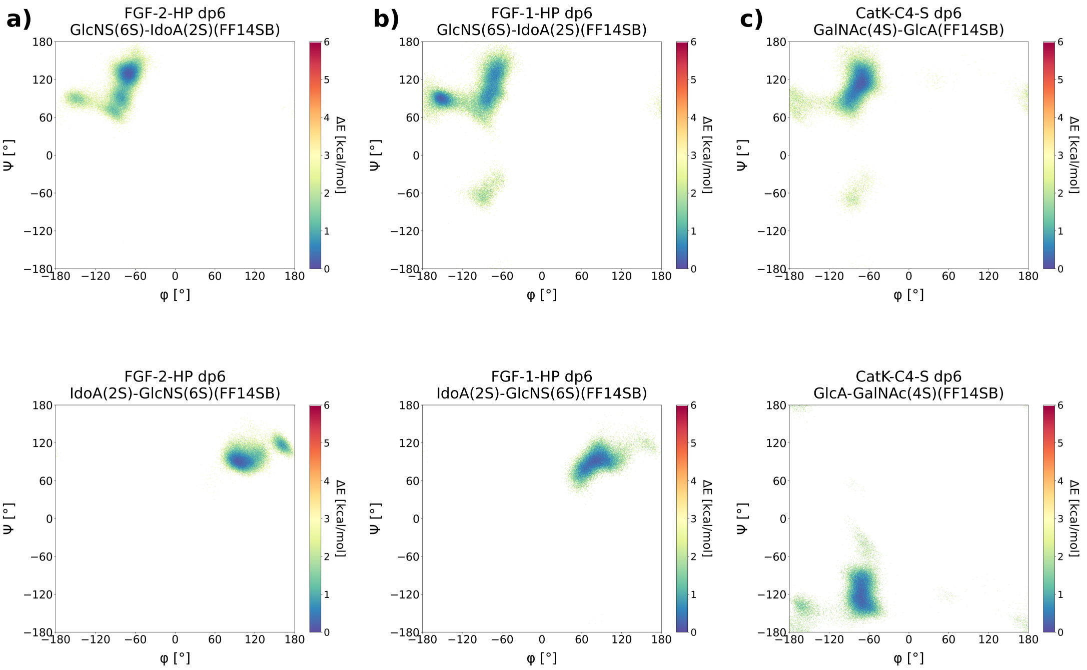

Figure S24: Heatmaps for the glycosidic linkage showing the ϕ and ψ dihedral angles for a) FGF-2-HP dp6, b) FGF-1-HP dp6 and c) CatK-C4-S dp6 using the ff14SB/GLYCAM06j-1 force field for simulations with 15 Å solvent box.

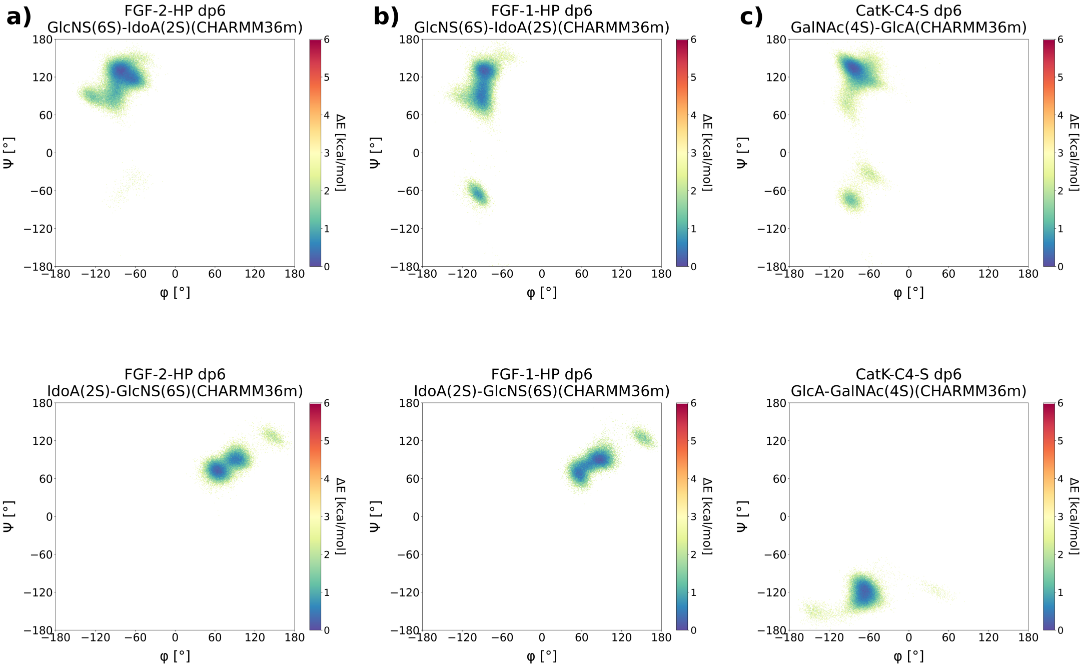

Figure S25: Heatmaps for the glycosidic linkage showing the ϕ and ψ dihedral angles for a) FGF-2-HP dp6, b) FGF-1-HP dp6 and c) CatK-C4-S dp6 using the CHARMM36m force field for simulations with 15 Å solvent box.

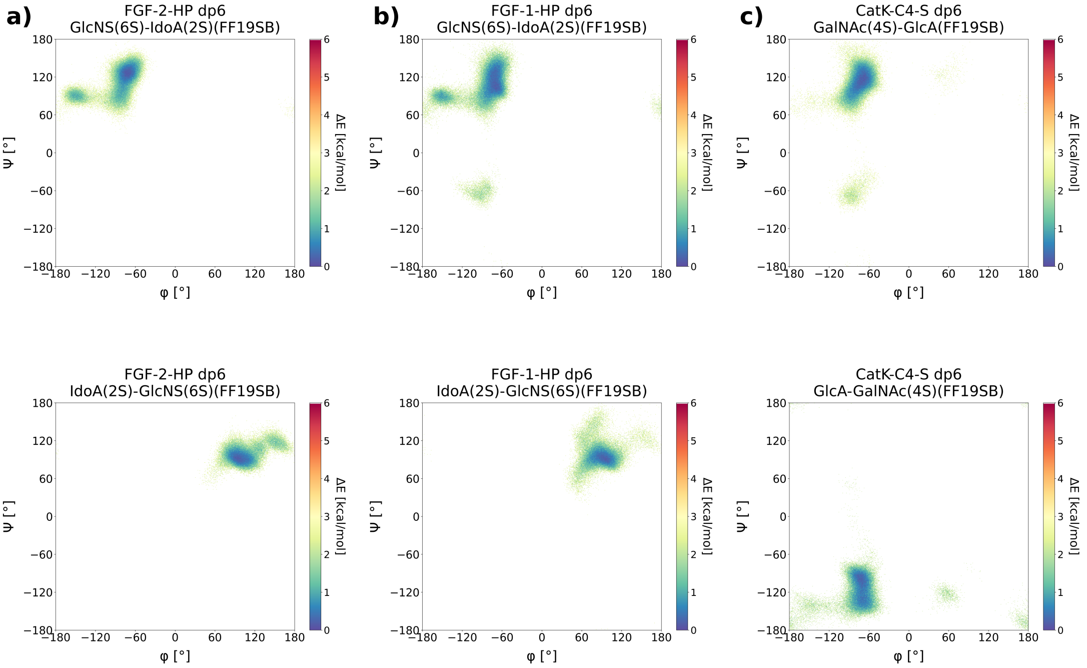

Figure S26: Heatmaps for the glycosidic linkage showing the ϕ and ψ dihedral angles for a) FGF-2-HP dp6, b) FGF-1-HP dp6 and c) CatK-C4-S dp6 using the ff19SB/GLYCAM06j-1 force field for simulations with 15 Å solvent box.

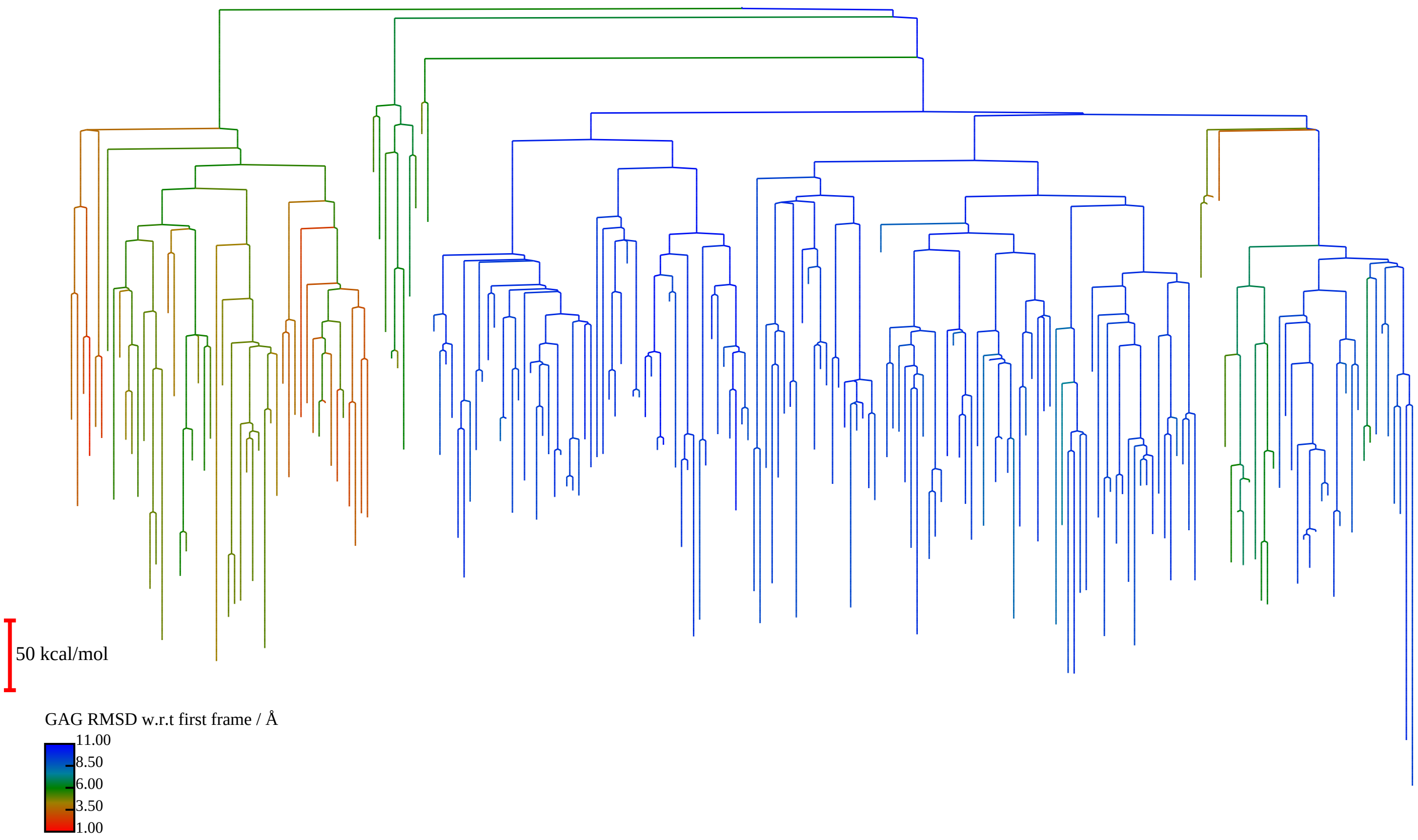

Figure S27: MDDG ff14SB/GLYCAM06j-1 disconnectivity graph for the 1BFC complex.

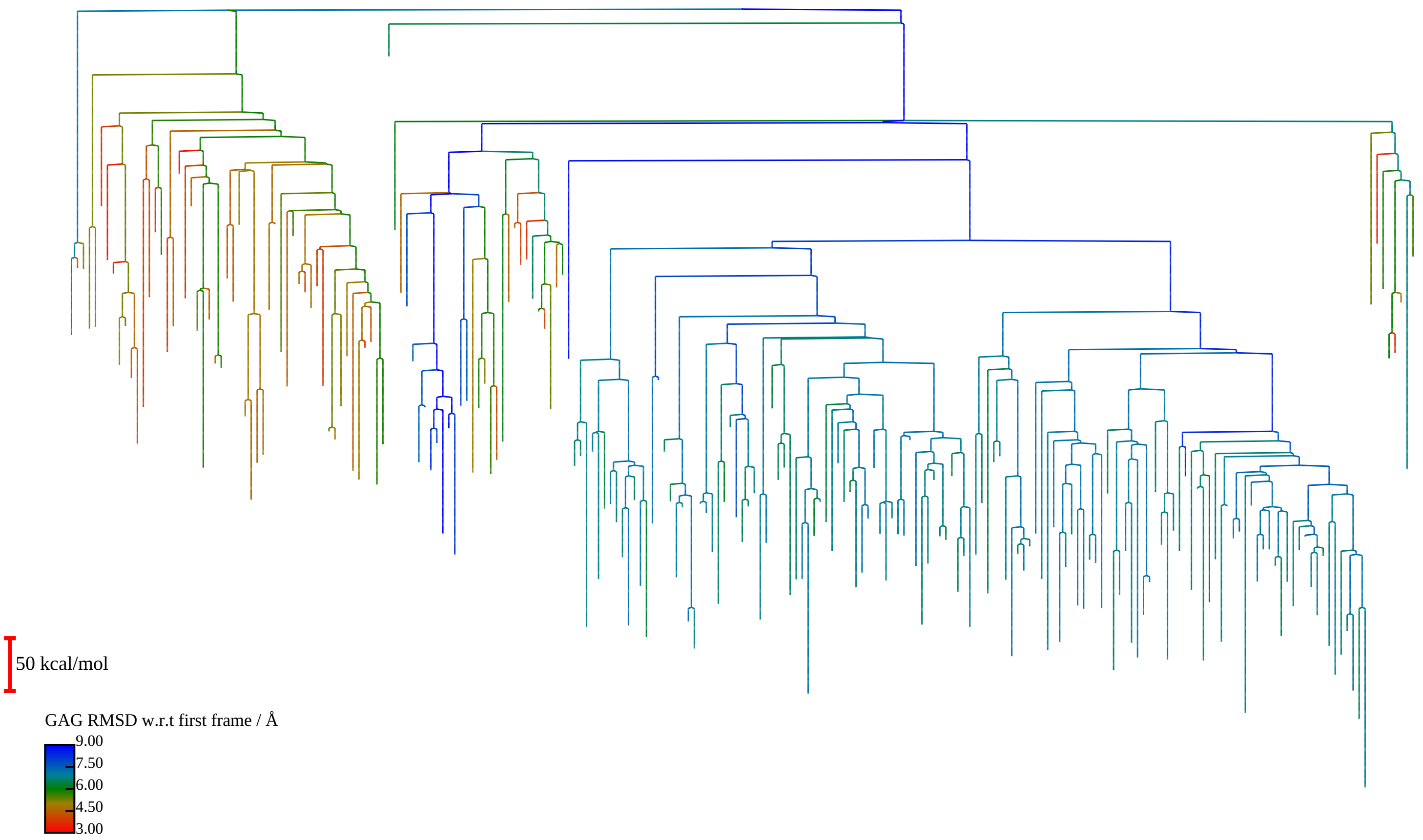

Figure S28: MDDG ff14SB/GLYCAM06j-1 disconnectivity graph for the 2AXM complex.

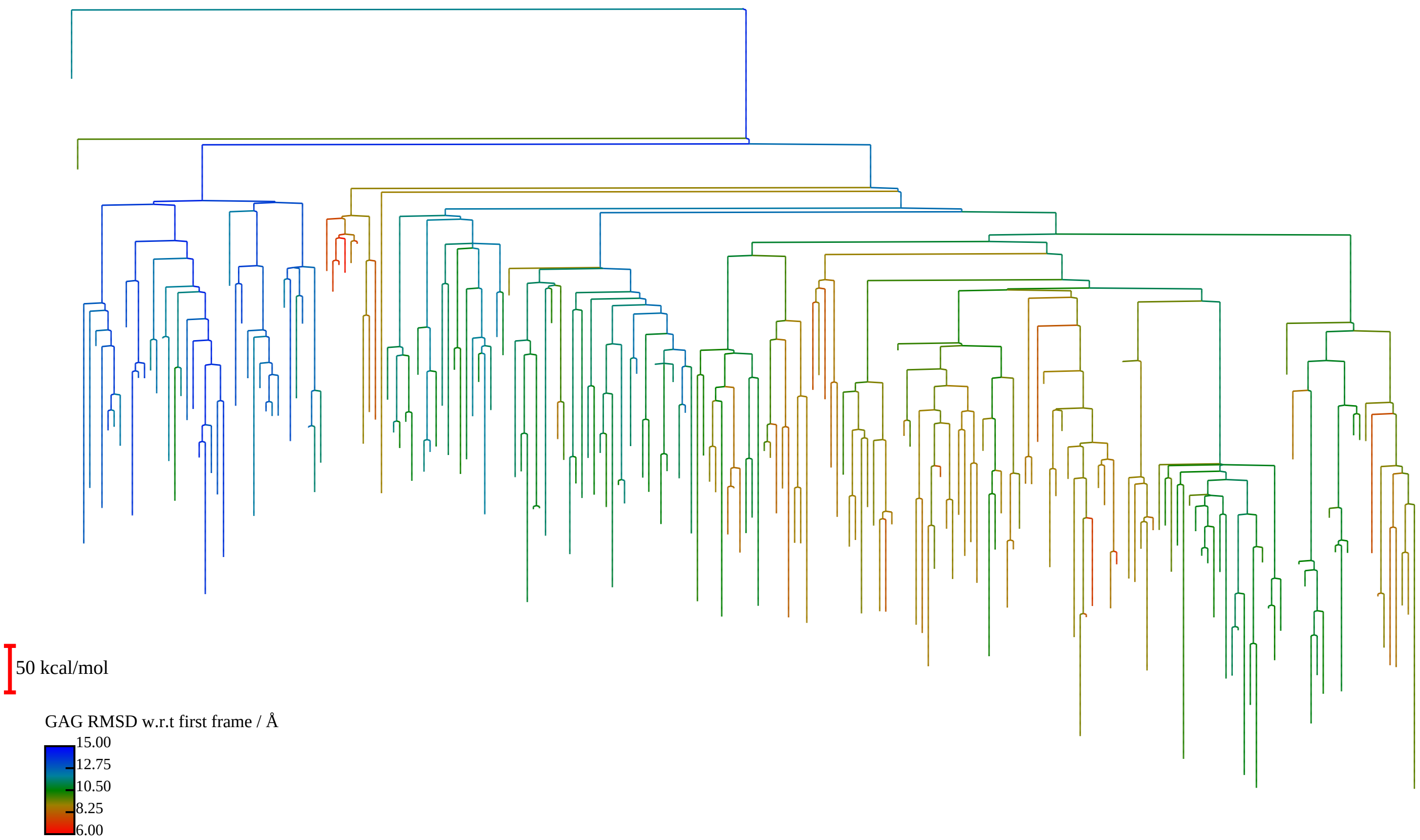

Figure S29: MDDG ff14SB/GLYCAM06j-1 disconnectivity graph for the 4N8W complex.

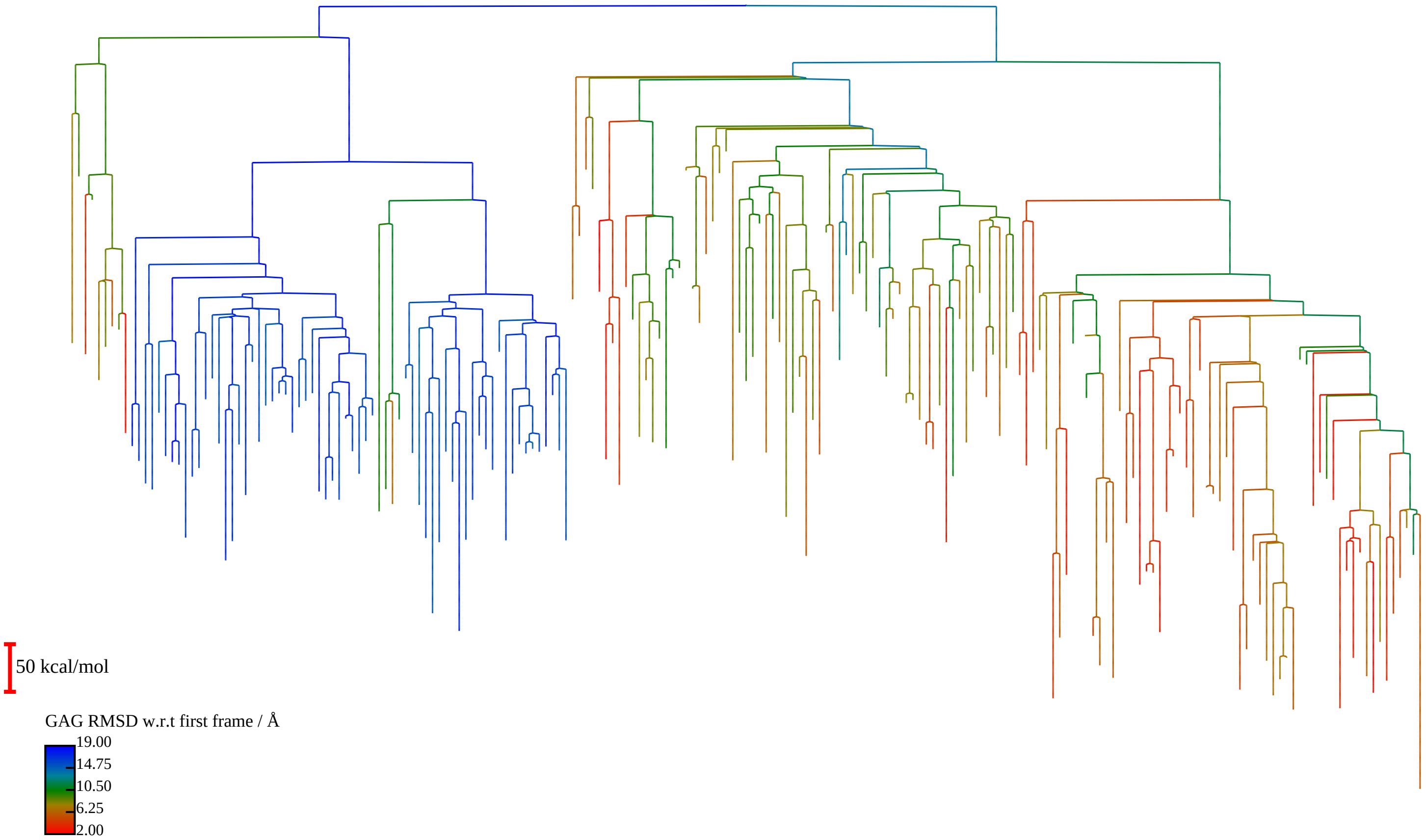

Figure S30: MDDG CHARMM disconnectivity graph for the 1BFC complex.

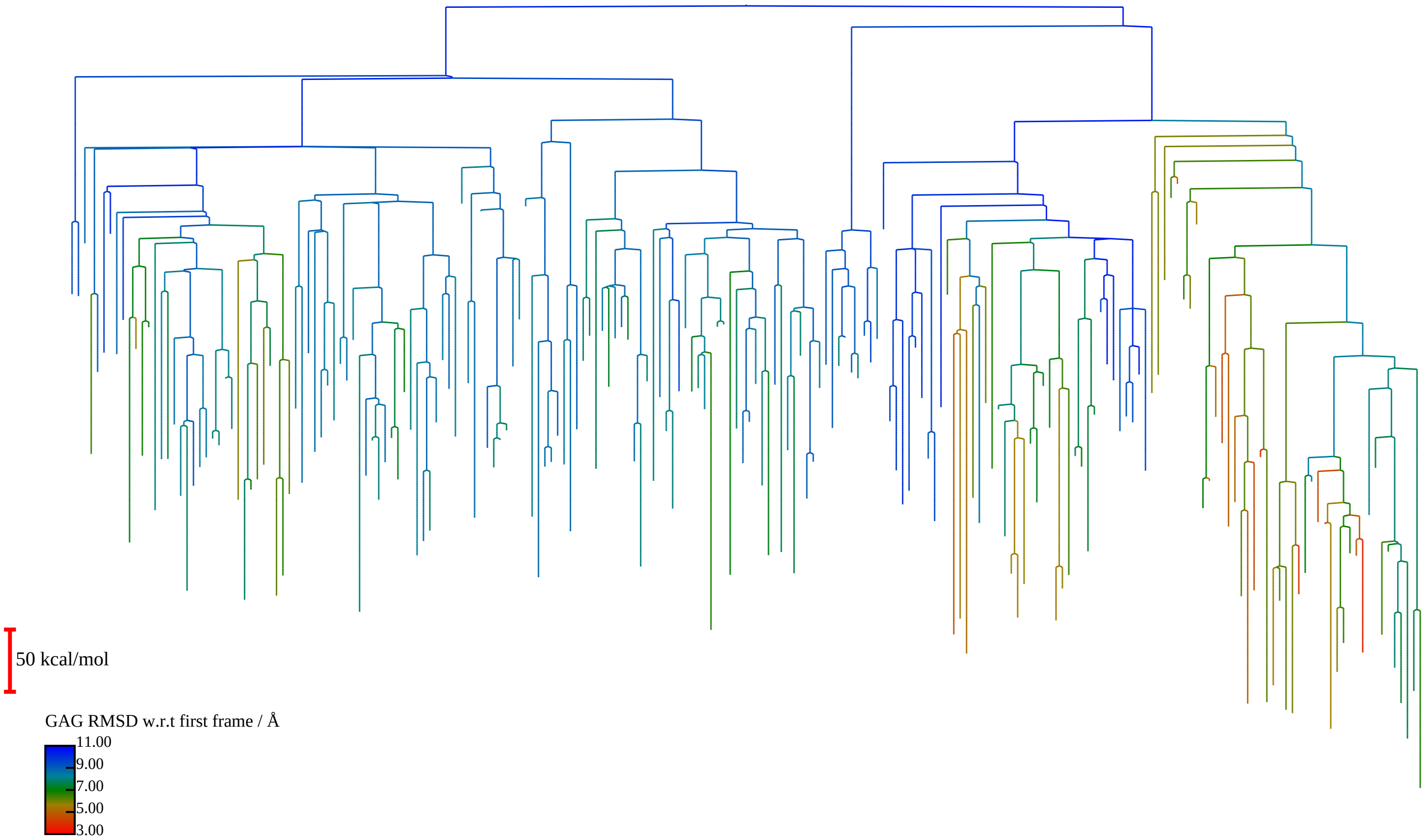

Figure S31: MDDG CHARMM disconnectivity graph for the 2AXM complex.

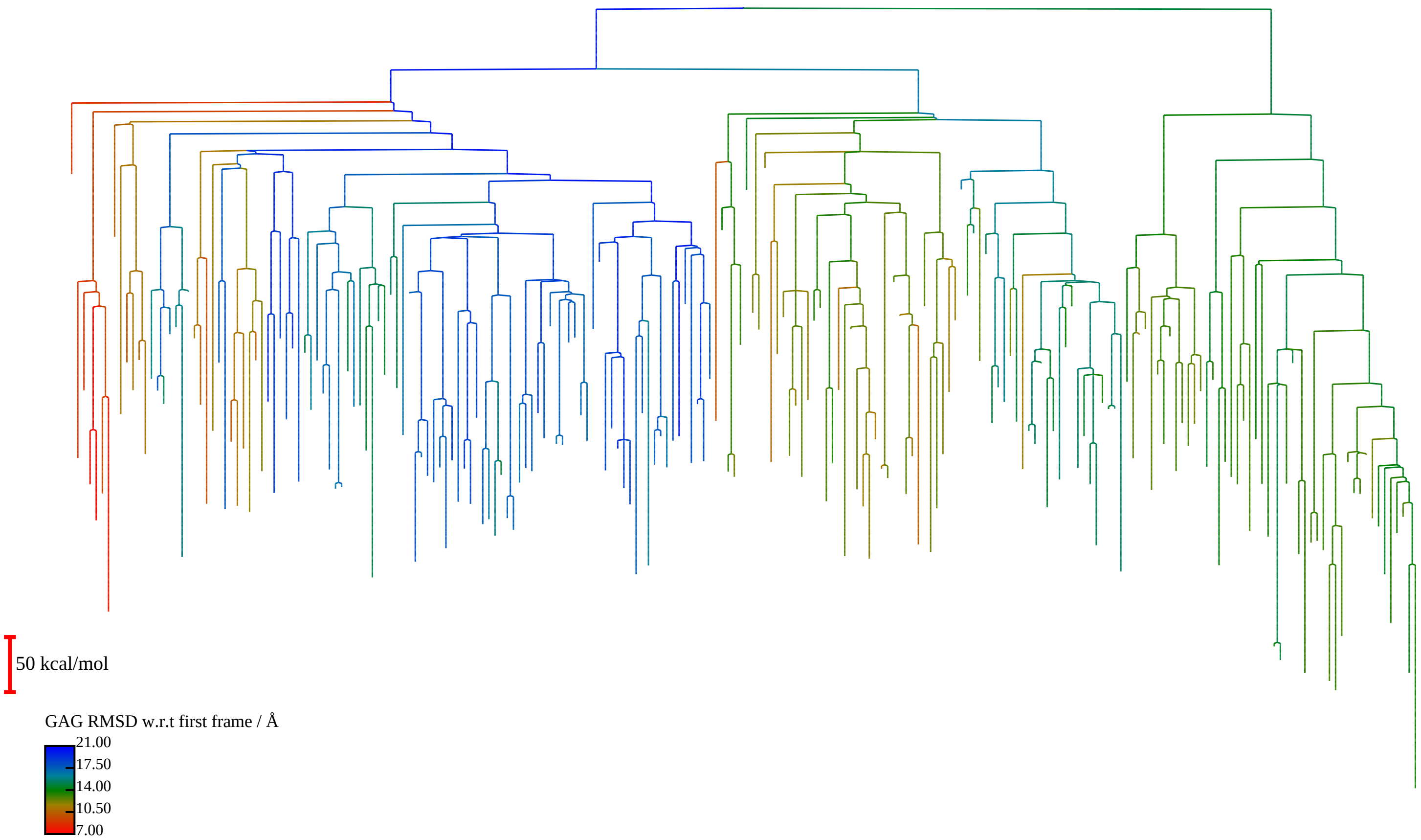

Figure S32: MDDG CHARMM disconnectivity graph for the 4N8W complex.

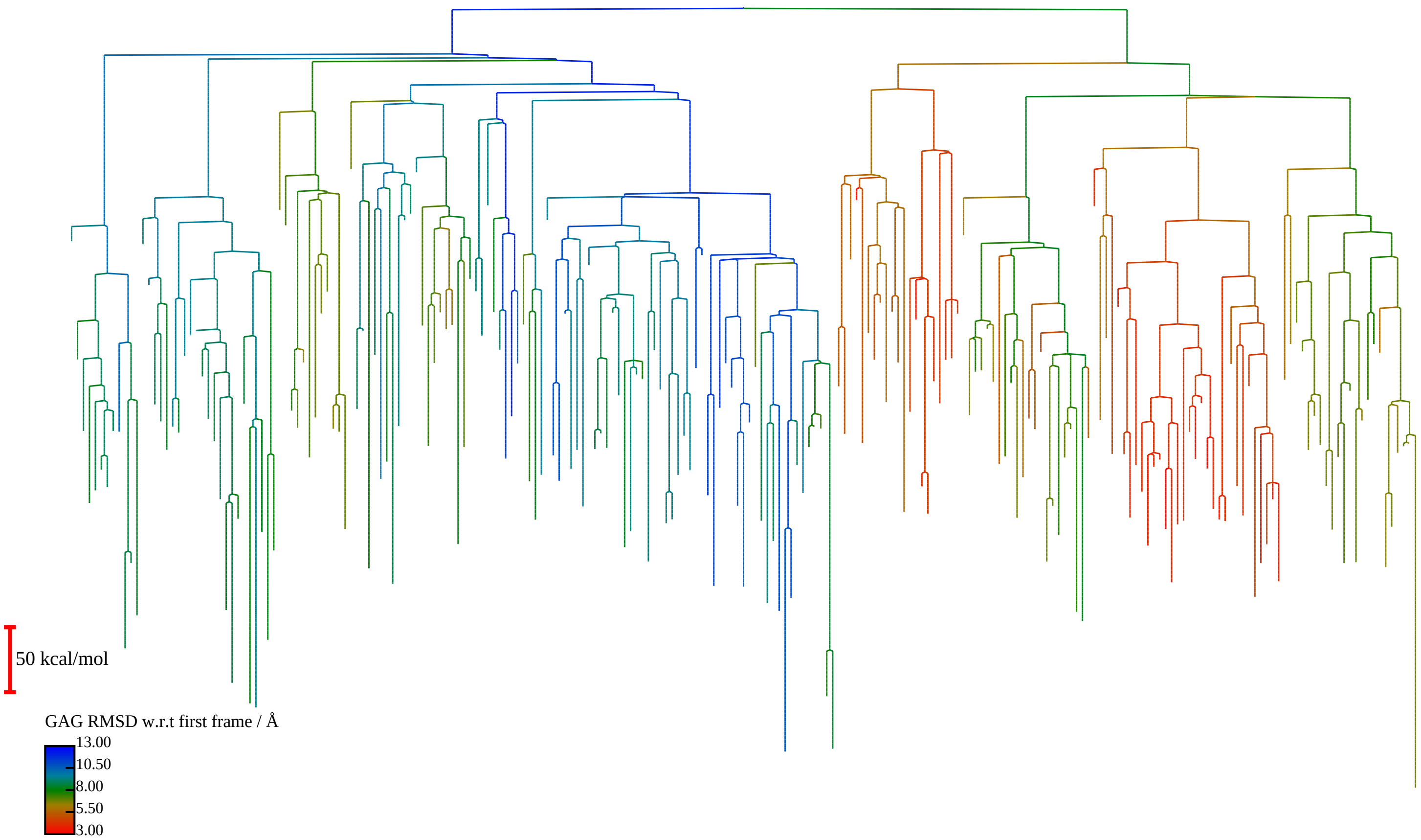

Figure S33: MDDG ff19SB/GLYCAM06j-1 disconnectivity graph for the 1BFC complex.

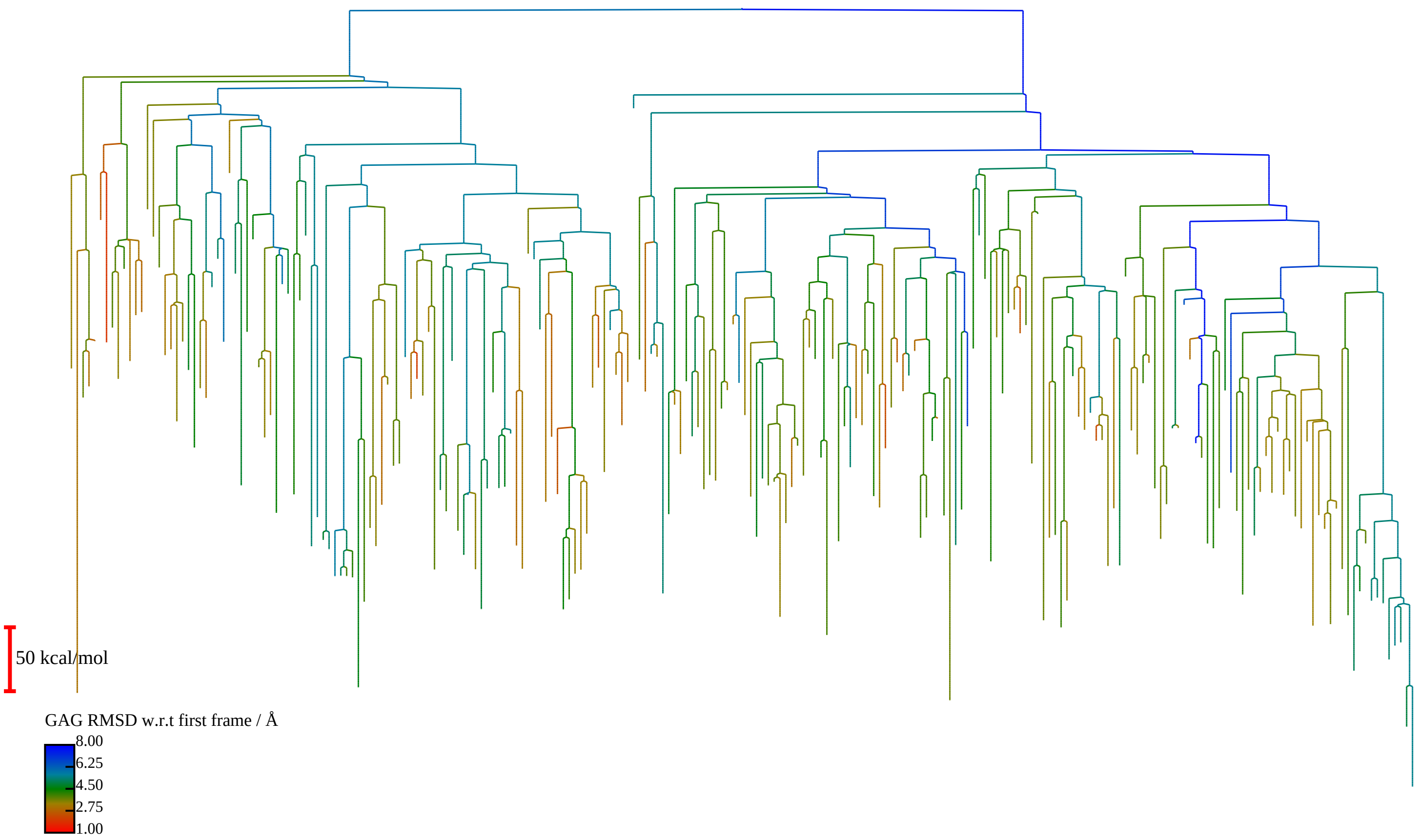

Figure S34: MDDG ff19SB/GLYCAM06j-1 disconnectivity graph for the 2AXM complex.

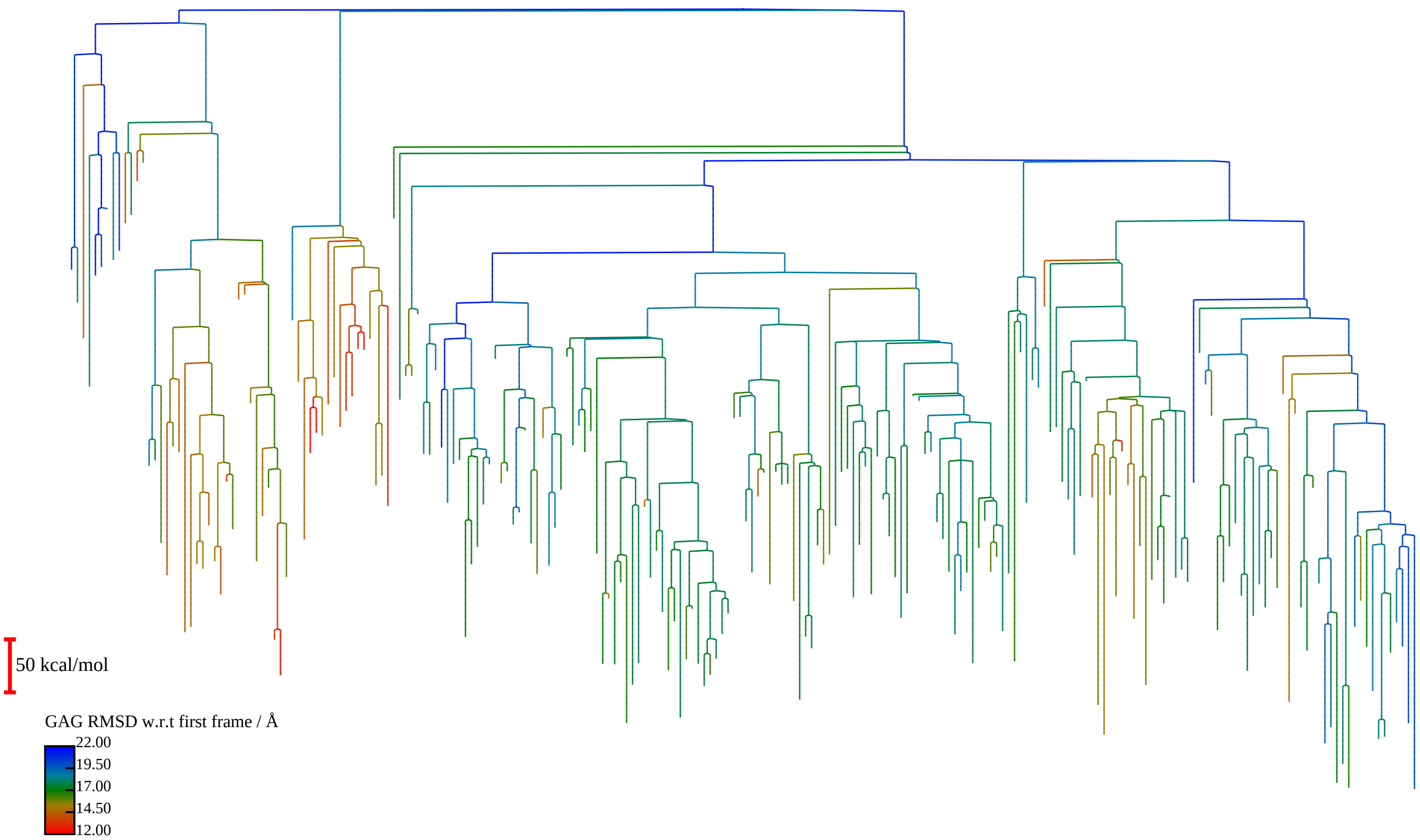

Figure S35: MDDG ff19SB/GLYCAM06j-1 disconnectivity graph for the 4N8W complex.

Table S1: Pearson (r_p) and Spearman (r_s) correlation coefficients between RMSF profiles in the unbound and GAG-bound states for each protein.

**Protein**	**FF14SB**		**CHARMM36m**		**Experimental**	
	r_p	r_s	r_p	r_s	r_p	r_s
FGF-2	0.9609	0.9469	0.6325	0.7648	0.7303	0.6726
FGF-1	0.9402	0.9447	0.9601	0.9400	0.3108	0.2099
CatK	0.9348	0.9707	0.9085	0.9506	0.7585	0.7971

Table S2: Pearson (r_p) and Spearman (r_s) correlation coefficients between RMSF profiles in the unbound and GAG-bound states for each protein for simulations with 15 Å solvent box.

**Protein**	**FF14SB**		**CHARMM36m**		**FF19SB**		**Experimental**	
	r_p	r_s	r_p	r_s	r_p	r_s	r_p	r_s
FGF-2	0.9724	0.9591	0.6059	0.7658	0.9411	0.9530	0.7303	0.6726
FGF-1	0.9872	0.9832	0.9509	0.9276	0.9764	0.9737	0.3108	0.2099
CatK	0.9230	0.9651	0.9205	0.9542	0.9611	0.9793	0.7585	0.7971

Table S3: Pearson (r_p) and Spearman (r_s) correlation coefficients for predicted versus experimental RMSF differences between unbound and complex forms.

**Protein**	**FF14SB**		**CHARMM36m**	
	r_p	r_s	r_p	r_s
FGF-2	0.1068	0.0867	0.3002	0.2555
FGF-1	-0.1219	0.0791	0.4617	0.4804
CatK	-0.1682	-0.2955	-0.1762	-0.1874

Table S4: Pearson (r_p) and Spearman (r_s) correlation coefficients for predicted versus experimental RMSF differences between unbound and complex forms for simulations with 15 Å solvent box.

**Protein**	**FF14SB**		**CHARMM36m**		**FF19SB**	
	r_p	r_s	r_p	r_s	r_p	r_s
FGF2	-0.2053	-0.2236	0.0301	0.0732	0.2128	0.2198
FGF1	0.3696	0.3739	0.4051	0.3742	0.0102	0.0421
CatK	-0.0012	-0.0832	-0.1063	-0.0170	-0.1327	-0.0760
